# C2230, a preferential use- and state-dependent Ca_V_2.2 channel blocker, mitigates pain behaviors across multiple pain models

**DOI:** 10.1172/JCI177429

**Published:** 2024-12-10

**Authors:** Cheng Tang, Kimberly Gomez, Yan Chen, Heather N. Allen, Sara Hestehave, Erick J. Rodríguez-Palma, Santiago Loya-Lopez, Aida Calderon-Rivera, Paz Duran, Tyler S. Nelson, Siva Rama Raju Kanumuri, Bijal Shah, Nihar R. Panigrahi, Samantha Perez-Miller, Morgan K. Schackmuth, Shivani Ruparel, Amol Patwardhan, Theodore J. Price, Paramjit S. Arora, Ravindra K. Sharma, Abhisheak Sharma, Jie Yu, Olga A. Korczeniewska, Rajesh Khanna

**Affiliations:** 1 Department of Molecular Pathobiology, College of Dentistry, and; 2 Pain Research Center, New York University, New York, New York, USA.; 3 The National and Local Joint Engineering Laboratory of Animal Peptide Drug Development, College of Life Sciences, Hunan Normal University, Changsha, China.; 4 Department of Pharmacology and Therapeutics, College of Medicine and; 5 Department of Pharmaceutics, College of Pharmacy, University of Florida, Gainesville, Florida, USA.; 6 Center for Orofacial Pain and Temporomandibular Disorders, Department of Diagnostic Sciences, Rutgers School of Dental Medicine, Newark, New Jersey, USA.; 7 Department of Chemistry, New York University, New York, New York, USA.; 8 University of Texas at Dallas, School of Behavioral and Brain Sciences, Department of Neuroscience, Center for Advanced Pain Studies, Richardson, Texas, USA.; 9 Department of Endodontics, School of Dentistry, University of Texas Health Science Center at San Antonio, and Center for Pain Therapeutics and Addiction Research, San Antonio, Texas, USA.; 10 Department of Anesthesiology and Pain Management, University of Texas Southwestern Medical Center, Dallas, Texas, USA.; 11 Department of Physiology and Aging, College of Medicine, University of Florida, Gainesville, Florida, USA.; 12 Institute of Chinese Medicine Clinical Basics, Institute of Chinese Medicine on Rheumatology, College of Basic Medical Science, Zhejiang Chinese Medical University, Hangzhou, China.; 13 McKnight Brain Institute, University of Florida, Gainsville, Florida, USA.

**Keywords:** Neuroscience, Calcium channels, Pain, Pharmacology

## Abstract

Antagonists — such as Ziconotide and Gabapentin — of the Ca_V_2.2 (N-type) calcium channels are used clinically as analgesics for chronic pain. However, their use is limited by narrow therapeutic windows, difficult dosing routes (Ziconotide), misuse, and overdoses (Gabapentin), as well as a litany of adverse effects. Expansion of novel pain therapeutics may emerge from mechanism-based interrogation of Ca_V_2.2. Here, we report the identification of C2230, an aryloxy-hydroxypropylamine, as a Ca_V_2.2 blocker. C2230 trapped and stabilized inactivated Ca_V_2.2 in a slow-recovering state and accelerated the open-state inactivation of the channel, conferring an advantageous use-dependent inhibition profile. C2230 inhibited Ca_V_2.2 during high-frequency stimulation, while sparing other voltage-gated ion channels. C2230 inhibited Ca_V_2.2 in dorsal root and trigeminal ganglia neurons from rats, marmosets, and humans in a G-protein-coupled-receptor–independent manner. Further, C2230 reduced evoked excitatory postsynaptic currents and excitatory neurotransmitter release in the spinal cord, leading to relief of neuropathic, orofacial, and osteoarthritic pain-like behaviors via 3 different routes of administration. C2230 also decreased fiber photometry-based calcium responses in the parabrachial nucleus, mitigated aversive behavioral responses to mechanical stimuli after neuropathic injury, and preserved protective pain responses, all without affecting motor or cardiovascular function. Finally, site-directed mutation analysis demonstrated that C2230 binds differently than other known Ca_V_2.2 blockers, making it a promising lead compound for analgesic development.

## Abstract 2

We report the discovery of a novel compound (C2230) targeting calcium channels to produce pain relief across various models, including neuropathic, orofacial, and osteoarthritis pain.

## Graphical abstract

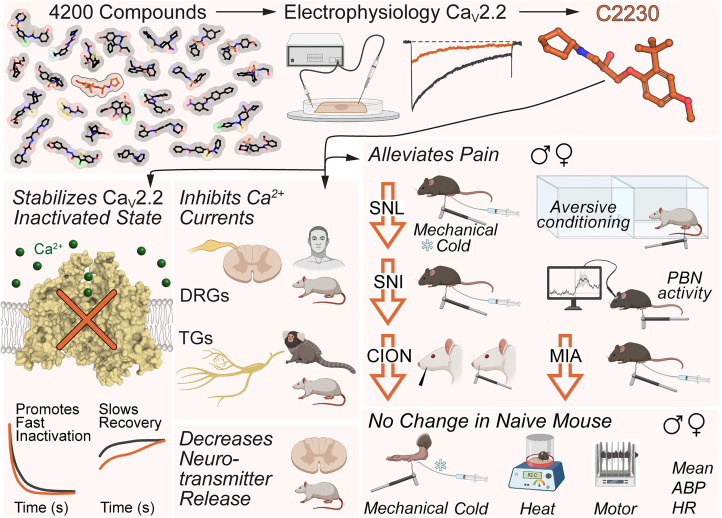

## Introduction

Pain is a multifaceted and debilitating condition that diminishes the quality of life and impacts millions of individuals worldwide. In the US, pain management predominantly relies on nonsteroidal antiinflammatory drugs, conventional opioids, and adjunctive agents like antidepressants and anticonvulsants (1); however, achieving comprehensive relief of pain is challenging due to the aversive side effects frequently associated with these treatments. Researchers have explored various molecular targets to develop better pain treatments. Here, we focus on the voltage-gated Ca_V_2.2 (N-type) calcium channel, a target with documented clinical success.

Ca_V_2.2 channels are expressed in primary afferent neurons and their central terminals in the spinal cord dorsal horn, functioning as critical components in the transmission of pain signals from the periphery to the central nervous system (2). Overexpression and overactivity of these channels causes hyperexcitability and enhanced excitatory neurotransmitter release (3), while blocking Ca_V_2.2 leads to decreased neurotransmitter release and the suppression of pain signals (4). Indeed, studies using knockout mice find that Ca_V_2.2 deficiency reduces pain-like behaviors (5–7), and genetic silencing (7) or pharmacological blockade of these channels alleviates pain (8, 9).

Ziconotide (Prialt) is a synthetic peptide derived from the cone snail peptide ω-conotoxin MVIIA that functions as a selective Ca_V_2.2 inhibitor. Discovered over 40 years ago (10), it holds the distinction of being the first nonopioid intrathecal analgesic approved by the US Food and Drug Administration (FDA) for treating intractable chronic pain (11). However, the effectiveness of Ziconotide is hindered by its limited ability to cross the blood-brain barrier, necessitating intrathecal administration (11). Intrathecal administration of Ziconotide is associated with dizziness and sedation, while systemic administration results in profound hemodynamic effects (12, 13). Advancements targeting Ca_V_2.2 channels have centered around identifying state- and use-dependent Ca_V_2.2 inhibitors. The Snutch group (14) initiated investigations aimed at identifying orally active, selective, state-, and use-dependent inhibitors of N-type calcium channels with a favorable therapeutic index for the treatment of chronic and inflammatory pain. This drug discovery program identified the flunarizine and lomerizine backbones as key contributors to calcium channel blocking activity (14). Subsequent structure-activity relationship studies resulted in the creation of a series of compounds with high affinity for N-type channels, exhibiting IC_50_ values between 10 and 150 nM (14, 15). These compounds demonstrated antiallodynic and antihyperalgesic effects in models of neuropathic and inflammatory pain (16). Z160, the lead compound, despite showing promising preclinical results, failed to demonstrate efficacy in 2 phase II clinical trials for lumbosacral radiculopathy and postherpetic neuralgia. TROX-1, another compound that inhibits N-type channels with an IC_50_ of 0.11 μM, initially showed promise in reversing pain-like behaviors. However, its further development was halted due to motor and cardiovascular impairments (17).

The state- or use-dependency of a drug carries significant implications for advancing therapeutics, especially considering that pain is often linked with hyperexcitability. In our pursuit of identifying state- and use-dependent inhibitors of Ca_V_2.2, we identified 1-(2-tert-butyl-4-methoxyphenoxy)-3-(cyclopentylamino)propan-2-ol, hereafter designated as C2230. This compound inhibited heterologously expressed and native Ca_V_2.2 channels in rats, marmosets, and humans, while also reducing evoked excitatory postsynaptic currents and excitatory neurotransmitter release in the spinal cord. Moreover, it successfully alleviated pain-like behaviors induced by spinal nerve ligation, spared nerve injury, chronic constriction of the infraorbital nerve, and monoiodoacetate-induced osteoarthritis without off-target effects. Site-directed mutation analysis suggests that C2230 binds differently than other known Ca_V_2.2 channel blockers, making it a promising compound for analgesic development. Taken together, we report C2230 as a state- and use-dependent Ca_V_2.2 inhibitor that may offer alternative therapeutic solutions for chronic pain.

## Results

### Identification and characterization of the mechanism of action of C2230, a Ca_V_2.2 (N-type) calcium channel inhibitor.

**Figure 1:**
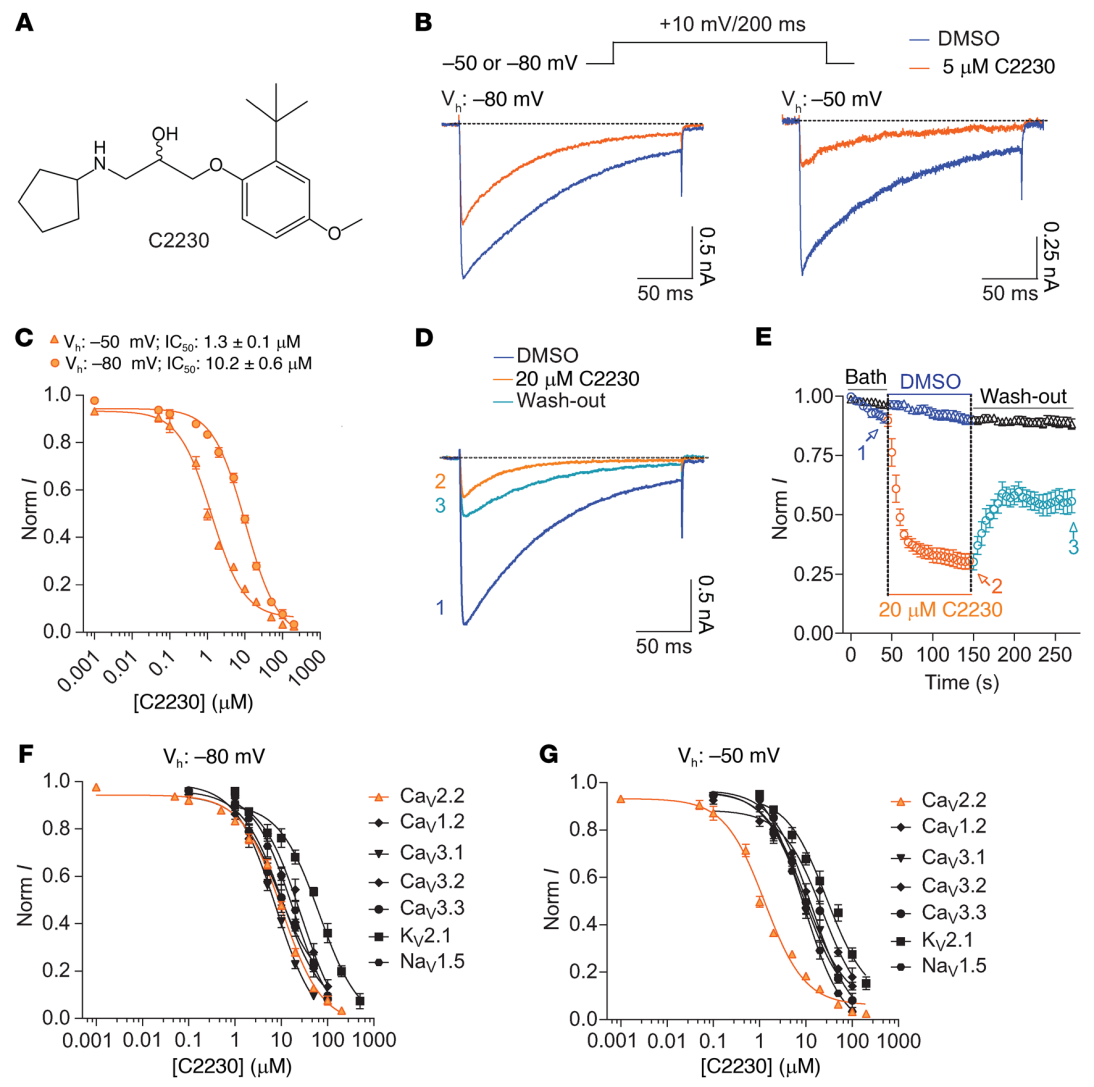
Identification of the aryloxy-hydroxypropylamine compound C2230 as a preferential Ca_V_2.2 channel antagonist. (**A**) Chemical structure of a racemic mixture of C2230. (**B**) Typical current traces from Ca_V_2.2-expressing (*Rattus norvegicus*) HEK293 cells in the presence and absence of 5 μM C2230 at the holding potentials (V_h_) of –50 mV and –80 mV (*n* = 10–16 cells). (**C**) Dose-response relationships of C2230 inhibiting Ca_V_2.2 currents at the 2 V_h_ (*n* = 12–14 cells). (**D** and **E**) Time-course of C2230 inhibiting the Ca_V_2.2 currents and subsequent recovery upon compound washing off (**E**), the typical traces in **D** represent the currents at time points of 1, 2, and 3, as indicated in **E**. Perfusion of 0.1% DMSO served as the negative control (*n* = 7–8 cells). (**F** and **G**) Dose-response relationships of C2230 inhibiting the heterologously expressed K_V_2.1, Na_V_1.5, Ca_V_1.2, Ca_V_3.1, Ca_V_3.2, and Ca_V_3.3 channels, with the IC_50_s being determined as 28.0 ± 5.4 μM and 65.8 ± 12.2 μM for K_V_2.1, 8.7 ± 1.0 μM and 18.1 ± 3.5 μM for Na_V_1.5, 22.7 ± 6.3 μM and 26.9 ± 6.0 μM for Ca_V_1.2, 9.2 ± 1.6 μM and 7.6 ± 0.9 μM for Ca_V_3.1, 9.9 ± 1.7 μM and 8.3 ± 2.5 μM for Ca_V_3.2, and 13.5 ± 2.2 μM and 10.5 ± 1.3 μM for Ca_V_3.3, at the V_h_ of –80 mV (**F**) and –50 mV (**G**), respectively (*n* = 5–9 cells). The Ca_V_2.2 curves were included for comparison. All data are from at least 3 independent experiments.

We screened a structurally diverse compound library (from Selleck Chemicals LLC, Catalog No. L3600) of over 4,200 small molecules for their ability to block heterologously expressed rat Ca_V_2.2 channels using manual patch-clamp electrophysiology. The inhibitory effects of these compounds (10 μM) on Ca_V_2.2 currents were assessed at a holding potential of –80 mV. Compounds showing greater than 50% inhibition were classified as potent Ca_V_2.2 antagonists and designated as positive ‘hits.’ This screening campaign identified C2230 (Figure 1A and Supplemental Table 1; supplemental material available online with this article; https://doi.org/10.1172/JCI177429DS1), belonging to the class of 1-aryloxy-3-amino-2-propanols, as an inhibitor of Ca_V_2.2. Several other inhibitors with lower potency were also found in the compound library (Supplemental Figure 1). Among these, C1740 and C0854 exhibited similar potency to C2230 at a holding potential (V_h_) of –80 mV but were less effective than C2230 at the more depolarizing V_h_ of –50 mV (Supplemental Figure 1).

**Figure 2:**
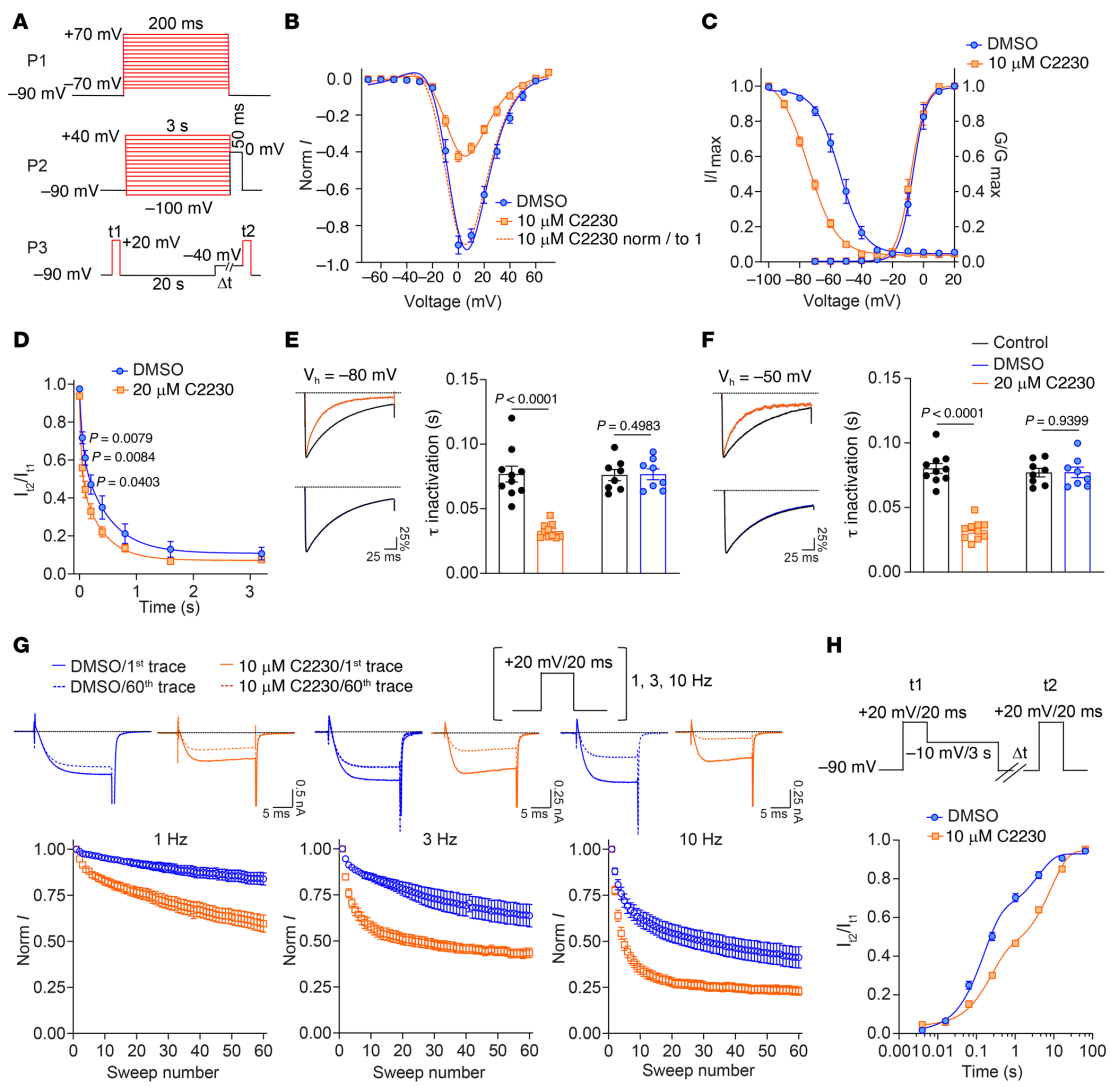
Use- and state-dependent inhibition of Ca_V_2.2 by C2230. (**A**) Voltage protocols assessing activation (P1), steady-state inactivation (P2), the development of time-dependent inactivation (P3) of Ca_V_2.2 channels. (**B**) Ca_V_2.2 current-voltage relationships before (DMSO 0.1%) and after C2230 (10 μM) treatment. Currents in each recording cell were normalized to the maximum peak current before C2230 treatment (blue and orange solid curves) or its own maximum peak current (orange dashed curve) (*n* = 13 cells). (**C**) Steady-state activation and inactivation relationships of Ca_V_2.2 channels in the absence and presence of 10 μM C2230 (*n* = 12 cells). (**D**) Time-dependent development of Ca_V_2.2 channels’ closed-state inactivation in the absence and presence of 20 μM C2230 (*n* = 12–13 cells, *P* values as indicated, Unpaired *t* test). (**E** and **F**) Mean normalized current traces (left) and bar graphs (right) of τ of inactivation (s) at V_h_ of –80 mV (**E**) and –50 mV (**F**) (*n* = 8–10 cells, *P* values as indicated, Paired *t* test). (**G**) Time-dependent current decay of Ca_V_2.2 channels during 60 consecutive step depolarizations at frequencies of 1, 3, and 10 Hz, with and without 10 μM C2230. The upper panel depicts the typical current traces at the first and the 60th depolarization in each group (*n* = 9–11 cells) while the summary data is shown in the lower panels. (**H**) Time-dependent recovery of Ca_V_2.2 channels from inactivated state as evaluated using the voltage protocol (upper panel; *n* = 14 cells) with the time constants for fast recovery (τ_fast_) and slow recovery (τ_slow_) being increased from 0.151 ± 0.022 s to 0.232 ± 0.026 s, and 5.719 ± 1.079 s to 9.382 ± 1.079 s by C2230 treatment, respectively (*P* < 0.05 for both τ_fast_ and τ_slow_ comparisons, Mann-Whitney test). The proportion of fast recovering channels were reduced in the C2230 group compared with that in the DMSO group (66.7 ± 3.0% to 42.4 ± 2.0%; *P* < 0.001; Mann-Whitney test). All data are from at least 3 independent experiments. See Supplemental Table 3 for full statistical analysis.

We next synthesized and purified C2230 as a racemic mixture to homogeneity for further in-depth characterization. Acute application of C2230 (5 μM, example traces from a low subsaturating concentration are shown to illustrate the difference at the 2 V_h_) demonstrated robust and rapid inhibition of Ca_V_2.2 currents (Figure 1B). C2230 block of Ca_V_2.2 was greater at –50 mV when compared with –80 mV (Figure 1B), with half-maximal inhibitory concentration (IC_50_) values of 1.3 ± 0.1 μM and 10.2 ± 0.6 μM, respectively (Figure 1C). These findings imply a use-dependent inhibition of C2230 on Ca_V_2.2, with higher inhibitory potency observed at –50 mV. At this V_h_, Ca_V_2.2 channels more readily transition into the inactivated state, unlike at –80 mV, where the resting state of the channels is dominant (see steady-state inactivation curve in Figure 2C). Acute application of a subsaturating concentration of C2230 (20 μM) exhibited a fast onset and stable inhibition of Ca_V_2.2 currents, with apparent inhibitory and recovery time constant values of 12.3 ± 1.7 seconds and 9.9 ± 1.3 seconds, respectively (Figure 1, D and E). However, the inhibition was only partially reversed, even with prolonged bath solution washing (Figure 1E). Perfusion with 0.1% DMSO did not affect the currents (Figure 1E).

We next assessed the effect of C2230 on other ion channels, including voltage-gated potassium, sodium, and non-Ca_V_2.2 calcium channels in heterologous systems. At the V_h_ of –80 mV, C2230 (20 μM) inhibited K_V_2.1 and Na_V_1.5 channels by approximately 45% (Supplemental Figure 2). Similarly, C2230 inhibited both L-type (Ca_V_1.2-1.3) and T-type (Ca_V_3.1-3.3) calcium channels by between 31% to 65% (Supplemental Figure 2). The other channels, including K_V_1.3, K_V_1.5, K_V_3.1, K_V_3.2, K_V_3.4, K_V_4.1–4.3, Na_V_1.3, Na_V_1.4, and Na_V_1.7–1.9 were more resistant to block by C2230 (Supplemental Figure 2). We further analyzed the concentration-dependent inhibition by C2230 of K_V_2.1, Na_V_1.5, Ca_V_1.2, and Ca_V_3.1-3.3 channels at the V_h_ of –50 mV and –80 mV (Figure 1, F and G). C2230 showed only slight variations in the inhibition of Na_V_1.5 and K_V_2.1 channels at the V_h_ of –50 and –80 mV (Figure 1, F and G). Across all concentrations tested, C2230 exhibited consistent inhibitory effects of Ca_V_1.2 and Ca_V_3.1–3.3 channels at both V_h_ (Figure 1, F and G), in contrast to the preferential inhibition of Ca_V_2.2 at –50 mV (Figure 1C). We determined the IC_50_s of C2230 inhibition of Ca_V_1.2, Ca_V_3.1, Ca_V_3.2, Ca_V_3.3, Na_V_1.5, and K_V_2.1 channels at the V_h_ of –50 mV (facilitating channel entering the inactivated state) and –80 mV (stabilizing channel in the resting state) and normalized these IC_50_s to the IC_50_ value of C2230 inhibiting Ca_V_2.2. These results show that C2230 preferentially inhibits Ca_V_2.2 channels, with the selectivity being less pronounced at –80 mV (Figure 1F and Supplemental Figure 3), compared with other tested channels, than at the V_h_ of –50 mV (Figure 1G and Supplemental Figure 3). These data suggest that C2230 is a use-dependent, preferential Ca_V_2.2 channel antagonist.

### C2230 enhances the closed-state inactivation, accelerates the open-state inactivation, and inhibits Ca_V_2.2 channels during high-frequency stimulation.

The enhanced inhibition of Ca_V_2.2 currents by C2230 at more depolarized V_h_ (–50 mV) suggests that the compound preferably binds to inactivated channels. To investigate this further, we tested the effects of C2230 on the gating kinetics of heterologously expressed Ca_V_2.2 channels. The current-voltage (I-V) relationships, established through the P1 protocol (Figure 2A), show that C2230 (10 μM) inhibited Ca_V_2.2 currents (blue versus orange solid curves, Figure 2B), without affecting the shape of the I-V curve, and, thus, the threshold activation voltage, the peak activation voltage, and the reversal activation voltages (blue solid versus orange dashed curves, Figure 2B). This resulted in an unaffected steady-state conductance-voltage (G-V) relationship in C2230-treated cells (Figure 2C). These data suggest that C2230 does not function by affecting the voltage-dependent activation of Ca_V_2.2. On the other hand, C2230 induced an approximately 20 mV hyperpolarizing shift in the voltage-dependence of steady-state inactivation (Figure 2C), which was evaluated through a 2-pulse protocol (P2, Figure 2A). These findings suggest that C2230 trapped the inactivated channels induced by the conditional pulses, thereby reducing the availability of channels during the subsequent test pulse. Such use-dependent inhibition of ion channels by their antagonists has been commonly observed in previous studies (18) and can be explained as a regulated access mechanism of the ligand to its binding site (4).

To delve deeper into the kinetics of C2230 trapping inactivated Ca_V_2.2 channels, we employed a 3-pulse protocol (P3, Figure 2A). A conditional pulse of –40 mV with varying durations was utilized to induce closed-state inactivation of the channels, while short depolarization test pulses (t1 and t2) were used to assess the available channels before and after the conditional pulse, respectively. The results revealed that C2230 rapidly trapped and stabilized the inactivated channels, apparently outpacing the development of fast inactivation itself (Figure 2D). Next, we examined the compound’s effect on the open-state inactivation (OSI) of Ca_V_2.2 channels. Our findings revealed that C2230, but not 0.1% DMSO, significantly accelerated OSI, as evidenced by a marked reduction in the time required for the channels to inactivate following depolarization after compound treatment, observed at V_h_ of –80 mV and –50 mV (Figure 2, E and F). This effect is likely due to C2230 rapidly binding to and blocking the open-state channels prior to inactivation.

C2230’s rapid, preferential binding to the inactivated state is advantageous during elevated neuronal firing in pain. Notably, when compared with the DMSO group, C2230 exhibited inhibition of Ca_V_2.2 currents during high-frequency stimulation at 1, 3, and 10 Hz (Figure 2G). Furthermore, these inactivated and C2230-blocked channels displayed a slower recovery to the closed and activatable states compared with their compound-free counterparts during hyperpolarization to –90 mV (Figure 2H). Overall, our findings suggest that C2230 binds a high-affinity inactivation site in Ca_V_2.2, accelerating OSI and trapping channels inactivated with slower recovery, making it a strong inhibitor during heightened neuronal activity.

### C2230 inhibits Ca_V_2.2 currents without affecting neuronal excitability in rat DRG neurons.

**Figure 3:**
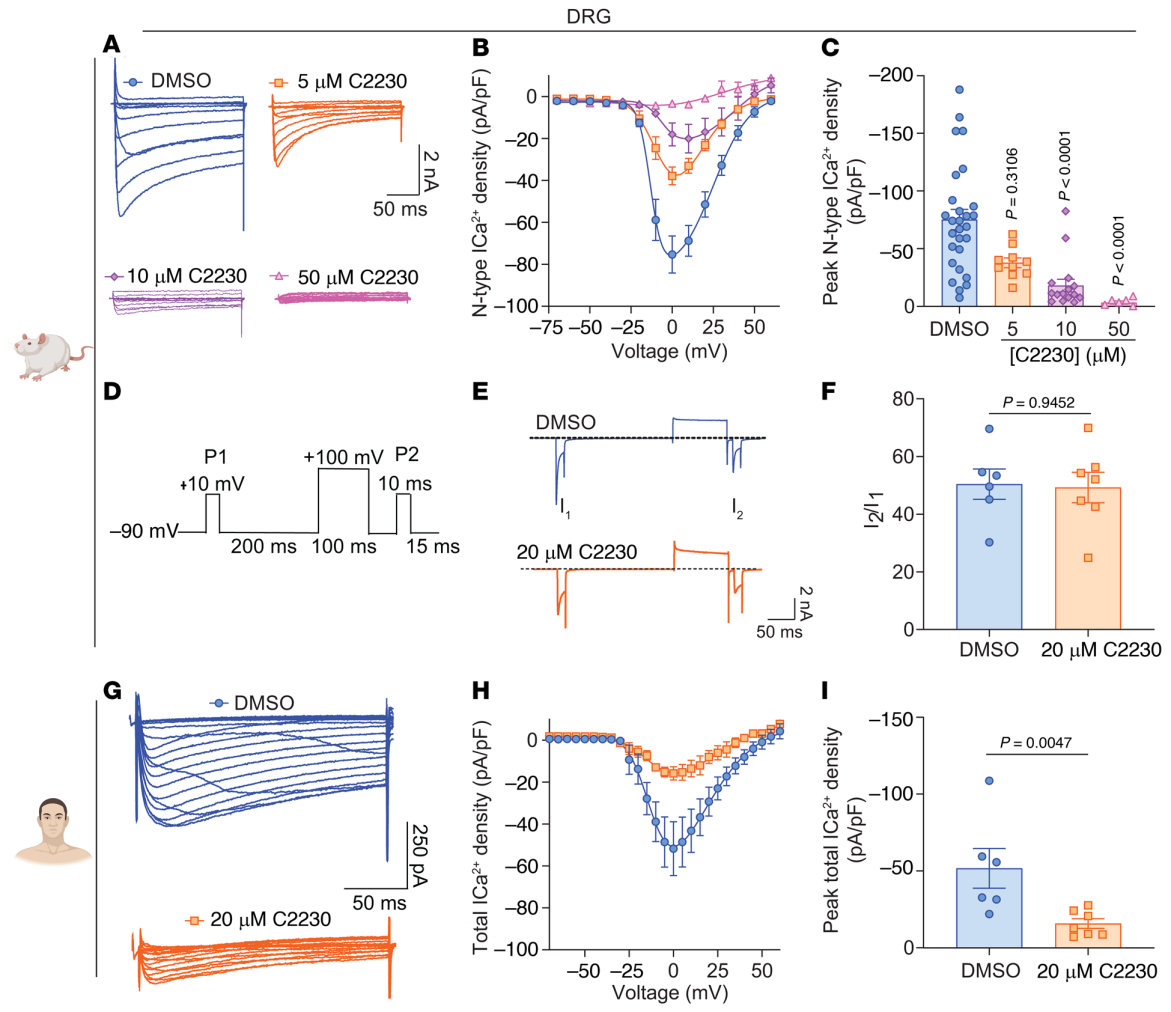
C2230 inhibits Ca_V_2.2 (N-type) calcium currents and total calcium currents in rat and human dorsal root ganglia sensory neurons, respectively. (**A**) Representative traces of N-type calcium currents from rat dorsal root ganglion (DRGs) neurons incubated with 0.1% DMSO (control; blue circles), 5 μM C2230 (orange squares), 10 μM C2230 (purple diamonds) and 50 μM C2230 (pink triangles). (**B**) Summary of N-type ICa^2+^ current density-voltage relationship. (**C**) Bar graphs of peak N-type ICa^2+^ density from rat DRGs pretreated as indicated. *P* values as indicated, Kruskal-Wallis test followed by Dunn’s post hoc test, *n* = 6–28 cells per condition from 3 independent experiments. (**D**) Paired-pulse voltage protocol for evaluating the possible GPCR-mediated inhibition on Ca_V_ currents, in which the +100 mV/100 ms strong depolarization was used to drive Gβγ dissociation from the Ca_V_ channels. (**E**) Typical DRG total Ca_V_ current traces elicited by the paired-pulse voltage protocol in (**D**), in the absence (DMSO) or presence of 20 μM C2230. (**F**) Summary I_2_/I_1_ ratio in (**E**) (*P* values as indicated, Mann-Whitney test *n* = 6–7 from 2 independent experiments). (**G**) Representative traces of total calcium currents from human DRGs incubated with 0.1% DMSO (control; blue circles) or 20 μM C2230 (orange squares). (**H**) Summary of total ICa^2+^ current density-voltage relationship. (**I**) Bar graphs of peak total ICa^2+^ density from human DRGs pretreated as indicated. *P* values as indicated, Mann-Whitney test, *n* = 6–7 cells per condition from 1 independent experiment. Error bars indicate mean ± SEM. See Supplemental Table 3 for full statistical analysis.

Small dorsal root ganglion (DRG) neurons, where Ca_V_2.2 channels reside (2), typically exhibit resting membrane potentials within the range of –40 to –60 mV (19, 20). Given our observed heightened inhibition of Ca_V_2.2 channels at a V_h_ of –50 mV (Figure 1C), our findings suggest that C2230 exhibits a preference for inhibiting Ca_V_2.2 over other voltage-gated ion channels. To test this, we isolated Ca_V_2.2 currents in DRGs through the application of a calcium channel blocker cocktail (21). Subsequently, we treated DRG neurons with different concentrations of C2230 or 0.1% DMSO, by adding the compound to the external recording solution for the entire duration of the recordings. C2230 at all tested concentrations attenuated Ca_V_2.2 currents (Figure 3A) and current densities (Figure 3B), while 50 μM nearly abolished these currents (Figure 3, A and B) across voltages ranging from –20 mV to +50 mV. Peak current analysis showed a significant reduction in calcium current densities at 10 and 50 μM of C2230 (Figure 3C). Notably, C2230 (20 μM) did not alter the resting membrane potential (RMP), rheobase, action potential firing, or fast after-hyperpolarization (fAHP, Supplemental Figure 4), suggesting that the compound does not impact neuronal excitability.

Since α conotoxins inhibit Ca_V_2.2 via G-protein coupled receptors (GPCRs) (22), we tested if C2230 does as well. G-βγ subunits physically interact with voltage-gated calcium channels (23, 24), stabilizing them in a closed state, requiring strong depolarizations to dissociate. Using a paired-pulse protocol (Figure 3D) we found no evidence of GPCR-mediated inhibition of Ca_V_2.2 with 20 μM C2230, as shown by the unchanged I_2_/I_1_ ratio (Figure 3, E and F). Thus, C2230 does not activate inhibitory GPCR signaling.

### C2230 inhibits total calcium currents in human DRG neurons.

Certain compounds can exhibit species-specific effects. What proves effective in rodents may not necessarily translate to humans (25). Therefore, given our prior observations of C2230 attenuating Ca_V_2.2 currents in rodents, we next assessed if C2230 inhibited calcium currents in human DRG neurons (see demographics of donors in Supplemental Table 2). Our findings revealed that C2230 (20 μM) led to a reduction in total calcium currents (Figure 3G) as well as current densities (Figure 3H). Peak current density analysis showed a statistically significant reduction in current with C2230 compared with control (Figure 3I). Importantly, these findings bridge the gap between rodent and human and provide compelling evidence that C2230 holds promise as a Ca_V_2.2 channel inhibitor in human DRG neurons.

### C2230 inhibits total calcium currents in rat and marmoset TG neurons.

**Figure 4:**
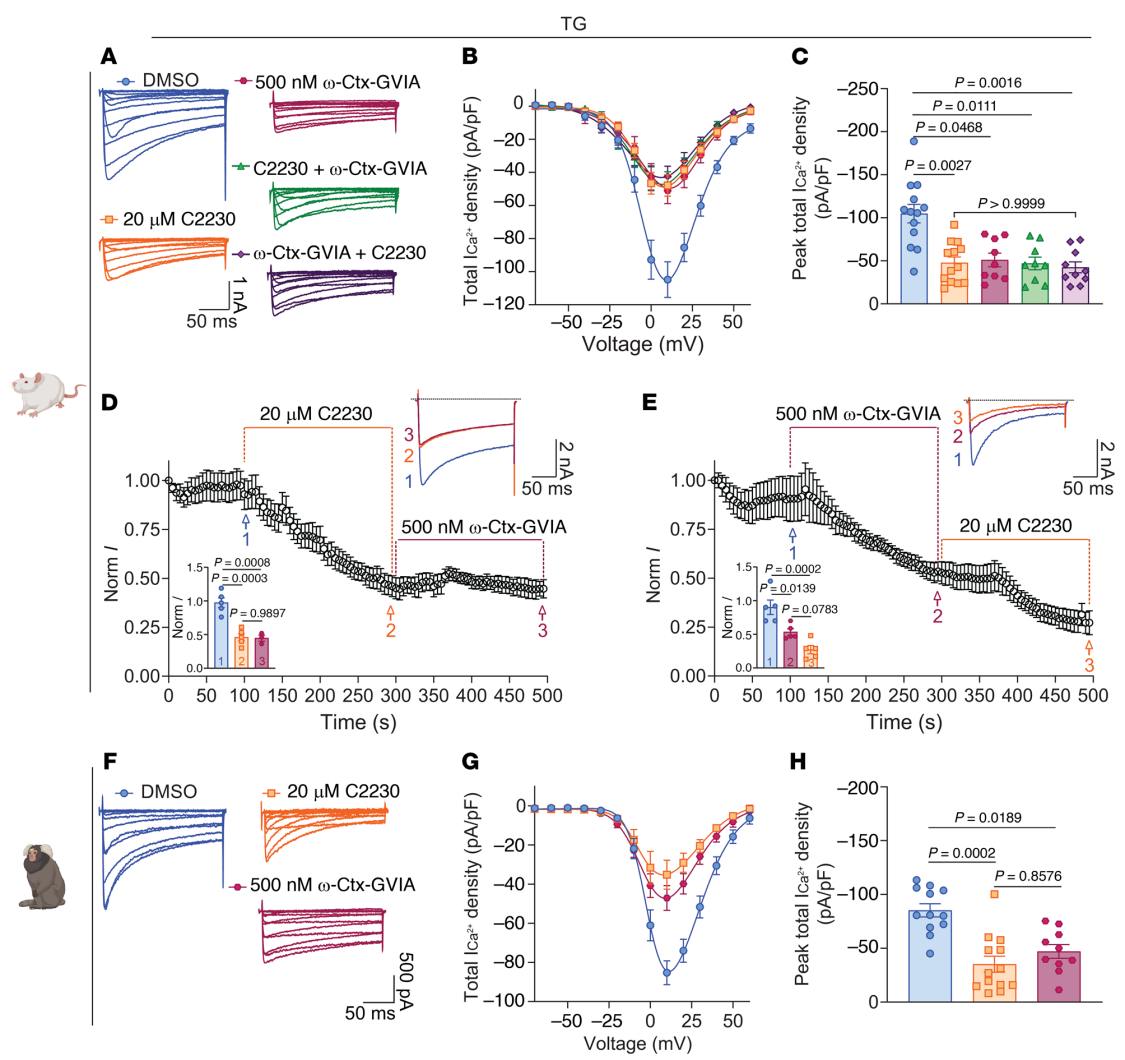
C2230 inhibits total calcium currents in rat and marmoset trigeminal sensory neurons. (**A**) Representative traces of total calcium currents from rat trigeminal ganglia (TG) neurons treated with 0.1% DMSO (control; blue), 20 μM C2230 (orange), 500 nM ω-conotoxin-GVIA (ω-Ctx-GVIA; burgundy), C2230 + ω-Ctx-GVIA (green), or ω-Ctx-GVIA + C2230 (dark purple). (**B**) Summary of total ICa^2+^ density-voltage relationship. (**C**) Bar graphs of peak total ICa^2+^ density from rat TGs -treated as indicated. *P* values as indicated, Kruskal-Wallis test followed by Dunn’s post hoc test, *n* = 9–13 cells per condition from 3 independent experiments. (**D**) Time-course of Ca_V_2.2 current inhibition by sequential perfusion of C2230 and ω-Ctx-GVIA. Inset: Bar graph illustrating the normalized current (Norm *I*) of each condition at the indicated time points. *P* values as indicated, 1-way ANOVA followed by Tukey multiple comparison test, *n* = 3–5 cells per condition from 2 independent experiments. (**E**) Time-course of Ca_V_2.2 currents inhibition by sequential perfusion of ω-Ctx-GVIA and C2230 perfusion. Inset: Bar graph illustrating the normalized current (Norm *I*) at the time points as indicated. *P* values as indicated, 1-way ANOVA followed by Tukey multiple comparison test, *n* = 5 cells per condition from 2 independent experiments. (**F**) Representative traces of total calcium currents from marmoset TGs incubated with 0.1% DMSO (control; blue circles), 20 μM C2230 (orange squares), or 500 nM ω-Ctx-GVIA (burgundy hexagons). (**G**) Summary of total ICa^2+^ density-voltage relationship. (**H**) Bar graphs of peak total ICa^2+^ density from marmoset TGs pretreated as indicated. *P* values as indicated, Kruskal-Wallis test followed by Dunn’s post hoc test, *n* = 10–13 cells per condition from 1 independent experiment. Error bars indicate mean ± SEM. See Supplemental Table 3 for full statistical analysis.

Ca_V_2.2 channels are expressed in adult rat trigeminal ganglion (TG) neurons, where they are implicated in the modulation of cephalic pain (21, 26). To test if C2230 inhibits calcium currents in rat sensory neurons innervating the head and face, we isolated TG neurons and measured total calcium currents (Figure 4, A–E). Acute application of C2230 (20 μM) resulted in a reduction of Ca^2+^ currents (Figure 4A) and current densities (Figure 4B). The C2230-mediated decrease in current densities was indistinguishable from those of DRGs treated with ω-conotoxin-GVIA (500 nM; Figure 4, B and C), a well-known and selective Ca_V_2.2 blocker (27). Next, we applied C2230 to a group of neurons, followed by ω-conotoxin-GVIA, while in another group, ω-conotoxin-GVIA was applied first, followed by C2230. These occlusion experiments revealed that total Ca^2+^ current densities did not further diminish, regardless of the order in which the Ca_V_2.2 blockers were applied (Figure 4, B and C). In a separate set of experiments, TG neurons were perfused with C2230 first, followed by ω-conotoxin-GVIA (Figure 4D), showing that once Ca_V_2.2 channels were blocked by C2230, total Ca^2+^ currents were not further reduced by subsequent ω-conotoxin-GVIA perfusion. A similar outcome was observed when the sequence of Ca_V_2.2 blocker perfusion was reversed (Figure 4E). Collectively, these data confirm that both compounds target Ca_V_2.2 channels.

To further corroborate our observations in a nonhuman primate model, we obtained TGs from *Callithrix jacchus* (marmoset). As with the rat TGs, total calcium currents (Figure 4F), current densities (Figure 4G), and peak currents (Figure 4H) were significantly reduced by C2230 (20 μM), when compared with DMSO-treated cells. Notably, this reduction was similar to that of ω-conotoxin-GVIA (500 nM; Figure 4H). These results demonstrate that C2230 is an effective Ca_V_2.2 inhibitor in TG neurons from both rodent and nonhuman primates.

### C2230 decreases rat spinal neurotransmission.

**Figure 5:**
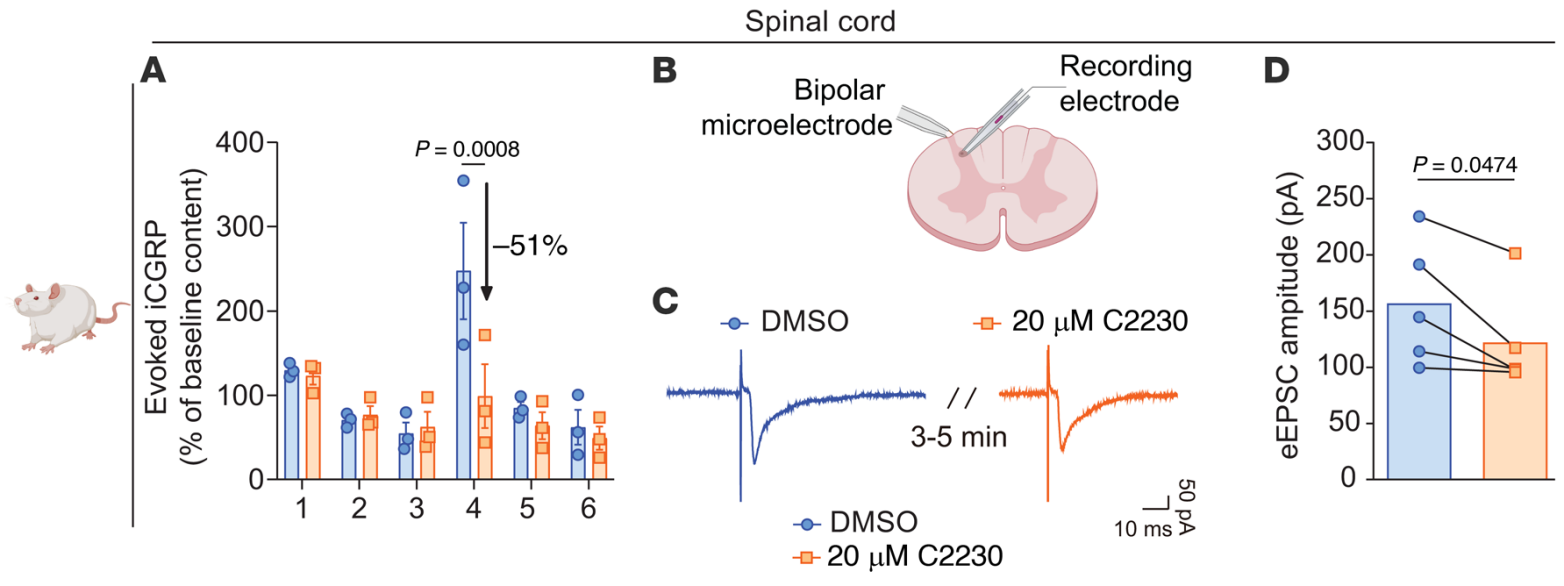
C2230 decreases spinal cord neurotransmission. (**A**) KCl (90 mM) depolarization-evoked immunoreactive calcitonin gene-related peptide (iCGRP) release was measured from spinal cords isolated from naive female rats following 10 minutes preincubation with 0.1% DMSO (control) or 20 μM of C2230. Bar graph showing iCGRP levels observed in bath solution normalized to the weight of each spinal cord section. Fraction 1, Baseline 1 measurement; Fraction 2, Baseline 2 measurement; Fraction 3, Treatment with vehicle and C2230; Fraction 4, Treatment with vehicle and C2230 + 90 mM KCl; Fraction 5, Wash 1; Fraction 6, Wash 2. *P* value as indicated; 2-way ANOVA with Šidák’s multiple comparisons test; *n* = 3 rats. (**B**) Cartoon representation of the electrophysiology setup used to measure evoked excitatory postsynaptic currents (eEPSCs) in spinal cord slices. A stimulus (~200 μA, 0.1 ms) was applied to the tract of Lissauer via a bipolar microelectrode connected to a flexible stimulus isolator. eEPSCs were recorded from neurons located in the substantia gelatinosa (lamina I/II). (**C**) Representative traces of eEPSCs recorded in the presence of 0.1% DMSO (control) or 20 μM of C2230. (**D**) Bar graph showing the amplitude of eEPSCs for these 2 conditions. *P* value as indicated; paired *t* test; *n* = 5 cells from 1 independent experiment. Data are expressed as mean ± SEM. See Supplemental Table 3 for full statistical analysis.

Ca_V_2.2 channels in primary afferent terminals are essential for dorsal horn neurotransmission (28). To investigate the impact of C2230 on the release of the excitatory neurotransmitter calcitonin gene-related peptide (CGRP) (29), we exposed rat lumbar spinal cords to depolarization with 90 mM KCl and measured evoked iCGRP levels using enzyme-linked immunosorbent assay. C2230 (20 μM) reduced iCGRP release by approximately 51% compared with controls (Fraction 4; Figure 5A). These results suggest that C2230 inhibits Ca_V_2.2 channels to reduce CGRP release.

Next, to assess whether C2230 modulates synaptic transmission at excitatory synapses, we recorded evoked excitatory postsynaptic currents (eEPSCs) in spinal cord slices. We stimulated the tract of Lissauer via a bipolar microelectrode and recorded eEPSCs from neurons in the substantia gelatinosa (lamina I/II) (Figure 5B). Perfusion with C2230 (20 μM) resulted in a significant reduction in the eEPSCs amplitude (156.9 ± 24.91 pA to 122.2 ± 20.15 pA, *n* = 5, *P* < 0.05), compared with the control group (0.1% DMSO; Figure 5, C and D). These results suggest that C2230 diminishes the strength of synaptic transmission, likely by inhibiting presynaptic calcium influx through Ca_V_2.2 channels.

### C2230 reverses nociceptive behaviors in rodents with neuropathic pain.

**Figure 6:**
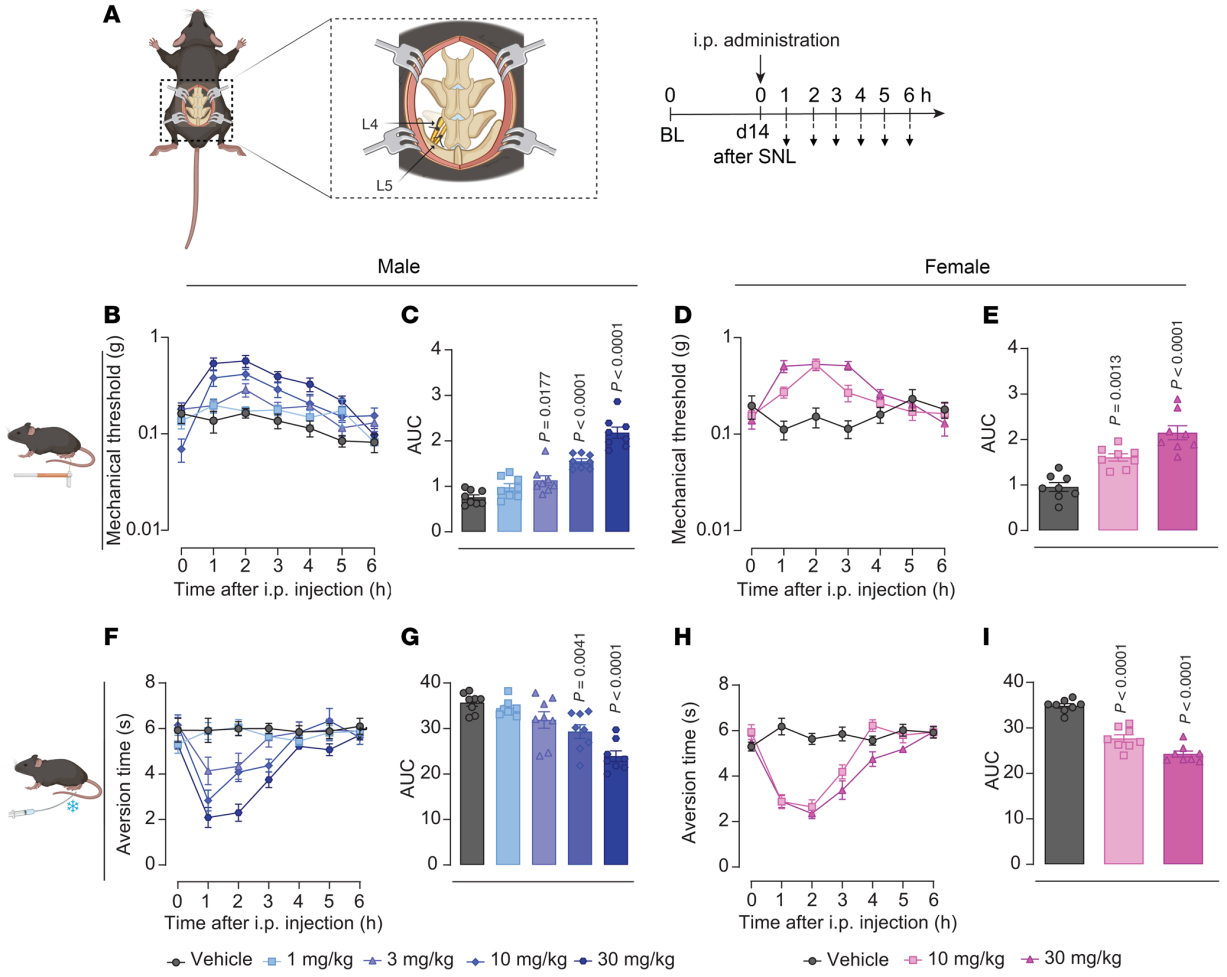
Intraperitoneal administration of C2230 induces reversal of pain-like behaviors induced by spinal nerve ligation in male and female mice. (**A**) Spinal nerve ligation (SNL) model schematic and timeline of the experimental approach used to determine the antinociceptive effects induced by C2230 on tactile and cold allodynia. Dose-response curves of the paw withdrawal mechanical threshold measurements after i.p. administration of vehicle or C2230 in male (**B**) and female (**D**) mice; *n* = 8 mice per group. Quantification of the area under the curve (AUC) of panels **B** and **D** between the baseline and 6 hours after i.p. injection in male (**C**) and female (**E**) mice, respectively. *P* values as indicated by 1-way ANOVA followed by Dunnett post hoc test; *n* = 8 mice per group. Dose-response curves of the aversion time to acetone stimulation after vehicle or C2230 i.p. administration in male (**F**) and female (**H**) mice. Quantification of the AUC of **F** and **H** between baseline and 6 hours after i.p. injection in male (**G**) and female (**I**) mice. *P* values as indicated; 1-way ANOVA followed by Dunnett post hoc test; *n* = 8 mice per group; values are expressed as mean ± SEM. See Supplemental Table 3 for full statistical analysis.

Ca_V_2.2 inhibition and the subsequent reduction in spinal neurotransmission by C2230 prompted us to evaluate its potential antinociceptive effects in preclinical models of pain. Given the established influence of sex on the antinociceptive effects of various drugs (30), we conducted behavioral experiments in both male and female rodents. Firstly, we evaluated C2230 in the L4/L5 spinal nerve ligation (SNL) (31) model of neuropathic pain in mice (Figure 6A). SNL resulted in the development of mechanical allodynia in male (Figure 6, B and C) and female (Figure 6, D and E) mice 14 days after injury (0 timepoint). Intraperitoneal administration of C2230 (1–30 mg/kg) dose-dependently mitigated SNL-induced mechanical allodynia and increased the AUC in male (Figure 6, B and C) and female (Figure 6, D and E) mice when compared with vehicle-treated mice, with an effective dose for 20% of the population (ED_20_) of 4.9 mg/kg in males (Supplemental Figure 5). Intrathecal administration of C2230 also reversed L5/L6 SNL-induced mechanical allodynia in rats (Supplemental Figure 6). Similarly, C2230 exhibited a dose-dependent reversal of SNL-induced cold allodynia and decreased the AUC in males (Figure 6, F and G) and females (Figure 6, H and I) when compared with vehicle-treated mice, with an ED_30_ of 6.7 mg/kg in males (Supplemental Figure 5B).

**Figure 7:**
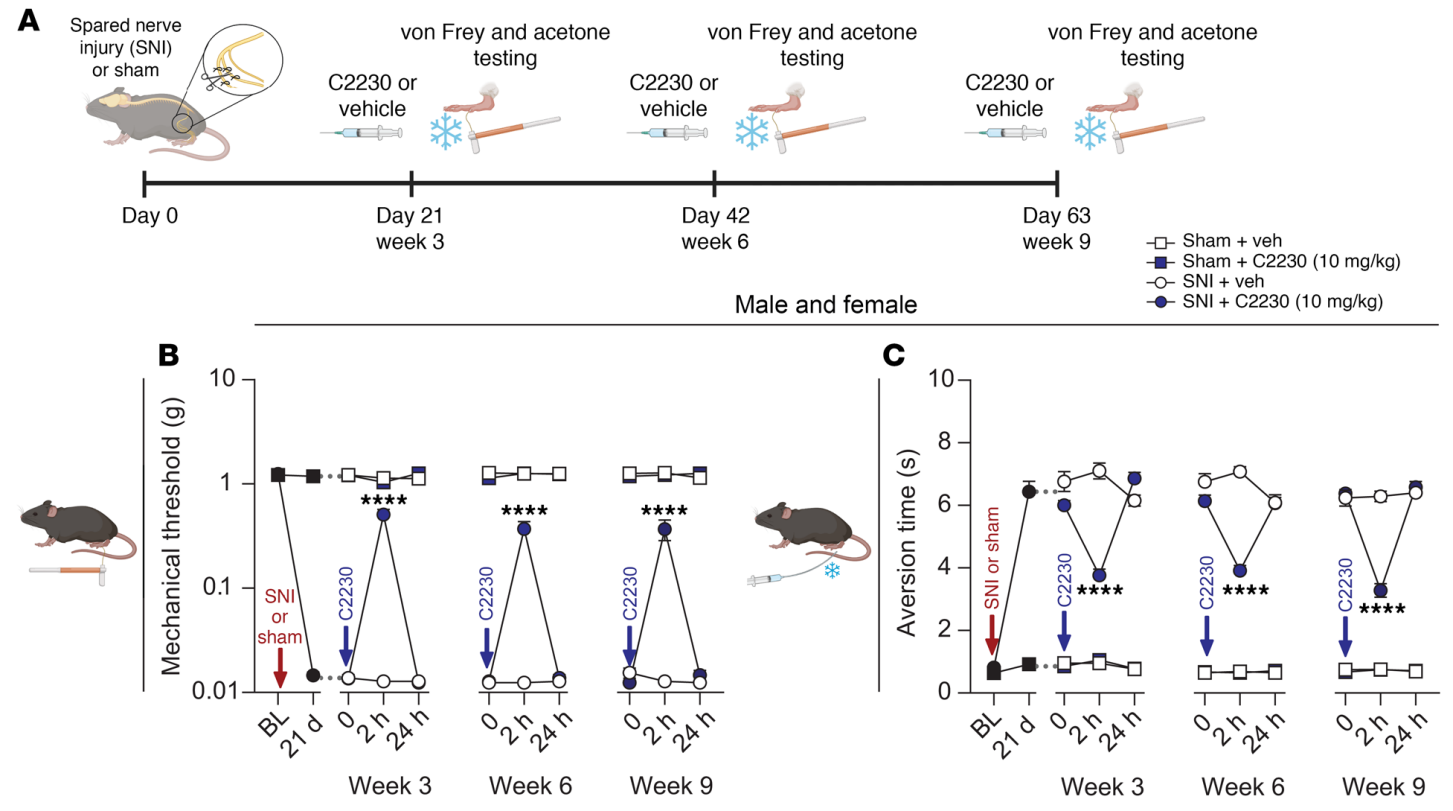
Repeated administration of C2230 maintains long-term efficacy in alleviating neuropathic pain–like behavior without the development of tolerance. (**A**) Timeline for spared nerve injury (SNI) or sham surgery and repeated administration of C2230 (i.p.; 10 mg/kg). (**B**) Time course of von Frey mechanical thresholds after i.p. administration of vehicle or C2230. C2230 is efficacious at alleviating SNI-induced mechanical hypersensitivity at 3- 6- and 9-week timepoints. (**C**) Time course of aversion time responses after i.p. administration of vehicle or C2230. C2230 is efficacious at alleviating SNI-induced cold hypersensitivity at 3- 6- and 9-week timepoints; *n* = 8–10 mice/group. 2-way repeated measures (RM) ANOVA with Dunnnet’s post hoc test. Data are shown as means ± SEM. ****P < 0.0001. See Supplemental Table 3 for full statistical analysis.

Having established the in vivo efficacy of C2230 in reducing behavioral correlates of neuropathic pain, we next evaluated the ability of this compound to sustain its analgesic effects with repeated administration. To test this, we used the well-established spared nerve injury (SNI) model, which induces long-lasting mechanical and cold hypersensitivity (32) (Figure 7A). As expected, SNI, but not sham-treated mice, displayed pain-like behaviors by 21 days postinjury (Figure 7, B and C). We administered C2230 at 3, 6, and 9 weeks post-SNI (Figure 7A), observing significant reductions in both mechanical (Figure 7B) and cold hypersensitivity (Figure 7C) compared with vehicle-treated SNI mice. Importantly, the analgesic effects of C2230 remained consistent over repeated doses without any signs of tolerance.

Collectively, these findings suggest that C2230 has antinociceptive effects in neuropathic pain across both sexes and provides sustained pain relief in chronic neuropathic pain models.

### C2230’s antinociceptive effects are observable along the central pain processing pathway.

**Figure 8:**
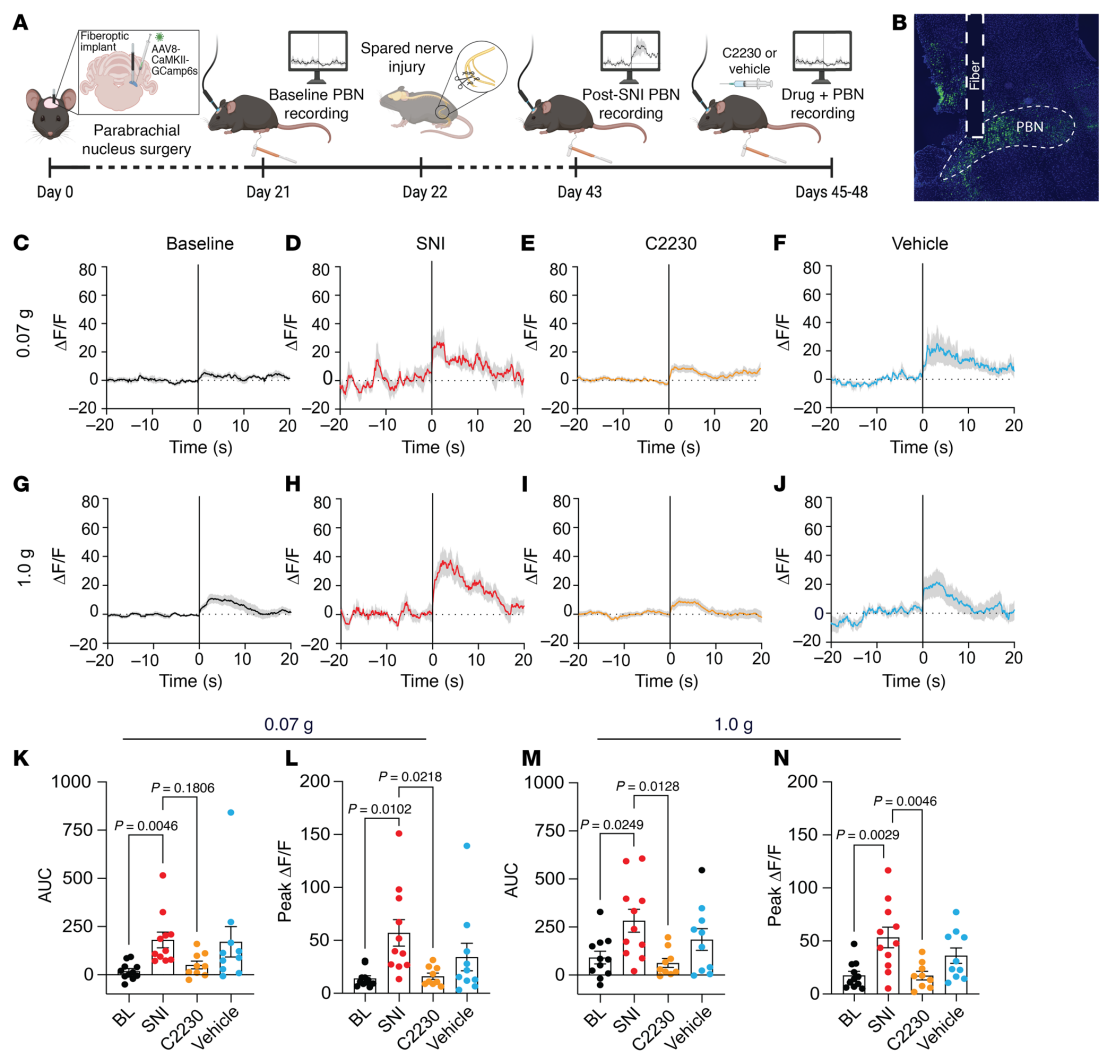
Intraperitoneal administration of C2230 (10 mg/kg) reduces spared nerve injury–induced increases in glutamatergic parabrachial nucleus activity. (**A**) Timeline schematic describing the order of events in parabrachial nucleus (PBN) recording experiments. (**B**) Representative viral expression and fiber track in the PBN. Change in the activity of GCamp6s in glutamatergic PBN neurons in response to 0.07 g or 1.0 g filament at baseline (**C** and **G**), after spared nerve injury (SNI) (**D** and **H**), 2 hours following i.p. administration of C2230 (10 mg/kg) (**E** and **I**) or vehicle (**F** and **J**). (**K**) Quantified AUC for GCamp6s activity in response to 0.07 g filament stimulation; mixed-effects analysis followed by Dunnett’s multiple comparisons test. *P* values as indicated, *n* = 9–11 mice. (**L**) Summary of peak change in fluorescence from baseline GCamp6s activity following 0.07 g stimulation; mixed-effects analysis followed by Dunnett’s multiple comparisons. *P* values as indicated, *n* = 9–11 mice. (**M**) Quantified AUC for GCamp6s activity in response to 1.0 g filament stimulation; mixed effect analysis followed by Dunnett’s multiple comparisons. *P* values as indicated, *n* = 9–11 mice. (**N**) Summary of peak change in fluorescence from baseline GCamp6s activity following 1.0 g stimulation; mixed effects analysis followed by Dunnett’s multiple comparisons. *P* values as indicated, *n* = 9–11 mice. Values are expressed as mean ± SEM. See Supplemental Table 3 for full statistical analysis.

The parabrachial nucleus (PBN) is among the first supraspinal regions to receive nociceptive input via the spinoparabrachial pathway (33), and it is a vital node in the pain processing pathway (34). Since neuropathic pain increases activity of glutamatergic neurons in the PBN (21, 35), we employed in vivo fiber photometry to monitor calcium dynamics of glutamatergic neurons during mechanical stimulation before and after induction of neuropathic pain (35), as well as 2 hours after administration of vehicle or C2230 (10 mg/kg, i.p.) (Figure 8, A and B). We found that SNI-induced neuropathic pain caused an increase in glutamatergic PBN response to nonnoxious mechanical stimuli (0.07 g or 1.0 g von Frey filaments) and the corresponding AUC when compared with baseline measurements (Figure 8, C–N). Notably, this heightened activity was reversed by i.p. administration of C2230, but not vehicle (Figure 8, C–N). These results further underscore the potential of C2230 as an effective agent for neuropathic pain relief and suggest that C2230 dampens the supraspinal relay of nociceptive transmission.

### C2230 reduces the aversion to mechanical stimuli under neuropathic pain conditions.

**Figure 9:**
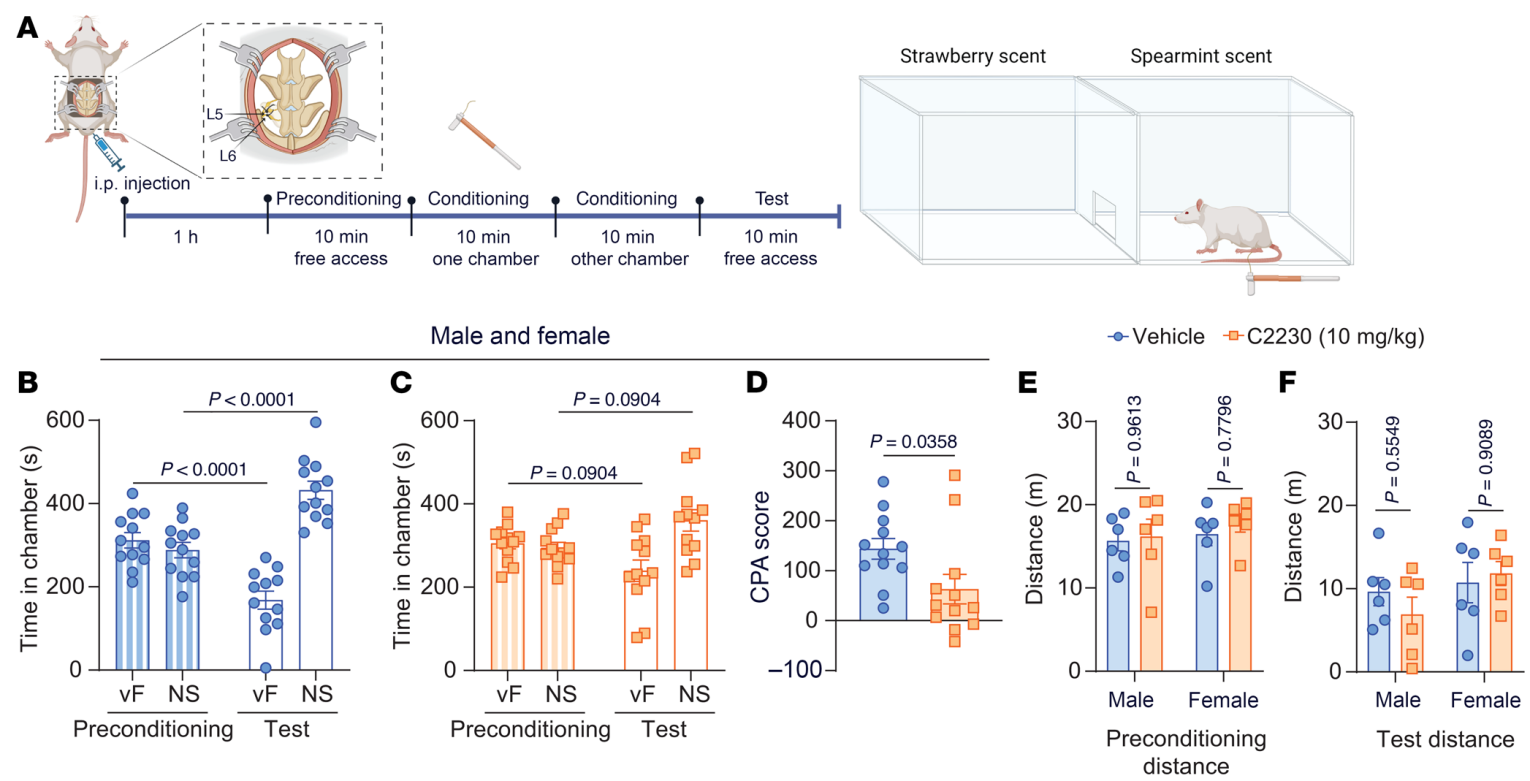
Intraperitoneal administration of C2230 (10 mg/kg) decreases aversive responses to mechanical stimulation after SNL. (**A**) Schematic timeline of the 2-chamber conditioned place aversion (CPA) test performed in SNL-injured rats. Quantification of the time spent in the vF-conditioned chamber (vF) and no stimuli (NS) chamber by vehicle-injected rats (**B**) and C2230-treated rats with SNL injury (**C**), respectively. (**D**) Quantification of CPA scores of vehicle-injected rats and C2230-treated rats with SNL injury. (**E** and **F**) Quantification of the traveled distance of vehicle-injected rats and C2230-treated rats with SNL injury during either the preconditioning or the test phase of the CPA protocol. *P* values as indicated; **B** and **C**: Bonferroni’s multiple comparison test. **D**: Unpaired *t* test; *n* = 12 rats per condition (6 male and 6 female mice); values are expressed as mean ± SEM. See Supplemental Table 3 for full statistical analysis.

We used a 2-chamber conditioned place aversion (CPA) assay to assess C2230’s effects on reducing the aversive response to mechanical stimulation in male and female neuropathic rats 14 days post-SNL (36). On the test day, animals were injected with either vehicle or C2230 (i.p.,10 mg/kg), and 2 hours later exposed to 4 × 10 minute consecutive sessions. During preconditioning, rats were allowed free access to both chambers, which were paired with a scent (strawberry or spearmint). During conditioning, rats were confined to 1 chamber, which was either paired with repeated mechanical stimulation (15 g vF filament) every 30 seconds or no stimulation (NS). In the testing phase, the rats were again allowed free access to both chambers, and aversion was assessed by measuring time spent in the chamber conditioned with mechanical stimulation (Figure 9A). Equal numbers of male and female rats were used, and, as no sex-specific effects were found, results are combined. SNL rats injected with vehicle spent equal time in both chambers during preconditioning but shifted significantly after conditioning, spending less time in the vF-conditioned chamber and more in the NS chamber (Figure 9B), indicating that mechanical stimulation with 15 g vF became aversive post-SNL. Injection with C2230 reduced the aversive effect of stimulation, as SNL rats spent similar time in the vF-conditioned chamber before and after conditioning (Figure 9C). CPA scores confirmed this, showing a significantly lower difference in C2230-treated rats (Figure 9D). This demonstrates that C2230 effectively reduces the aversive aspect to mechanical stimuli induced by the neuropathic injury. No differences in locomotor activity were observed between vehicle and C2230-treated rats (Figure 9, E and F), indicating no impact on general movement.

### Intranasal delivery of C2230 reduces orofacial pain.

**Figure 10:**
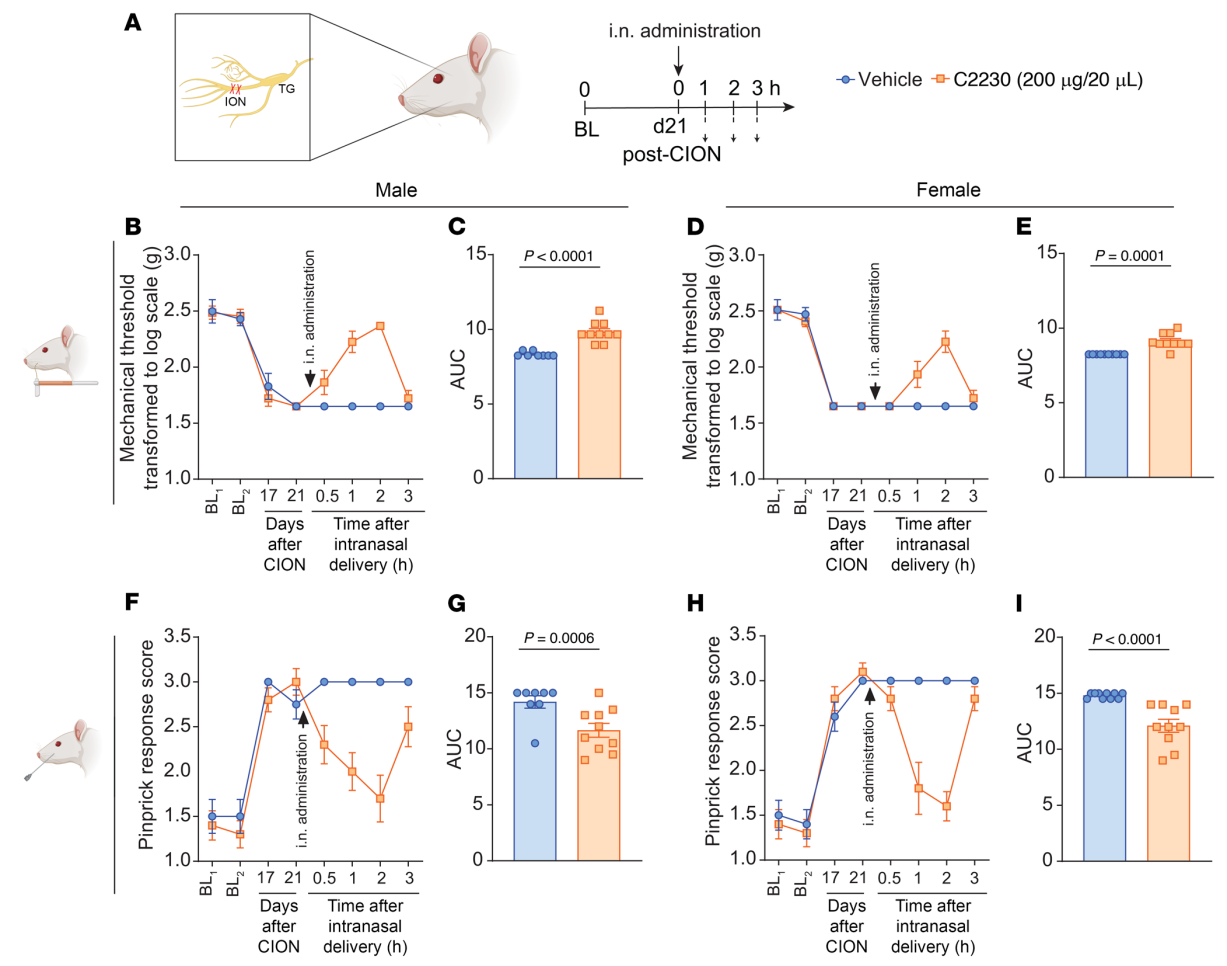
Intranasal administration of C2230 (200 μg/20 μL) effectively alleviates pain-like behaviors induced by chronic constriction of the infraorbital nerve. (**A**) Constriction of the infraorbital nerve (CION) model schematic and timeline of the experimental approach used to determine the antinociceptive effects of C2230. Time course of von Frey mechanical thresholds after i.n. administration of vehicle or C2230 in male (**B**) and female (**D**) rats; *n* = 8–10 rats per group. Quantification of the AUC of **B** and **D** between 17 days after CION and 3 hours after i.n. injection in male (**C**) and female (**E**) rats, respectively. *P* values as indicated, Mann-Whitney test; *n* = 8–10 rats per group. Time course of the pinprick response score after vehicle or C2230 i.n. administration in male (**F**) and female (**H**) rats. Quantification of the AUC of **F** and **H** between 17 days after CION and 3 hours after i.n. injection in male (**G**) and female (**I**) rats. *P* values as indicated, Mann-Whitney test; *n* = 8–10 rats per group. Values are expressed as mean ± SEM. See Supplemental Table 3 for full statistical analysis.

After observing a C2230-induced reduction in calcium currents in TG neurons similar to ω-conotoxin-GVIA (Figure 4), we tested its antinociceptive effect in a chronic constriction injury model of the infraorbital nerve (CION) in male and female rats (Figure 10A). CION led to a reduction in von Frey mechanical thresholds (Figure 10, B–E), while pinprick responses increased (Figure 10, F–I) in animals of both sexes, 17 and 21 days after injury. Intranasal administration of C2230 (200 μg/20 μL) increased the mechanical threshold and AUC in male (Figure 10, B and C) and female rats (Figure 10, D and E) compared with the vehicle-treated groups. Moreover, intranasal administration of C2230 led to a significant reduction in the pinprick response score and their respective AUC in male (Figure 10, F and G) and female rats (Figure 10, H and I). These findings demonstrate that C2230 exerts an antinociceptive effect in a model of orofacial pain.

### C2230 reverses monoiodoacetate-induced osteoarthritis-like pain.

**Figure 11:**
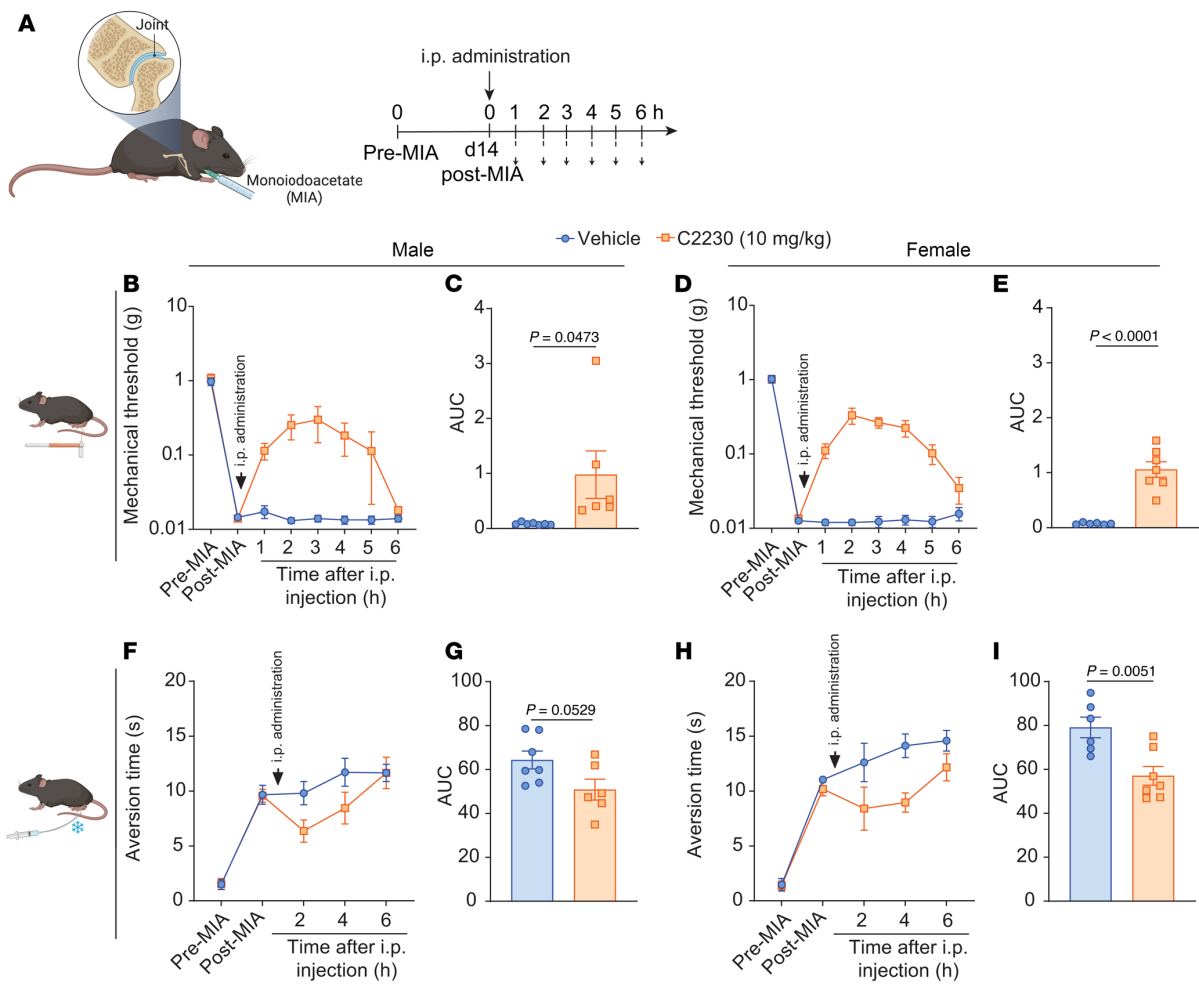
Intraperitoneal administration of C2230 (10 mg/kg) reverses monoiodoacetate-induced mechanical and cold allodynia. (**A**) Schematic depicting the monoiodoacetate (MIA) model of OA-like pain and timeline of the experimental approach used to determine the antinociceptive effects of C2230 in mice with osteoarthritis. Time course of baseline mechanical withdrawal threshold measurements conducted before (pre-MIA), after (post-MIA), and every hour after injection, for male (**B**) and female (**D**) mice. Quantification of the AUC in **B** and **D**, respectively, between post MIA to 6 hours after i.p. injection in males (**C**) and females (**E**). *P* value as indicated; unpaired *t* test. *n* = 6 male and 6 female mice per experimental group. Time course of aversion time duration to the acetone assessed before (pre-MIA), after (post-MIA), and every 2 hours after injection for male (**F**) and female mice (**H**). Quantification of the AUC in **F** and **H** respectively between post-MIA to 6 hours after i.p. injection in males (**G**) and females (**I**). *P* value as indicated; unpaired *t* test; *n* = 6 male and 6 female mice per experimental group. Values are expressed as mean ± SEM. See Supplemental Table 3 for full statistical analysis.

Osteoarthritis (OA) is a chronic progressive joint disease that causes inflammation, joint stiffness, swelling, and persistent pain (37), with Ca_V_2.2 channels implicated in OA-like pain (38). Notably, state- and use-dependent blockers of Ca_V_2.2 channels have shown potential in alleviating OA-like pain (38). To assess the potential effects of C2230 on OA-like pain, we tested the effect of C2230 (10 mg/kg, i.p.) on mechanical and cold allodynia in the Monoiodoacetate (MIA) model of OA-like pain. MIA was injected into the left knee joints of male and female mice, and, 2 weeks after injury, the animals were injected with C2230 or vehicle and assessed for mechanical and cold allodynia hourly over a 6-hour period (Figure 11A). MIA-induced mechanical sensitivity was reversed by C2230 and manifested as an increase in the AUC in both male (Figure 11, B and C) and female mice (Figure 11, D and E).

C2230 also reduced acetone-induced response duration in male (Figure 11, F and G) and female (Figure 11, H and I) mice. Analysis showed significant effects of treatment (*P* = 0.0006) and sex (*P* = 0.027), but no sex-treatment interaction (*P* = 0.35), suggesting that the treatment had significant effects across sex, but the sex difference was caused by the overall higher response duration for the females compared with males. These results offer evidence that OA-like pain can be alleviated by state- and use-dependent blockers of Ca_V_2.2.

### C2230 preserves protective pain response without impacting motor or cardiovascular functions.

**Figure 12:**
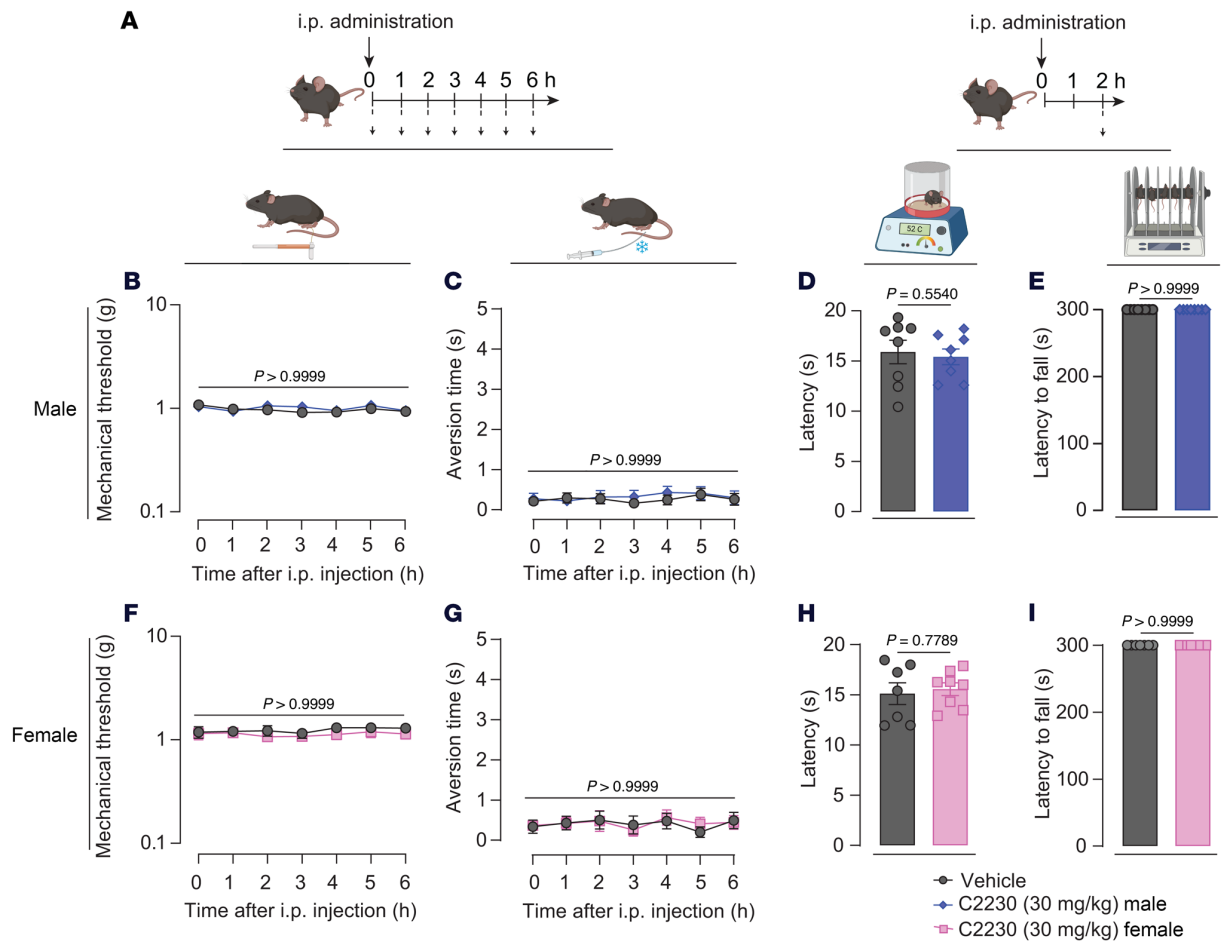
C2230 does not affect sensitivity to mechanical, cold, or nociceptive heat stimulation, nor motor function. (**A**) Left: Schematic representation and timeline of the experimental approach used to assess mechanical threshold and aversion time in naive mice following C2230 administration. Right: Schematic representation and timeline of the experimental approach used to evaluate thermal stimulation responses and motor function in naive mice 2 hours after C2230 administration. (**B** and **F**) Time course of von Frey mechanical thresholds after i.p. administration of vehicle or C2230 in naive male and female mice. (**C** and **G**) Time course of aversion time responses to an acetone drop following i.p. administration of vehicle or C2230 in naive male and female mice. *P* values as indicated; 2-way ANOVA followed by Bonferroni’s multiple comparison test; *n* = 8 mice per group. (**D** and **H**) Bar graphs showing withdrawal latency to a 52°C nociceptive stimulus in male and female naive mice 2 hours after C2230 injection. (**E** and **I**) Bar graphs representing latency to fall in the rotarod test for male and female naive mice 2 hours after C2230 injection. *P* values as indicated, Mann Whitney test; *n* = 8 mice per group. Values are expressed as mean ± SEM. See Supplemental Table 3 for full statistical analysis.

To ensure that C2230 does not interfere with the protective function of pain, we evaluated the effect of the highest dose (30 mg/kg) on mechanical and thermal stimuli response in naive male and female mice 2 hours after i.p. injection (Figure 12A). In males, C2230 did not affect mechanical (Figure 12B) or cold sensitivity (Figure 12C) or withdrawal latency to a 52°C nociceptive stimulus (Figure 12D). Since TROX-1 affected motor and cardiovascular functions (17), we tested if C2230 induced similar side effects. Motor function was unchanged in naive mice 2 hours after i.p. administration of C2230 (30 mg/kg), as assessed by the rotarod test (Figure 12E). Similar findings were observed in female mice (Figure 12, F–I). Next, the effect of C2230 on terminal mean arterial blood pressure (MABP) and mean heart rate (MHR) were recorded using a radio telemetry catheter 2 hours after i.p. administration of C2230 (10 mg/kg). C2230 treatment had no significant effect on either MABP or MHR (Supplemental Figure 7) of naive male mice. Together, these findings demonstrate that C2230 does not affect somatosensation or compromise the protective pain response, nor does it impair motor or cardiovascular function.

### C2230 does not bind to the DIII/DIV fenestration of the rat Ca_V_2.2 channel.

**Figure 13:**
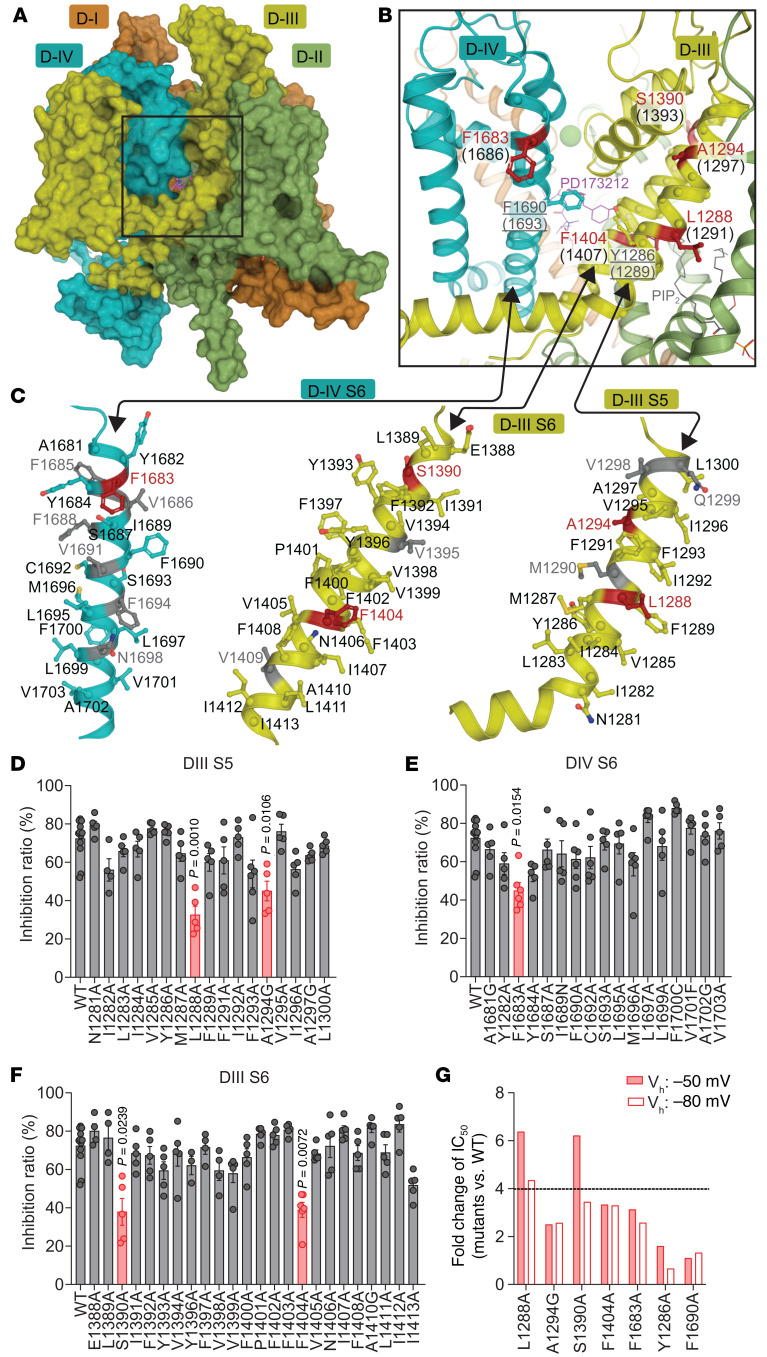
Locations of alanine-scanning mutation sites on Ca_V_ 2.2. (**A**) Surface representation of the human Ca_V_2.2 α subunit (PDB: 7VFV(40)) with bound PD173212 in stick representation. View facing the open D-III/D-IV fenestration. (**B**) Ribbon representation through the D-III/D-IV fenestration with the alanine mutation sites on the D-III S5, S6 and D-IV S6 helices shown as spheres. The mutations affecting inhibition by C2230 are shown as sticks and labeled according to their rat Ca_V_2.2 sequence, with the corresponding human numbering in parentheses. Residues important for PD173212 binding (40) also shown as sticks and their labels are underlined. (**C**) Close up views showing all amino acids with their rat sequence numbers. (**D**–**F**) Bar graphs of percent inhibition by 20 μM C2230 for alanine scan of D-III S5 (**D**), D-IV S6 (**E**), and D-III S6 (**F**) helices. Data in red bars indicate mutations affecting inhibition by C2230 versus WT while gray bars denote mutations that were not different from WT. Mutations of L1288A, A1294G, S1390A, F1404A, and F1683A significantly reduced inhibition C2230 when compared with the WT. Channels were clamped at –80 mV and currents were elicited by depolarization to + 10 mV. *P* values are as indicated, Kruskal-Wallis test followed by Dunn’s post hoc test; *n* = 4–12 cells from 2–3 independent experiments. (**G**) The fold change in IC_50_ values for C2230 inhibiting the specified mutants is presented relative to its inhibition of the WT Ca_V_ 2.2 channel, assessed at holding potentials of –80 mV and –50 mV. Note the IC_50_s of C2230 on these mutants at –80 mV holding potential were calculated using the Hill equation (IC_50_ = [C2230] × I_res_/(1 – I_res_)), where I_res_ for each mutant equals to ‘1 – inhibition ratio’ as determined in **D**–**F**. The IC_50_s of C2230 on these mutants at –50 mV holding, however, were experimentally determined (*n* = 5–6). See Supplemental Table 3 for full statistical analysis.

Recent structural analyses (39, 40) have yielded remarkable insights into the mechanisms of action of voltage-gated calcium channel modulators. For example, dihydropyridines have been found to allosterically inhibit the activity of L-type Ca^2+^ channels by binding to their DIII–DIV fenestration (41, 42). Similarly, benzothiazepines and phenylalkylamines directly block ion influx by occupying the central cavity of the channel pore (41). Based on the observed binding location of 2 Ca_V_2.2 antagonists, PD173212 and “Ca_V_2.2 blocker 1”, in the pore and the DIII/DIV fenestration (40) (Figure 13, A and B), we targeted the rat Ca_V_2.2 DIII S5, S6 and DIV S6 helices (Figure 13C) for alanine scanning to determine if these binding sites are also utilized by C2230. Most of these mutant channels were functionally expressed when transfected in HEK293T cells. Their response to current inhibition by 20 μM C2230 at a V_h_ of –80 mV was measured and compared with that of WT Ca_V_2.2 (Figure 13, D and F). The L1288A and A1294G mutations in DIII S5 helix, the F1683A mutation in DIV S6 helix, and the S1390A and F1404A mutations in DIII S6 helix, had reduced inhibition of Ca_V_2.2 currents by C2230 (Figure 13, D–F). As depicted in Figures 13, B and C, these amino acids point away from the pore and the DIII/DIV fenestration. Thus, the only mutations that affected C2230 are not in the fenestration opening and they do not form part of the pore (where PD173212 and “blocker 1” bind). F1683 points out toward the membrane, A1294 and S1390 point toward the P1 helices, F1404 contacts DIII-S5, and L1288 contacts the D-II voltage-sensing helix S4 (Figure 13B). Using the Hill equation to calculate the IC_50_ values of C2230 against these mutant channels, we found a 2- to 4-fold difference compared with the WT Ca_V_2.2 channel (Figure 13G). These findings suggest that these amino acids are not the key residues involved in binding C2230, although they might allosterically contribute to the formation of a binding pocket for C2230.

As C2230 preferably binds to the inactivated Ca_V_2.2 channels, we asked if these residues were important for the inactivated but not the resting state binding of the compound. When we conducted experiments at a V_h_ of –50 mV, mutations A1294G, F1404A, and F1683A showed similar increases in IC_50_ values as were observed at –80 mV (Figure 13G and Supplemental Figure 8A). For the L1288A and S1390A mutations, there was an approximate 6-fold change in IC_50_, slightly higher than that observed at –80 mV (Figure 13G and Supplemental Figure 8A). Mutations of the other 2 residues, Y1286 in DIII S5, and F1690 in DIV S6, which were critical for binding with PD173212 and “Ca_V_2.2 blocker 1” (Figure 13B), did not change the potency of C2230 at both holding potentials (Figure 13G and Supplemental Figure 8B). These findings collectively suggest that these residues are not critical for accommodating C2230 in the resting and inactivated state channels. C2230 likely binds to the central cavity or possibly other fenestration sites aside from the DIII/DIV fenestration in the Ca_V_2.2 channel.

## Discussion

The severe side effects of pain medications present a considerable hurdle for effective pain management (43). Opioids — a cornerstone of pain management — induce unpleasant symptoms such as nausea, constipation, addiction, tolerance, sedation, and the potential for substance use disorder (43). Consequently, the pursuit of alternative nonopioid treatments, such as Ca_V_2.2 channel blockers/modulators, offers a promising avenue to diminish the dependence on opioids for pain relief and contribute to the battle against the national opioid crisis.

Approved by the FDA in 2004, Ziconotide became the first nonopioid intrathecal analgesic for the treatment of severe chronic pain in patients unresponsive to opioid therapy (11). In contrast to opioid-based treatments, Ziconotide does not give rise to tolerance (9, 44, 45), however, it produces severe side effects, including dizziness, nystagmus, somnolence, abnormal gait, and ataxia, which limit its wider application (46, 47). Snail-derived ω-conotoxins specific to Ca_V_2.2 channels have emerged as promising analgesic candidates. CVID (also known as Leconotide), obtained from *Conus catus*, is a selective peptide antagonist of Ca_V_2.2. that advanced into clinical trials (48) and performed better than Ziconotide, due to having less toxicity and the ability to be administered intravenously (49), as opposed to the intrathecal injection of Ziconotide. Additionally, the GVIA ω-conotoxin, derived from *Conus geographus*, exhibits an impressive ability to permanently block Ca_V_2.2 channels at nanomolar concentrations (50), and it demonstrates higher in vivo potency when compared with its structurally similar counterparts (9, 27, 51).

Studies to elucidate the toxicity of Ca_V_2.2 calcium channel inhibitors, such as conotoxins, have been essential in mapping the underlying causes of the side effects associated with these drugs. One research team that identified ω-conotoxin SO-3 (isolated from *Conus striatus*), which shared structural similarities and analgesic properties with MVIIA — the cone snail peptide ω-conotoxin that formed the basis of Ziconotide (52–54) — found that substitution of MVIIA’s loop 2 with the loop 2 of SO-3 not only enhanced the binding of the peptide to Ca_V_2.2 but also decreased its toxicity. This led to the amelioration of side effects such as tremors, spontaneous locomotor activity, and uncoordinated locomotion function (54). Subsequent work identified Met12 in the loop 2 region as the primary source of MVIIA’s toxicity. Understanding the origins of MVIIA’s toxicity holds significant implications for the development of safer and more efficacious Ca_V_2.2 calcium channel inhibitors (54).

Although Ca_V_2.2 pore blockers (55) have demonstrated analgesic efficacy, recent progress has shifted toward the discovery of state- and/or use- dependent Ca_V_2.2 inhibitors. These compounds exhibit a preference for targeting channels in their open and/or inactivated states and/or when they are frequently or continuously activated. The Ca_V_2.2 inhibitor “T4,” for example, has strong state dependence and preferentially interacts with inactivated Ca_V_2.2 channels, produces only weak use-dependent inhibition, and displays very fast recovery from inactivated-state inhibition at hyperpolarized potentials (56). Likewise, the compound ZC88 acts as a state-dependent inhibitor of transiently expressed Ca_V_2.2 channels by blocking their inactivated state in oocytes (57). Notably, earlier research also indicated that ZC88 targeted hERG potassium channels (58), which hampered its progress in further development. Although the antinociceptive properties of T4 and ZC88 were not explored, other use- and state-dependent Ca_V_2.2 inhibitors have exhibited efficacy in preclinical pain models (18).

In our study, we found that C2230 exhibits dual attributes as a state-dependent and use-dependent Ca_V_2.2 channel blocker. C2230 preferably inhibits Ca_V_2.2 channels at –50 mV, compared with other voltage-gated ion channels. Additionally, it stabilizes the inactivated state of Ca_V_2.2 channels, accelerates the OSI of the channel, and inhibits the channel during high-frequency stimulation. Blocking Ca_V_2.2 channels during heightened neuronal firing, as in pain states, offers an advantage over other blockers. C2230 may have minimal impact on normal physiological functions while showing promising drug likeness (Supplemental Table 1).

It is essential to recognize that certain compounds can elicit species-specific effects. For example, the Na_V_1.7 channel blocker Protoxin-II inhibits rat Na_V_1.7 currents but is not effective, nor selective, against Na_V_1.7 channels in human DRG neurons (25). Our observations of reduced calcium currents by C2230 in rat DRG neurons mirrored what we detected in human sensory neurons. Furthermore, the data we collected from rat TGs matched the marmoset TGs, and together provide support for our conclusions that C2230 is an effective Ca_V_2.2 blocker across species and neuronal populations. Of significant note is that the impact of C2230 on total calcium currents in TG neurons paralleled the effects observed with ω-conotoxin-GVIA. One distinctive feature of ω-conotoxin-GVIA is its slow onset and recovery kinetics (59, 60), rendering it an almost irreversible inhibitor of the Ca_V_2.2 channels (61), complicating dose control in a clinical setting. Additionally, like Ziconotide, ω-conotoxin-GVIA requires intrathecal administration to be effective therapeutically (9). C2230 effectively relieved pain via 3 routes of administration without side effects and maintained its efficacy with repeated use. This is crucial, as many analgesics, like opioids, lose effectiveness over time due to tolerance, limiting their long-term use for chronic pain (62, 63).

TROX-1, a small molecule state- and use-dependent inhibitor of Ca_V_2.2 (18), induced antinociceptive effects in preclinical models of pain, including a model of OA-like pain (38), but side effects related to cardiovascular and motor impairment impeded its further development (17). Despite a high likelihood of CNS exposure (Supplemental Table 1) and the compound’s ability to cross the blood-brain barrier — resulting in higher systemic exposure in the brain compared with plasma (Supplemental Figure 9) — C2230 showed no signs of impairing locomotor activity or normal exploratory behavior, indicating no evidence of toxicity following a single systemic injection. The lack of effect of C2230 on locomotor activity, heart rate, and blood pressure at concentrations that produced analgesia in a wide range of rodent pain models likely results from state-dependent block of Ca_V_2.2 in a manner that possesses minimal activity against non–Ca_V_2.2 ion channels and other nonspecific molecular and physiological targets (64). In this context, although C2230 also blocks Ca_V_1.2 and Ca_V_1.3 by approximately 50% and 30%, respectively, the IC_50_s of inhibition of these channels are 15-fold (Ca_V_1.2) to 38-fold (Ca_V_1.3) higher than that of Ca_V_2.2, reducing the chance of side effects due to actions on these channels. Moreover, C2230 exhibited only a weak inhibition of the skeletal muscle Na_V_1.4 channel and the cardiac K_V_4.2–4.3 channels (Supplemental Figure 2).

Aversion associated with pain and injury is thought to be mediated by limbic areas in the brain. We found that systemic administration of C2230 decreased nerve injury–induced increases in glutamatergic response in the PBN to nonnoxious mechanical stimuli in mice. Similarly, we found that C2230 decreased mechanical aversion in a CPA assay in rats. We hypothesize that C2230-induced reduction in excitatory neurotransmitter release from DRG neurons into the spinal cord dorsal horn reduced the ascending transmission of nociceptive information from the spinal cord to the brain. However, C2230 may be reducing aversion via direct CNS effects in limbic brain regions. For example, microinjection of ω-conotoxin-GVIA into the central nucleus of the amygdala decreased conditioned aversion responses in an inflammatory pain model (65), and local microinjection of MVIIA in the rostral ventromedial medulla reduced nociceptive behaviors induced by nerve injury (66).

Achieving selectivity in ion channel targeting can be challenging, primarily due to the structural similarities shared among various voltage-gated ion channel isoforms. For instance, roscovitine, an inhibitor of cyclin-dependent kinases (67), slows deactivation of all Ca_V_2 channels (N, P/Q and R) by binding to their open state (68, 69). On the other hand, it is worth noting that targeting multiple ion channel isoforms simultaneously can also offer many advantages. For instance, A-1048400, an orally active state-dependent neuronal calcium channel blocker, has demonstrated antinociceptive properties by targeting recombinant human and native rat N-, P/Q-, and T-type calcium channels (70). Similarly, CNCB-2, a dual ion channel inhibitor, employs a state-dependent approach to inhibit Ca_V_2.2 channels while also inhibiting Na_V_1.7 channels in a state- and use-dependent manner, resulting in long-lasting analgesic effects in models of inflammatory and neuropathic pain (71). It has been suggested that calcium channel blockers that exhibit a preference for binding to and stabilizing the inactivated states of the channel (70) could potentially yield potent pain relief without the adverse effects on cardiovascular or central nervous system functions commonly associated with voltage-independent peptide blockers like Ziconotide (4, 72).

Pharmacokinetic (PK) analysis showed that C2230 (i.p., 10 mg/kg) reached peak brain and plasma concentrations within 30 minutes (T_max_), with mean maximum concentrations of 960.2 ± 180.9 ng/mL in the brain and 158.8 ± 28.0 ng/mL in plasma (C_max_). The elimination half-life was 1.1 hours in the brain and 1.2 hours in plasma (Supplemental Figure 9). These biological findings provide a foundation for future medicinal chemistry efforts to enhance the efficacy and drug-like properties of C2230. The current studies evaluated a racemic mixture of 1-(2-(tert-butyl)-4-methoxyphenoxy)-3-(cyclopentylamino)propan-2-ol. C2230 features several rotatable bonds providing an opportunity to explore aliphatic rings to constraint geometry. Whether C2230 will face the same challenges as Z160 remains uncertain. However, in our hands, C2230 has shown robust efficacy and favorable PK properties in preclinical models, suggesting its potential to overcome the limitations encountered by previous compounds in its class. In ongoing studies, we are determining if a stereochemical pure derivative provides an improvement in selectivity and analgesic potency.

In summary, C2230 relieves pain in neuropathic, orofacial, and osteoarthritis models, underscoring the potential of use- and state-dependent Ca_V_2.2 blockers for managing diverse pain conditions.

## Methods

### Sex as a biological variable

In pain behavioral testing, sex was considered a biological variable, and both male and female mice were included. Since no sex differences were observed, subsequent analyses were conducted as follows: for electrophysiology and calcitonin gene-related peptide (CGRP) release analyses, spinal cord and DRG neurons were obtained from female Sprague-Dawley rats, TG neurons were collected from female Sprague-Dawley rats and a male marmoset. Spinal cord slices were prepared from male Sprague-Dawley rat pups. For pharmacokinetic study and hemodynamic measurements, male mice were used exclusively. We believe that the findings from these experiments, are relevant to both male and female sexes.

Full methods are available in Supplemental materials. All procedures involving electrophysiology, biochemistry, and behavior adhered to established protocols (73, 74).

### Data Availability

All data are available in the main text, figures, supplemental materials, and in the Supporting Data Values file.

### Statistics

Data are presented as mean ± SEM. Statistical analyses were performed using Prism 9 (GraphPad Software). Full statistical details are provided in Supplemental Table 3. In brief, a 2-tailed unpaired Student’s *t* test was used to compare 2 normally distributed sample groups, with equality of variances tested using the F test. A paired *t* test was performed for data from experiments with a paired design. For comparisons involving more than 2 groups, a 1-way ANOVA was conducted, followed by Tukey’s or Dunnett’s multiple comparisons tests, with equality of variances assessed using the Brown-Forsythe and Bartlett’s tests. The Mann-Whitney test and Kruskal-Wallis test were used for comparing 2 or more nonnormally distributed sample groups, respectively. A *P* value less-than 0.05 was considered statistically significant. The *n* value represents the number of independent biological replicates. No statistical method was used to predetermine the sample size (*n* value); however, we adopted sample sizes in the same range as those previously reported in the literature for similar experiments. The experiments were randomized, and investigators were blinded to allocations during experiments and outcome assessment.

### Study approval

#### Rodents.

All animal use was conducted in accordance with the National Institutes of Health guidelines, and the study was approved and conducted in strict accordance with recommendations in the Guide for the Care and Use of Laboratory Animals of the College of Dentistry at New York University, The University of Florida, and Rutgers School of Dental Medicine. All efforts were made to minimize animal suffering.

#### Nonhuman primate tissue.

All animal experiments conformed to IASP’s Guiding Principles in the Care and Use of Vertebrate Animals in Research and Training. We also followed guidelines issued by the National Institutes of Health (NIH) and the Society for Neuroscience (SfN) to minimize the number of animals used and their suffering. All animal experiments conformed to protocols approved by the UTHSCSA and Texas Biomedical Research Institute Institutional Animal Care and Use Committee.

#### Human tissue.

All human tissue procurement procedures were approved by the Institutional Review Boards at University of Texas at Dallas, New York University, and the University of Florida.

## Author contributions

CT and RK developed the concept of this project, designed, and supervised experiments and advised in data analysis. CT, KG, AP, and RK wrote the manuscript. CT, KG, YC, HNA, EJRP, SH, SLL, ACR, PD, TSN, SRRK, BS, RKS, and JY conducted experiments and acquired data. NRP synthesized the compound. SPM performed docking studies. SR provided marmoset tissue. MKS and TJP provided human DRG cells. PSA, OAK, and AS designed and supervised experiments performed in their laboratories. All authors had the opportunity to discuss the results and comment on the manuscript. The equal contribution of the first 3 authors was determined by proximity to decisions regarding tenure, graduation, and early career transitions.

## Supplementary Material

Supplemental data

Supporting data values

## Version 1 12/10/2024

In-Press Preview

## Version 2 02/17/2025

Electronic publication
