# Supplementary material for: C2230, a preferential use- and state-dependent CaV2.2 channel blocker, mitigates pain behaviors across multiple pain models
Source: J Clin Invest. 2024 Dec 10;135(4):e177429. doi: 10.1172/JCI177429 (PMC11827853; doi:10.1172/JCI177429)
Supplement: Supplemental data [file jci-135-177429-s066.pdf]

C2230, a preferential use- and state-dependent $Ca_V2.2$ channel blocker, mitigates pain behaviors across multiple pain models

Cheng Tang[1,2,3,#], Kimberly Gomez[4,#], Yan Chen[3,#], Heather N. Allen[4], Sara Hestehave[1,2], Erick J. Rodríguez-Palma[4], Santiago Loya-López[4], Aida Calderon-Rivera[4], Paz Duran[1,2], Tyler S. Nelson[4], Siva Rama Raju Kanumuri[5], Bijal Shah[6], Nihar R. Panigrahi[7], Samantha Perez-Miller[4], Morgan Schackmuth[8], Shivani Ruparel[9], Amol Patwardhan[10], Theodore J. Price[8], Paramjit S. Arora[7], Ravindra K. Sharma[11], Abhisheak Sharma[5], Jie Yu[12], Olga A. Korczeniewska[6], Rajesh Khanna[4]*

[1] Department of Molecular Pathobiology, College of Dentistry, New York University, New York, New York 10010, USA

[2] Pain Research Center, New York University, New York, NY 10010, USA

[3]The National and Local Joint Engineering Laboratory of Animal Peptide Drug Development, College of Life Sciences, Hunan Normal University, Changsha 410081, China

[4] Department of Pharmacology and Therapeutics, College of Medicine, University of Florida, Gainesville, FL 32610, USA

[5] Department of Pharmaceutics, College of Pharmacy, University of Florida, Gainesville, FL 32610, USA

[6] Center for Orofacial Pain and Temporomandibular Disorders, Department of Diagnostic Sciences, Rutgers School of Dental Medicine, Newark, NJ 07101, USA

[7] Department of Chemistry, New York University, New York, NY 10003, USA

[8] University of Texas at Dallas, School of Behavioral and Brain Sciences, Department of Neuroscience, Center for Advanced Pain Studies, Richardson TX, 75080, USA

[9] Department of Endodontics, School of Dentistry, University of Texas Health Science Center at San Antonio, and Center for Pain Therapeutics and Addiction Research, San Antonio, TX, 78229, USA

[10] Department of Anesthesiology and Pain Management, University of Texas Southwestern Medical Center, Dallas, TX, USA

[11] Department of Physiology and Aging, College of Medicine, University of Florida, Gainesville, FL 32610, USA

[12] Institute of Chinese Medicine Clinical Basics, Institute of Chinese Medicine on Rheumatology, College of Basic Medical Science, Zhejiang Chinese Medical University, Hangzhou 310053, China

[#]Contributed equally to this work

*To whom correspondence should be addressed: Dr. Rajesh Khanna, McKnight Brain Institute,1149 Newell Dr, Room L4-183. Gainesville, FL 32610. Phone number: (520) 271-0433. Email: r.khanna@ufl.edu

This PDF file includes:

Supplemental Methods

Supplemental Figures 1 to 9

Supplemental Table 1 to 3

Supplemental References

Supplemental methods

Synthesis of 1-(2-(tert-butyl)-4-methoxyphenoxy)-3-(cyclopentylamino)propan-2-ol

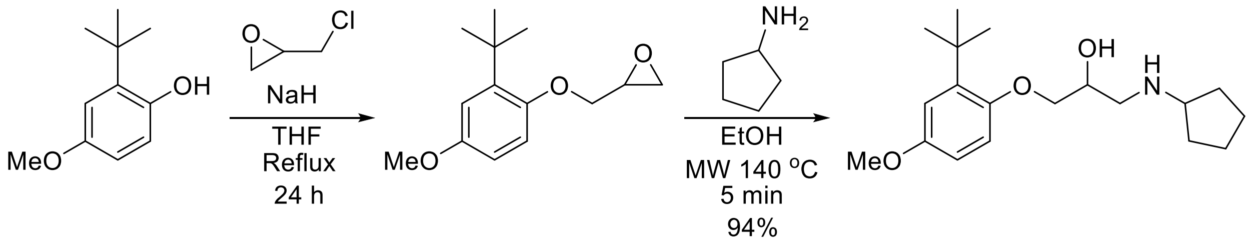

Step 1. 2-((2-(tert-butyl)-4-methoxyphenoxy)methyl)oxirane

NaH (60% suspension in oil, 0.333 mg, 8.32 mmol) was added slowly to a solution of 2-(tert-butyl)-4-methoxyphenol (1.00 g, 5.55 mmol) in anhydrous THF (10.0 mL). Evolution of gas was observed, and the color of the solution turned to turquoise blue. After stirring for 30 minutes at room temperature, epichlorohydrin (1.30 mL, 16.6 mmol) was added to the solution, and the mixture refluxed. After 24 h, the mixture was concentrated on a rotary evaporator. The residue was partitioned between Et_2O and 5% aqueous citric acid solution. The ether layer was collected, washed with water and brine, and dried over anhydrous Na_2SO_4. Rotary evaporation of Et_2O afforded crude 2-((2-(tert-butyl)-4-methoxyphenoxy)methyl)oxirane (1.31 g, quantitative yield) as pale yellow oil, which was used in the next step without further purification. ^1H NMR (600 MHz, $CDCl_3$) δ 6.90 (d, J = 2.8 Hz, 1H), 6.78 (d, J = 8.8 Hz, 1H), 6.67 (dd, J = 8.8, 2.8 Hz, 1H), 4.21 (dd, J = 10.8, 2.9 Hz, 1H), 3.96 (dd, J = 10.8, 5.5 Hz, 1H), 3.77 (s, 3H), 3.42 – 3.36 (m, 1H), 2.94 – 2.90 (m, 1H), 2.78 (dd, J = 4.8, 2.6 Hz, 1H), 1.40 (s, 9H). ^{13}C NMR (151 MHz, $CDCl_3$) δ 153.7, 151.7, 140.2, 114.5, 113.3, 109.9, 69.5, 55.7, 50.5, 44.9, 35.1, 29.9. HRMS calcd for $C_{14}H_{20}O_3Na$ $[M+Na]^+$: 259.1341; found: 259.1305.

Step 2. 1-(2-(tert-butyl)-4-methoxyphenoxy)-3-(cyclopentylamino)propan-2-ol

A mixture of 2-((2-(tert-butyl)-4-methoxyphenoxy)methyl)oxirane (200 mg, 0.899 mmol) and cyclopentylamine (115 mg, 133 µL, 1.35 mmol) in EtOH (2.2 mL) was heated to 140°C using microwave irradiation for 5 minutes. The solution was allowed to cool to room temperature and the solvent was evaporated under reduced pressure. The resulting residue was then purified by flash column chromatography (gradient elution of 0% to 10% MeOH in CH_2Cl_2) to afford 1-(2-(tert-butyl)-4-

methoxyphenoxy)-3-(cyclopentylamino)propan-2-ol as white solid (273 mg, 94% yield). ^{1}H NMR (600 MHz, $CDCl_3$) δ 6.88 (d, J = 3.1 Hz, 1H), 6.79 (d, J = 8.8 Hz, 1H), 6.66 (dd, J = 8.8, 3.1 Hz, 1H), 4.75 (br, 2H), 4.34 – 4.23 (m, 1H), 4.02 (dd, J = 9.5, 5.1 Hz, 1H), 3.94 (dd, J = 9.5, 5.5 Hz, 1H), 3.76 (s, 3H), 3.30 – 3.24 (m, 1H), 3.11 (dd, J = 12.3, 3.2 Hz, 1H), 2.93 (dd, J = 12.3, 8.9 Hz, 1H), 2.02 – 1.88 (m, 2H), 1.84 – 1.70 (m, 2H), 1.65 – 1.49 (m, 4H), 1.37 (s, 9H). ^{13}C NMR (151 MHz, $CDCl_3$) δ 153.6, 151.6, 139.80, 114.6, 113.0, 109.9, 70.8, 67.8, 60.2, 55.7, 51.1, 35.0, 32.1, 32.0, 30.0, 24.1, 24.0. HRMS calcd for $C_{19}H_{32}NO_3[M+H]^+$: 322.2353; found: 322.2377.

Screening of compound library

A chemical compound library containing 4,208 compounds featuring different core structures and structural diversity was purchased from Selleck (Selleck Chemicals LLC, Catalog No. L3600). Using manual whole-cell patch-clamp recording, we tested all 4208 compounds for their ability to inhibit heterologously expressed $Ca_V2.2$ currents at a concentration of 10 μM. A stable recording of $Ca_V2.2$ currents was initially established in a positively transfected HEK cell. Next, 0.1% DMSO (in bath) was applied by perfusion as a negative control, since the library compounds were dissolved in a bath solution containing 0.1% DMSO, to confirm that the solvent did not influence the currents. Subsequently, the different compounds were sequentially applied. Most compounds showed no activity, and no more than 10 compounds were added before the recording cell was discarded. After each recording, ω-Ctx GVIA was added as a positive control to validate the currents were $Ca_V2.2$. Each compound was applied to three different recording cells to conduct three independent replications. During the screening process, all compounds exhibiting fast and apparent inhibition of the currents were documented but only those compounds exhibiting >50% inhibition of the currents were identified as positive ‘hits’. This screening campaign resulted in the identification of one compound, 1-(2-(tert-butyl)-4-methoxyphenoxy)-3-(cyclopentylamino)propan-2-ol (designated as C2230), as a potent inhibitor of the $Ca_V2.2$ channel and two less potent $Ca_V2.2$ antagonists, C1740 and C0854 (**Supplemental Figure 1**). C2230 was then synthesized (as reported in the above section), purified to homogeneity, and structurally validated by ^{1}H-NMR and ^{13}C-NMR analysis.

Animals

All experiments and procedures were performed in accordance with the guidelines recommended by the National Institutes of Health, the International Association for the Study of Pain, and the National Centre for the Replacement, Refinement, and Reduction of Animals in Research (NC3Rs) guidelines. No animals showed any signs of adverse effects from the administration of C2230.

Rats: Animals were housed in the New York University Kriser Dental Center Animal Facility in light (12-h light: 12-h dark cycle; lights on at 07:00 h) and temperature (23 ± 3°C) controlled rooms. Adult female Sprague-Dawley rats (~200 g, Charles River Laboratories, Wilmington, MA) were used for calcitonin gene-related peptide (CGRP) release assay. Female Sprague-Dawley rats (~75-100 g, Charles River Laboratories, Wilmington, MA) were employed for electrophysiological recordings. Adult male Sprague-Dawley rats (~100 g, Charles River Laboratories, Wilmington, MA) were used to establish a spinal nerve ligation (SNL) pain model. For the conditioned place aversion (CPA) paradigm, both male and female rats were included. Adult male and female Sprague-Dawley rats (56 days old, Charles River Laboratories, Wilmington, MA) were used to create a chronic constriction injury of the infraorbital nerve (CION) pain model.

Mice: C57BL/6 mice were housed in the New York University Kriser Dental Center Animal Facility and in the University of Florida MBI animal facility. Animals were kept in light (12-h light: 12-h dark cycle; lights on at 07:00 h) and temperature (23 ± 3°C) controlled rooms. Adult male and female C57BL/6J mice (8–10 weeks of age, The Jackson Laboratory, JAX - Bar Harbor, ME) were used for creating pain models of SNL, spared nerve injury (SNI), and monoiodoacetate-induced osteoarthritis-like pain (MIA).

Marmosets: Animals were housed at the University of Texas Health Science Center at San Antonio (UTHSCSA) and Texas Biomedical Research Institute (TBRI, San Antonio, TX). Samples for this study were collected opportunistically, through the "Tissue Share" program in UTHSCSA and TBRI, from animals that were euthanized at IACUC– or TBRI–approved endpoints on their respective studies. Marmosets used in this study did not have injury affecting the head or neck area, nor systemic infections. For our study, we collected tissue from one 5-year-old male marmoset for all experiments.

Biochemistry

CGRP release assay: Adult female Sprague-Dawley rats were anesthetized with 5% isofluorane and then decapitated. Two vertebral incisions (cervical and lumbar) were made to expose the spinal cord. Pressure was applied to a saline-filled syringe inserted into the lumbar vertebral foramen, and the spinal cord was extracted. Only the lumbar region of the spinal cord was used for the calcitonin gene-related peptide (CGRP) release assay. Baseline treatments involved bathing the spinal cord in standard Tyrode solution. The excitatory solution, consisting of 90 mM KCl, was paired with the treatment. These fractions (5 minutes, 700 μL each) were collected for measurement of CGRP release. Samples were immediately stored in a −20˚C freezer. C2230 (20 μM), or vehicle (0.1% DMSO) was added to the pretreatment and cotreatment fractions. The concentration of CGRP released into the buffer was measured by enzyme-linked immunosorbent assay (Cat# 589001; Cayman Chemical, Ann Arbor, MI).

In vivo calcium imaging (fiber photometry): Adult male and female C57BL/6J mice received 500 nL of AAV9-CaMKIIa-GCamp6s-WPRE-SV40 (Addgene, Watertown, MA) in the right parabrachial nucleus (PBN) to transfect glutamatergic PBN neurons with the calcium indicator GCamp6s (coordinates: A/P-5.15 mm, M/L+/- 1.45 mm, D/V-3.45 mm). Virus was precisely administered with a Nanoject II Auto-Nanoliter Injector (Drummond) at a rate of 2 nL/sec and a wait time of 5 minutes to prevent backflow. Directly following viral infusion, a fiber optic cannula with black ceramic ferrule (RWD, 1.25 mm ferrule diameter, 200 μm core diameter, and 0.37 numerical aperture) was chronically implanted in the right PBN and fixed to the skull using dental cement (Cat# 10-000-786, Stoelting, Wood Dale, Il). Mice were allowed 21 days to recover before undergoing baseline testing. Mice were acclimated in acrylic boxes on wire mesh with fiber optic patch cord attached for at least one hour prior to testing. Calcium transients were collected continuously (FP3002, Neurophotometrics) during the mechanical stimulation protocol. A 0.07 g and 1.0 g von Frey filament were applied perpendicularly to the outer plantar surface of the left hindpaw for approximately one second. The stimulus was delivered three times, with a two minute interval between each application, and all three responses were averaged to represent the animal's response. Using custom MatLab scripts, the GCamp6s signal (470 nm laser) was normalized to the isosbestic control 405 nm laser signal to control for photobleaching and motion artifacts. Change in fluorescence (dF/F) was

calculated by subtracting the GCamp6s signal following stimulation from the average of GCamp6s signal for 15 seconds prior to stimulation. The area under the curve was calculated for the ten seconds directly following stimulus application. The day after baseline recordings, animals underwent spared nerve injury surgery (SNI, described below) to induce neuropathic pain. Twenty-one days following SNI, the fiber photometry protocol was repeated in the same animals to collect post SNI responses of glutamatergic neurons in the PBN to mechanical stimuli. The same fiber photometry protocol was conducted again 2 hours after intraperitoneal injection of either C2230 (10 mg/kg) or vehicle. The behavior and recording paradigm were repeated in a cross over design in a randomized order. Following the completion of the experiment, animals were transcardially perfused with ice cold 1x PBS and 10% neutral buffered formalin (Cat# SF98-4, Fisher Scientific, Waltham, MA) before brains were extracted for verification of viral infection and fiberoptic placement. 30 µm thick coronal brain sections were obtained on a cryostat and stored at 4°C. To visualize GCamp6s expression we performed immunohistochemistry for GFP. Briefly, sections were washed 3 times in PBS for 5 minutes, incubated in normal goat serum (Cat# 5425, Cell Signaling Technology, Danvers, MA) based blocking buffer (PBS with 5% normal goat serum 0.1% Triton X-100) for one hour, and incubated in primary antibody (Rabbit anti-GFP 1:1000 in blocking buffer, Cat# AB3080, Millipore Sigma, St. Louis, MO) overnight at room temperature on an orbital shaker. Sections were then washed 3 times in PBS with 0.1% Triton X-100 for 5 minutes, incubated for 1.5 hours in secondary antibody (goat anti rabbit AlexaFluor 488, Cat# A11008, Invitrogen, Waltham, MA), and washed again in PBS before being mounted on SuperFrost Plus microscope slides (Cat# 22-037-246, Fisher Scientific, Waltham, MA), coverslipped with Vectashield Plus antifade mounting medium with DAPI (H-2000-10, Vector Laboratories), and imaged at 20x on Leica DMi8 inverted widefield microscope. No animals were excluded due to post hoc target verification.

Electrophysiology

Cell lines, plasmids, site-directed mutation, and transient transfection: HEK293T (ATCC CRL-3216™) and ND7/23 (Sigma-Aldrich 92090903) cells were cultured in Dulbecco's Modified Eagle Medium (DMEM) (Invitrogen; Thermo Fisher Scientific, Inc., Waltham, MA, USA) supplemented with 10% FBS and 1% PS

(all from Gibco; Thermo Fisher Scientific, Waltham, MA, USA), maintained at 37 ℃ in an incubator with saturated humidity and 5% CO_2. cDNAs encoding the voltage-gated sodium channels (Na_Vs), voltage-gated potassium channels (K_Vs), voltage-gated calcium channels (Ca_Vs), and Ca_V auxiliary subunit (α2δ1 and β3) were cloned into pCDNA3.1 or pCMV mammalian expression vectors. Plasmid transient transfection was carried out using lipofectamine 2000 following the manufacturer's instructions (Invitrogen; Thermo Fisher Scientific, Inc., Waltham, MA, USA). Briefly, to express different Na_V subtypes for currents recording, 3 μg of Na_V1.3*(Rattus norvergicus)*, Na_V1.4*(Rattus norvergicus)*, Na_V1.5*(Homo sapiens)*, or Na_V1.7*(Homo sapiens)* plasmid was transfected into HEK293T cells, or 3 μg of Na_V1.8*(Rattus norvergicus)* or Na_V1.9*(Homo sapiens)* plasmid into ND7/23 cells. For expressing different K_V subytpes for currents recording, 0.5 – 1 μg (depending on the expression level of each channel subtype) of K_V1.3*(Homo sapiens)*, K_V1.5*(Homo sapiens)*, K_V2.1*(Rattus norvergicus)*, K_V3.1*(Homo sapiens)*, K_V3.2*(Homo sapiens)*, K_V3.4*(Homo sapiens)*, K_V4.1*(Rattus norvergicus)*, K_V4.2*(Mus musculus)*, or K_V4.3*(Mus musculus)* plasmid was transfected into HEK293T cells. Similarly, for expressing different Ca_V subtypes for currents recording, 2.5 μg of Ca_V2.2*(Rattus norvergicus)*(with 1 μg α2δ1 and 1 μg β3), Ca_V1.2*(Mus musculus)*(with 1 μg α2δ1 and 1 μg β3), Ca_V1.3*(Rattus norvergicus)* (with 1 μg α2δ1 and 1 μg β3), Ca_V3.1*(Homo sapiens)*, Ca_V3.2*(Homo sapiens)*, or Ca_V3.3*(Rattus norvergicus)* plasmid was transfected into HEK293T cells. Ca_V2.2 mutants were made by site-directed mutation as described in our previous study (1). In all of these ion channel plasmid transfections, 0.5 μg of pEGFP-N1 was co-transfected to express green fluorescence protein for identifying positively transfected cells in patch-clamp recordings. Six hours after transfection, cells were seeded onto poly L-lysine (PLL)-coated coverslips, and patch-clamp analysis was conducted 24-36 hours post transfection.

<u>Whole cell currents recording of heterologously expressed Na_V, K_V, and Ca_V channels:</u> Whole-cell patch clamp recordings was performed using standard procedures. Briefly, artifactual capacitance effect was reduced by sequential fast and slow capacitance compensation using the computer–controlled circuit of the amplifier. The series resistance after break-in was kept less than 10 MΩ to minimize voltage error in the recording circuit and 80% series resistance compensation was used with a speed value of 10 μs.

For Na_V currents recording, the standard pipette solutions contains (in mM): 140 CsF, 10 NaCl, 1 EGTA, and 10 HEPES (pH = 7.4; mOsm/L = 290 - 310); and the corresponding bath solutions contains (in mM): 140 NaCl, 5 KCl, 2 $CaCl_2$, 1 $MgCl_2$, 10 D-Glucose, and 10 HEPES (pH = 7.4; mOsm/L = 310 - 320).

For K_V currents recording, the standard pipette solution contains (in mM): 140 KCl, 2.5 $MgCl_2$, 11 EGTA, and 10 HEPES (pH = 7.4; mOsm/L = 290 - 310); and the corresponding bath solution contains (in mM): 145 NaCl, 2.8 KCl, 2 $CaCl_2$, $MgCl_2$, 10 D-Glucose, and 10 HEPES (pH = 7.3, mOsm/L = 310 - 320).

For Ca_V currents recording, the standard pipette solutions contains (in mM): 150 $CsCl_2$, 5 MgATP, 5 mM BAPTA, and 10 HEPES (pH 7.3, mOsm/L = 290-310); and the corresponding bath solution contains (in mM): 110 N-methyl-d-glucamine, 10 $BaCl_2$, 30 tetraethylammonium chloride, 10 D-glucose, and 10 HEPES (pH 7.3, mOsm/L = 310-315).

<u>Acute dissociation and culture of rat DRG neurons:</u> Thoracic and lumbar DRGs were dissociated as described previously (2, 3). Female Sprague-Dawley rats were euthanized according to institutionally approved procedures. Briefly, DRGs were collected, trimmed at their roots, and enzymatically digested in DMEM (Cat#11965, Thermo Fisher Scientific, Waltham, MA) media with neutral protease (1.04 mg/mL, Cat#LS02104, Worthington, Lakewood, NJ) and collagenase type I (1.66 mg/mL, Cat#LS004194, Worthington, Lakewood, NJ) for 50 min at 37 ºC under gentle agitation. The dissociated DRG neurons were gently centrifuged to collect cells and resuspended in complete DRG media (DMEM containing 1% PS (Gibco), and 10% FBS (Hyclone, Logan, UT)). Cells were seeded on poly-D-lysine-coated 12 mm coverslips, maintained under standard cell culture conditions (37 ℃, 5% CO_2, saturated humidity) until patch-clamp analysis.

<u>Acute dissociation and culture of rat and marmoset TG neurons:</u> TGs were collected and enzymatically digested in DMEM/F12 media (cat. no. 11765, GIBCO) with Collagenase type I (1 mg/mL, Cat#LS004194, Worthington, Lakewood, NJ) and neutral protease (0.625 mg/mL, Cat# LS02104, Worthington, Lakewood, NJ) for 25 min at 37 ºC under gentle agitation. The neurons were washed twice by centrifugation at 800 g for 7 minutes with TG media (DMEM/F12 containing 1% PS (Gibco) and 10% FBS (Hyclone, Logan, UT)), and seeded on poly-D-lysine-coated 12 mm coverslips, maintained under standard cell culture conditions until patch-clamp analysis.

Whole cell patch clamp recordings of rat and marmoset DRG and TG neurons: All recordings were done using procedures adapted from our prior work (3-6). Acutely dissociated neurons from Sprague Dawley rats with a capacitance value below 30 pF, which has been historically associated with the population of small-diameter DRG neurons, were used (7). For calcium current recordings, the internal pipette solution consists of (in mM): 150 $CsCl_2$, 5 MgATP, 5 mM BAPTA, and 10 HEPES (pH 7.3, mOsm/L = 290-310) and external solution contains (in mM): 110 N-methyl-d-glucamine, 10 $BaCl_2$, 30 tetraethylammonium chloride, 10 D-glucose, and 10 HEPES (pH 7.3, mOsm/L = 310-315). To isolate N-type calcium currents, DRG neurons were treated with a Ca_V inhibitor cocktail: Nifedipine (10 μM, L-type), SNX482 (200 nM, R-type), ω-agatoxin (200 nM, P/Q-type), TTA-P2 (1 μM, T-type). Total calcium currents in TG neurons were recorded in a manner similar to that in DRG neurons.

All neurons were interrogated with a current-voltage (I-V) protocol. The I-V protocol is as follows: From a holding potential of -60 mV, cells were depolarized with 200-millisecond voltage steps over a range of -70 to +60 mV in +10 – mV increments. In experiments utilizing C2230, the compound was added at the external solution at a final concentration of 5, 10, 20 or 50 μM as indicated. For the paired pulse protocol shown in Figure 3D, the pulse sequence was: +10 mV/15 ms → -90 mV/200 ms →+100 mV/100 ms→-90 mV/10 ms→+10 mV/15 ms. The cells were hold at -90 mV.

Rat spinal cord slices for patch-clamp: Post-natal day 8 to 12 male rat pups (Sprague-Dawley) were anesthetized with isoflurane (2-4%). 2% lidocaine was injected to both sides of lumbar vertebrae (L3-L6). The lumbar spinal cord was exposed by performing a mid-thoracic to low-lumbar laminectomy and placed in oxygenated cutting aCSF (80 mM NaCl, 2.5 mM KCl, 1.25 mM NaH_2PO_4, 0.5 mM $CaCl_2$, 3.5 mM $MgCl_2$, 25 mM $NaHCO_3$, 75 mM sucrose, 1.3 mM ascorbate, 3.0 mM sodium pyruvate; pH 7.4, 310 mOsm). 400 μm-thick transverse slices were cut on a vibratome (VT1200s) and incubated in oxygenated recording solution (125 mM NaCl, 2.5 mM KCl, 2 mM $CaCl_2$, 1 mM $MgCl_2$, 1.25 mM NaH_2PO_4, 26 mM $NaHCO_3$, 25 mM D-glucose, 1.3 mM ascorbate, 3.0 mM sodium pyruvate; pH 7.4, 320 mOsm) for 45 min at 37°C then 1 hour at room temperature. Patch pipettes were pulled from borosilicate glass capillaries using a four-step micropipette puller (P-90, Sutter Instruments, Novato, CA) to obtain a resistance ranging from 8 to 10 MΩ and loaded with pipette solution (120 mM potassium gluconate, 20 mM KCl, 2 mM $MgCl_2$, 2 mM Na_2-ATP, 0.5 mM Na-GTP, 0.5 mM EGTA, and 20 mM HEPES; pH 7.28, 310 mOsm).

Spinal cord slices were placed in the recording chamber and perfused with oxygenated recording solution (flow rate: 3-4 mL/min). The *substantia gelatinosa* (lamina I/II) was visualized by infrared differential interference contrast (DIC) video microscopy on a FN1 upright microscope (Nikon, Tokyo, Japan) equipped with a 3.40/0.80 water-immersion objective and a CCD camera.

Evoked excitatory postsynaptic current (eEPSC) recordings: eEPSC in spinal cord slice were recorded under whole-cell configuration that was obtained in voltage-clamp mode. The membrane potential was held at -60 mV using the Patchmaster software combined with the patch clamp amplifier (EPC10; HEKA Elektronik). For EPSC recording, 10 μM of bicuculline methiodide and 1 μM of strychnine were added to the recording solution to block γ-aminobutyric acid-activated (GABA) and glycine-triggered currents respectively. The access resistance (15-20 MΩ) was monitored periodically by applying step pulses (5 mV, 50 ms). For eEPSC recordings, we applied a stimulus (~200 μA, 0.1 msec) to the tract of Lissauer via a bipolar microelectrode (MicroProbes for Life Science) connected to an Flexible Stimulus Isolator (MicroProbes for Life Science).

Human DRG (hDRG) neurons culture: Human DRG suspension cells were obtained from AnaBios Corporation. Donor information is provided in Supplemental table 2.Cells were recovered with a gentle centrifugation (~350 xG) at room temperature for 3 min and the pellet was gently resuspend with 2 mL of complete DMEM/F12 media containing 1% PS, 10% horse serum (Cat#SH3007403, Cytiva HyClone, Logan, UT), 25 ng/mL hNGF (Cat#256GF100CF, Fisher scientific, Pittsburgh, PA), and 25 ng/mL GDNF (Cat#RP-8602, Thermo fisher, Waltham, MA). The cells were seeded on poly-D-lysine (0.1 mg/ml; Cat# P6407, Millipore Sigma, St. Louis, MO) and laminin (1 mg/ml; Cat#sc-29012, Santa Cruz Biotechnology, Dallas, TX)-coated 12-mm glass coverslips and incubated at 37°C. Half of the culture media is replaced with fresh media every 3 days. All cultures were used within 96 hours.

Whole cell patch clamp recordings of hDRG neurons: The composition of the external solution to record total calcium currents is (in mM): 100 Choline-Cl, 3 KCl, 5 $BaCl_2$, 0.6 $MgCl_2$, 10 D-Glucose, 10 HEPES (pH 7.4, mOsm/L = 325 Osm). The internal solution contains (in mM): 100 CsCl, 5 NaCl, 40 TEA-Cl, 2 $MgCl_2$, 1 $CaCl_2$, 2 Mg-ATP, 1 GTP, 11 EGTA, 10 HEPES, (pH7.4, mOsm/L = 310) (8). hDRG neurons were interrogated with current-voltage (I-V) protocols. From a holding potential of −60 mV, cells were depolarized with 200-millisecond voltage steps over a range of -70 to +60 mV in +5 mV increments.

In experiments utilizing C2230, the compound was added to the external solution at a final concentration of 20 μM.

Construction of current-voltage (I-V) curves: Normalization of currents to each cell's capacitance (pF) was performed to collect the current density data. For I-V curves, functions were fitted to data using a non-linear least squares analysis. I-V curves were fitted using double Boltzmann functions:

$$f = a + g1/(1+\exp((x-V_{1/2}1)/k1)) + g2/(1+\exp(-(x-V_{1/2}2)/k2))$$

where x is the pre-pulse potential, $V_{1/2}$ is the mid-point potential and k is the corresponding slope factor for single Boltzmann functions. Double Boltzmann fits were used to describe the shape of the curve, not to imply the existence of separate channel populations. Numbers *1* and *2* simply indicate first and second mid-points; *a* along with *g* are fitting parameters.

Pain models

L4/L5 spinal nerve ligation in mice: All mouse surgeries were conducted following the method previously described (9). In brief, the animals were anesthetized using isoflurane (5% for induction and 2.5% for maintenance). The left L4 and L5 spinal nerves were exposed and ligated using 7-0 silk suture in a region distal to the DRG. Muscle and fascia were closed in layers using 5-0 absorbable suture, and the skin was autoclipped. Throughout the study, all mice were regularly monitored for general health, and no mice were excluded from the study.

L5/L6 spinal nerve ligation in rats: Rats were anesthetized with isoflurane (5% for induction and 2.5% for maintenance). The lower half of the animal's back was shaved. After surgical preparation, the left L5 and L6 spinal nerves were exposed by removing the paraspinal muscles and ligated with a 5–0 silk suture in a region distal to the DRG (10). After hemostasis was confirmed, muscle and fascia were closed in layers using 5-0 absorbable suture, and the skin was closed with wound clips.

Spared nerve injury in mice: SNI was performed as previously described (11). Mice were anesthetized using isoflurane (5% for induction and 2.5% for maintenance). The hind left limb was shaved and disinfected with three alternating wipes of 70% ethanol and 2% chlorhexidine gluconate. A small incision was made in the skin of the hind left leg, and the underlying muscle was separated via blunt

dissection to expose the sciatic nerve branches. The peroneal and tibial nerves were ligated with 6-0 silk sutures and carefully transected, ensuring the sural nerve was left intact. After nerve manipulation, the muscle tissue was loosely sutured using 5-0 nylon sutures, and the skin was closed with 9 mm wound clips. A topical triple antibiotic ointment (Neosporin) was applied to the wound. Wound clips were removed approximately 10 days post-surgery, with behavioral experiments starting 21 days after surgery. Sham surgeries followed the same procedure, including nerve exposure, but without ligation or transection.

Chronic constriction injury of the rat's infraorbital nerve: Prior to surgical procedures, rats were anesthetized with intraperitoneal injections of ketamine (50 mg/kg) and xylazine (7.5 mg/kg) solution. A single investigator performed the surgeries to minimize variability. Unilateral chronic constriction injury to the infraorbital nerve (CION) was used to induce trigeminal neuropathic pain in rats as previously described (12). Briefly, an approximately 1 cm long incision was made along the left gingivobuccal sulcus beginning just proximal to the first molar. Infraorbital nerve was exposed (~0.5 cm) and freed from the surrounding tissue. Two chromic gut (4-0) ligatures were loosely tied around the exposed nerve. The incision was closed with the absorbable sutures.

Induction of osteoarthritis-like pain: Monoiodoacetate (MIA)-induced osteoarthritis-like pain model was established as previously described (13). On the day of injection, the monoiodoacetate (MIA) solution was freshly prepared in sterile saline by dissolving 1 mg of sodium Iodoacetate (Cat#I9148, Sigma, St. Louis, MO) in 10 µL of saline. Mice were anaesthetized using Isoflurane (5% for induction and 2.5% for maintenance). The animal was then placed on its back in dorsal recumbency, and the area around the knee of the left hind leg was shaved. The knee was stabilized and fixed in a slightly bent position, and the patellar tendon was visualized as a white line beneath the skin. A 10 µL of MIA solution was injected intraarticularly into the joint-space using a 30G insulin-syringe (BD Micro-Fine Plus Demi, 0.3ml (30G)) by inserting the needle perpendicularly through the skin and tendon just below the patella.

Compound administration

Intraperitoneal administration: Intraperitoneal delivery was carried out using a 30-gauge, 0.3-inch needle, inserted into the lower left quadrant of the abdomen. The needle was positioned parallel to the backbone at a 45° angle to the abdominal wall. Behavioral assessments were conducted at 1-, 2-, 3-, 4-, 5-, and 6-hours following administration to measure paw withdrawal threshold and cold aversion time. Additionally, assessments of calcium dynamics of glutamatergic neurons, CPA, and distance traveled were performed 2 h post-administration.

Intranasal administration: At 22 days post-CION, half of the rats received an intranasal C2230 (200 µg in 20µL isotonic saline) and the remaining half received 20 µL of isotonic saline (vehicle-control). Intranasal delivery was performed with a pipette and a disposable plastic tip. Immediately after administration, the head of the animal was held in a tilted back position for ~15 seconds to prevent loss of solution from the nare. Behavioral assessments were done at 30 minutes, 1-, 2-, and 3-hours post-administration.

Intrathecal administration: Ten days after spinal nerve ligation, C2230 was injected intrathecally (10 µg/5 µL) between L4/L5 intervertebral level into isoflurane anesthetized male rats, and behavior was measured every hour for 5 hours.

Behavioral testing

Measurement of mechanical allodynia in the hindpaw: Mechanical allodynia was assessed by measuring rats' and mice's paw withdrawal threshold in response to probing with a series of fine calibrated filaments (von Frey, Stoelting, Wood Dale, IL). Rats or mice were placed in suspended plastic cages with a wire mesh floor, and each von Frey filament was applied perpendicularly to the plantar surface of the paw. The "up-down" method (sequential increase and decrease of the stimulus strength) was used to determine the withdrawal threshold. Dixon's nonparametric method was used for data analysis, as described by Chaplan *et al* (14). Data were expressed as the 50% paw withdrawal threshold. Mechanical allodynia was manifested as a decrease in paw withdrawal threshold from baseline.

Measurement of cold sensitivity in the hindpaw: Mice were individually caged on mesh metal flooring for 30 minutes prior to testing. 10 µL of acetone (Sigma-Aldrich, MO, USA) was applied onto plantar surface of each hind paw via pipette or a syringe connected to PE-90 tubing. The cumulative time spent displaying pain-like behaviors like, licking, guarding, slapping, flinching, or shaking the hind paw was recorded for 30 seconds for MIA and 60 seconds for SNL/SNI mice. Measures were repeated three times and averaged. An increase in response duration from baseline is indicative of development of cold allodynia.

Measurement of facial mechanical allodynia: von Frey detection threshold was measured by applying von Frey monofilaments delivering calibrated force ranging from 0.0008 g to 1 g, corresponding to log units from 1.65 to 4.08 (EXACTA Precision & Performance monofilaments, Stoelting), within the ION territory in ascending order of intensity (15). The lowest filament that evoked a withdrawal response (brisk head withdrawal, touching or scratching of the facial regions) was designated as the withdrawal threshold. A decrease in the withdrawal threshold from baseline is indicative of the development of hypersensitivity.

Pinprick assay: Pinprick response score was measured by scoring the response to stimulation with a blunted acupuncture needle applied within the vibrissal pad of the rats. The scores were assigned as follows: 0=no response, 1=non-aversive response, 2=mild aversive response, 3=strong aversive response, 4=prolonged aversive behavior (16, 17). An increase in the response score is indicative of the development of hypersensitivity.

Conditioned Place Aversion: CPA was conducted in a two-chamber device based on the protocols from Zhou *et al* 2018 (18). The protocol includes 4 x 10 min of sequential tests of preconditioning (10 min), conditioning (2 x 10 min) and testing (10 min). During preconditioning, the animal is allowed free access to two connected chambers (30 x 30 x 19 cm), each associated with a scented lip-balm applied to the walls. Immediately following preconditioning, a divider was applied between the chambers, and the rats were conditioned to either stimuli or no-stimuli for 10 min in each chamber. The stimuli consisted of repeated stimulation with a 15 g von Frey filament (vF) every 30 s for the 10 min that the subject was contained in that chamber, while no stimuli (NS) was applied in the other chamber. The order and side of conditioning was alternated between subjects. Following the conditioning, the divider was removed, and

the rat was allowed free access to both chambers for the 10 min test. Animal movements in each chamber were recorded by a camera above and analyzed using ANYmaze. The duration of time spent in each chamber and the distance moved was recorded during preconditioning and test. Decreased time spent in a chamber during the test versus preconditioning indicated avoidance for that chamber and was calculated as a CPA-score; time in vF-chamber during preconditioning – time in vF-chamber during test.

Rats were exposed to SNL-injury approximately two weeks prior to the CPA-test. On the day of the CPA test, they were administered freshly prepared vehicle (PBS) or 10 mg/kg C2230, and two hours later (the peak of effect in the time-course studies), they were tested in the CPA paradigm.

Hemodynamic measurements

C57BL/6 male mice were used for terminal hemodynamic measurements. The mice were randomized and injected intraperitoneally with either C2230 (10 mg/kg) or vehicle (n = 4 - 5 in each group) . After 2 hrs of C2230 injection, the hemodynamic parameters such as blood pressure and heart rate of all mice were evaluated as previously described (19). Briefly, the mice were anesthetized with 0.75 mg/g of 2.5% Avertin (a mixture of tert-amyl alcohol and 2,2,2-tribromoethanol; Sigma-Aldrich, St. Louis, MO) and a radio telemetry catheter (PA-C10; Data Sciences International (DSI), St. Paul, MN) was advanced into the right carotid artery. The BP data tracings was recorded invasively using Dataquest ART (DSI; St. Paul, MN) software. The data such as mean arterial blood pressure (MABP) and mean heart rate (MHR) were analyzed with Dataquest ART software supplied along with the DSI system. After the completion of the measurements, which took less than 10 minutes per animal, the mice were euthanized.

Pharmacokinetic study

Experimental design and sample collection: Young, adult male C57BL/6 mice, weighing 20-25 g, were procured from Envigo (Indianapolis, IN, USA) and group housed in home cages (N = 5). The animals were acclimatized for a minimum period of 3 days before the experiment at room temperature and controlled humidity while on a regular 12 h light-dark cycle. The mice were randomly divided into 5

groups of N = 3 each and C2230 was administered intraperitoneally at a dose of 10 mg/kg. The mice were euthanized at 0.5, 1, 2, 4, and 6 h, post-dose, and blood and brain samples were collected. Plasma was harvested by centrifuging the blood at 10000 rpm for 10 min and stored frozen at –80°C until bioanalysis. Pharmacokinetic parameters were calculated using non-compartmental analysis of sparse concentration-time data by Phoenix 6.4 (Certara, Princeton, NJ, USA).

Sample preparation and LC-MS/MS analysis: A simple protein precipitation method was used for the extraction of C2230 from plasma and brain homogenate samples. The brain samples were homogenized in water at a ratio of 1:2 before processing. A 30 µL of the plasma/brain homogenate sample was cleaned up with methanol containing 0.1 % formic acid and 20 ng/mL of phenacetin as internal standard (I.S.) at a ratio of 1:4 and vortex-mixed for 5 minutes followed by filtration through 0.45 µm filter plates (Millipore Solvinert® plates) by centrifugation at 1500 rpm for 5 min.

Bioanalysis was carried out using a Waters Acquity Class I Plus ultra-performance liquid chromatography coupled with a Waters Xevo TQ-XS triple quadrupole mass spectrometer (UPLC-MS/MS) (Waters, Milford, MA, USA). The chromatographic separation was achieved using Acquity UPLC BEH C18 column (2.1 mm x 50 mm, 1.7 µm) and the mobile phase consisted of 0.1% formic acid (A) – acetonitrile (B) with a gradient program of 90 % A held for 0.5 min, then decreased to 10% reaching 2.0 min and sharply decreased back to the initial conditions by 2.1 min and maintained until 2.5 min. The column and autosampler temperatures were kept at 40°C and 10°C, respectively. The mobile phase was delivered at a flow rate of 0.35 mL/min and the injection volume was set to 4 µL. The MassLnyx software version 4.2 was used for instrument control and TargetLynx for data analysis. The mass spectrometer was operated in positive ion mode and detection of the ions was performed in the multiple reaction monitoring (MRM) mode, monitoring transitions of *m/z* 322.24 precursor $[M+H]^+$ to the *m/z* 198.06 product ion for C2230 and *m/z* 180.12 precursor $[M+H]^+$ to *m/z* 110.03 product ion for I.S. (phenacetin). Test samples were analyzed with freshly prepared calibration and quality control standards for a linearity range of 1-200 ng/mL or ng/g in respective plasma and brain homogenates.

Supplemental figures and legends

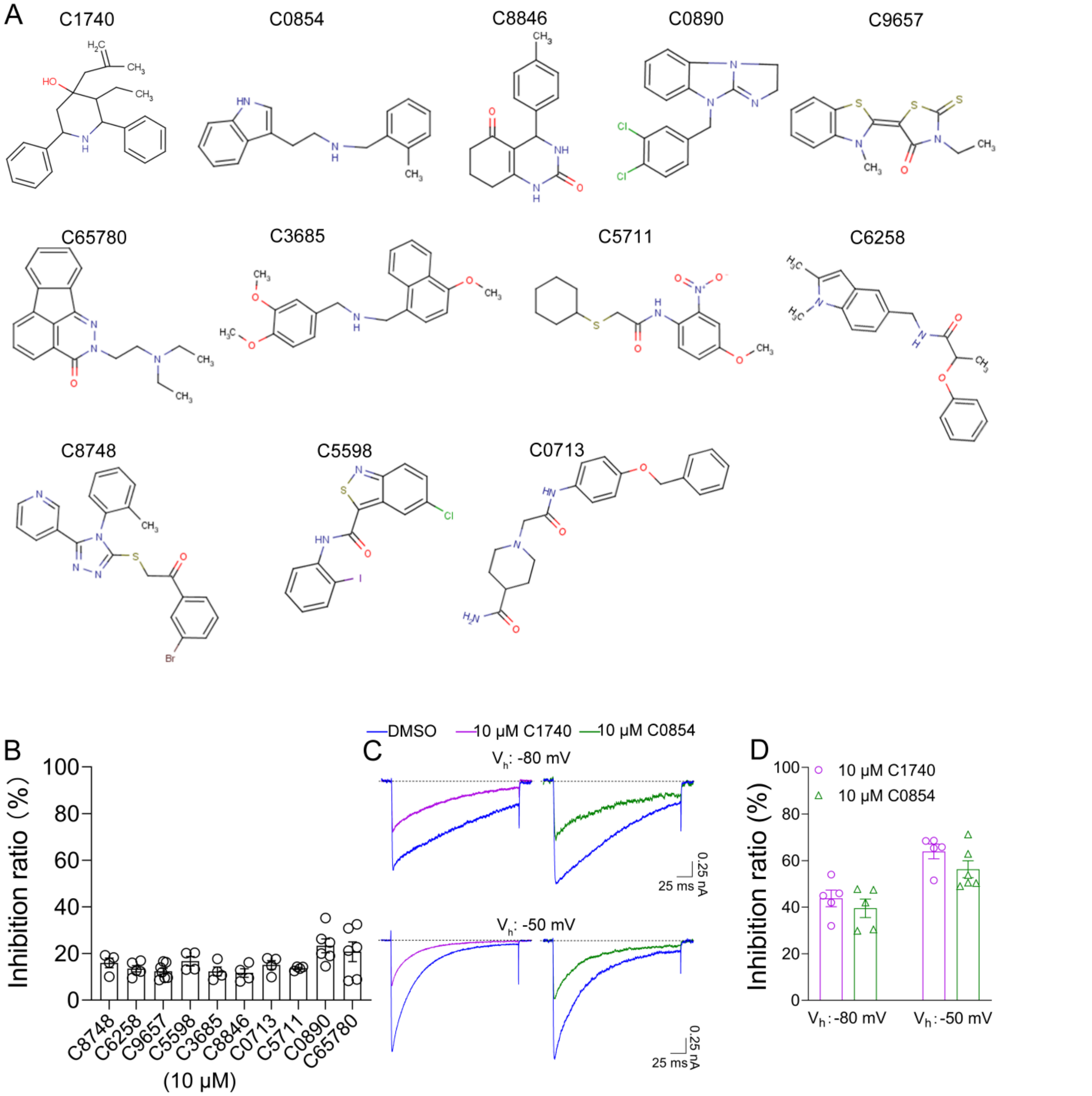

Supplemental Figure 1. (A) Structures of 12 low-potency Cav2.2 inhibitors identified from the library. **(B)** inhibition ratio of these 12 compounds (n = 4 - 7). Typical traces **(C)** and bar graphs **(D)** showing the inhibition of 10 μM C1740 or 10 μM C0854 on heterologously expressed Cav2.2 currents at different holding potentials. The inhibition ratio is determined as 43.8 ± 3.6% and 63.9 ± 3.2% for C1740, and 39.5 ± 4.0% and 56.3 ± 3.6% for C0854, at -80 mV and -50 mV, respectively (n = 5 - 6). Using the simplified

Hill equation, the roughly calculated IC_{50} values were 13.5 ± 2.1 μM and 5.8 ± 0.9 μM for C1740, and 16.4 ± 2.8 μM and 8.1 ± 1.0 μM for C0854, at the holding potentials of -80 mV and -50 mV, respectively.

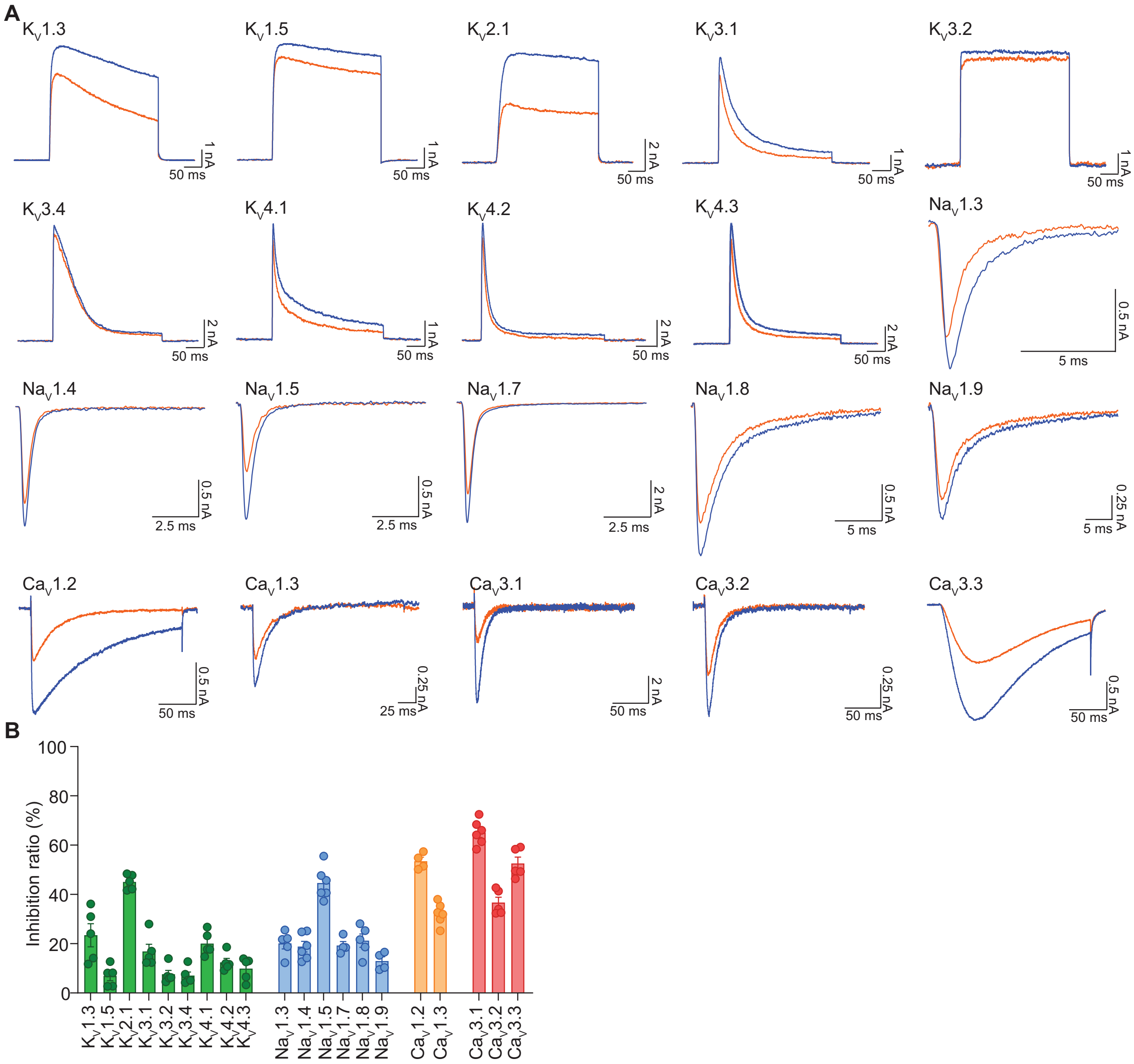

Supplemental Figure 2. (A) Shown are representative current traces demonstrating the effect of 20 μM C2230 on different K_V, Na_V, and Ca_V channels heterologously expressed in HEK293T cells (blue: control; red: 20 μM C2230) (n = 4 - 6). **(B)** Bar graphs with data points representing the summary inhibition ratio of 20 μM C2230 on the currents of K_V, Na_V, and Ca_V channels, as indicated (n = 4 - 6).

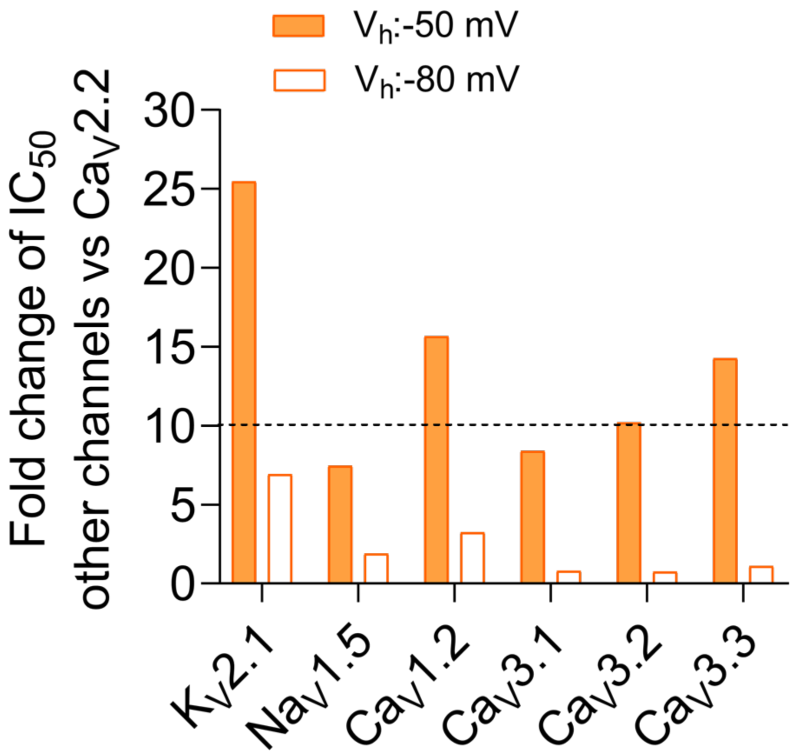

Supplemental Figure 3. Summary of fold-changes, versus, $Ca_V2.2$, in IC_{50} values of inhibition by C2230 at holding potentials of -50 mV and -80 mV. Related to data in Figure 1 F and G. Dose-response relationships of C2230 inhibiting the heterologously expressed $K_V2.1$, $Na_V1.5$, $Ca_V1.2$, $Ca_V3.1$, $Ca_V3.2$, and $Ca_V3.3$ channels were calculated from 5 - 9 cells each.

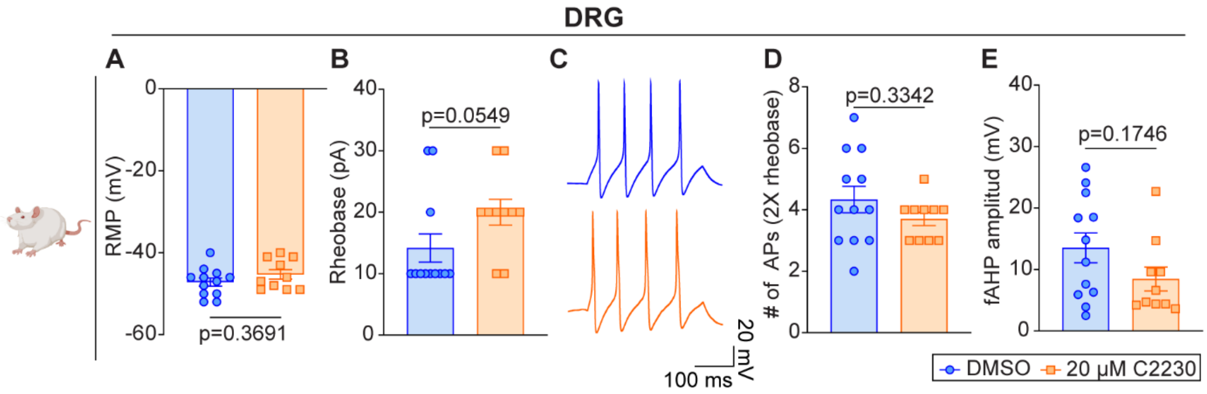

Supplemental Figure 4. C2230 does not affect DRG neuron excitability. (A) Bar graph showing DRG neuron resting membrane potential (RMP). **(B)** Bar graph representing the minimum current necessary to elicit a single action potential or rheobase. **(C)** Representative traces of action potentials from cells treated with 0.1% DMSO (blue) or 20 μM C2230 (orange). **(D)** Bar graph illustrating the number of action potentials elicited upon stimulation at 2 times (2X) the rheobase. **(E)** Bar graph depicting the fast hyper-polarization (fAHP). *P* value as indicated; Mann-Whitney tests; n = 10-12 cells per condition from 3 independent experiments. Data are presented as MEAN ± SEM. p values as indicated, see Supplemental Table 3 for full statistical analysis.

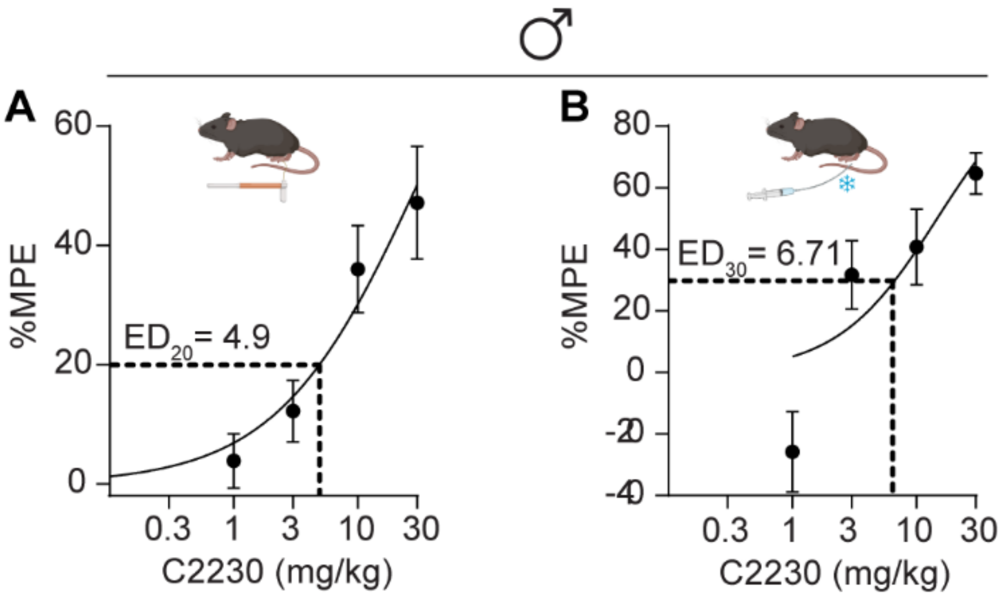

Supplemental Figure 5. C2230's maximum possible effect (MPE) is dose-dependent in male mice with spinal nerve ligation. Related to Figure 6B and 6F of the manuscript. **(A)** Dose-response curve of the pencentage maximum possible effect (%MPE) of C2230 on tactile allodynia. **(B)** Dose-response curve of %MPE of C2230 on cold allodynia. ED_{20} and ED_{30} were calculated as they represent half of the maximal effect observed in the experiments. n= 8 mice per group; values are expressed as MEAN ± SEM.

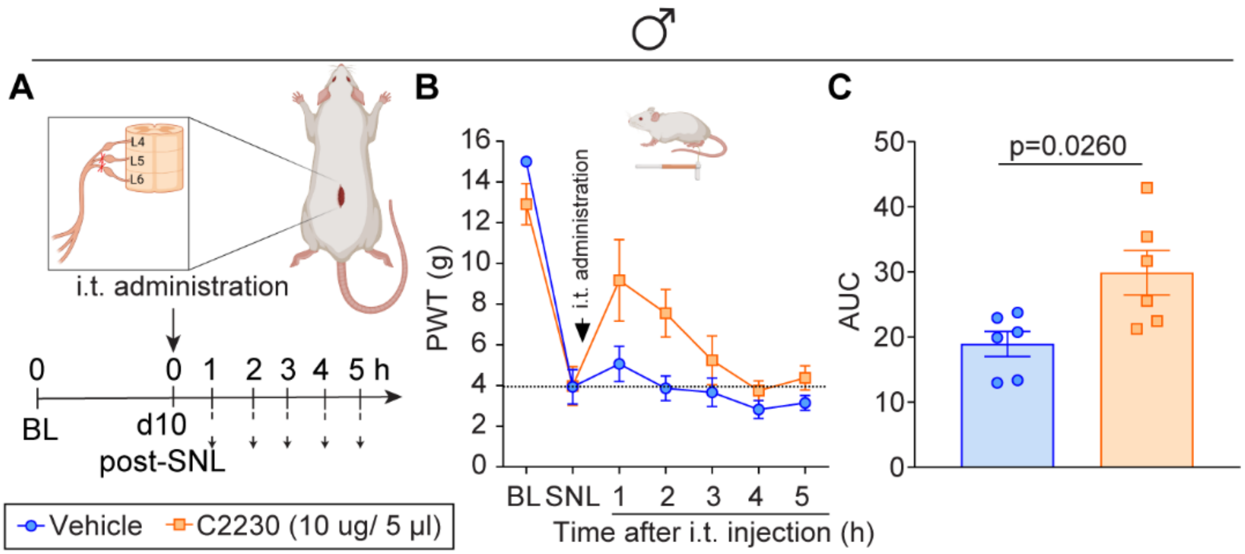

Supplemental Figure 6. Intrathecal administration of C2230 reverses pain-like behaviors in rats with spinal nerve ligation (SNL). (A) SNL model schematic and timeline of the experimental approach used to determine the antinociceptive effects induced by C2230 on mechanical allodynia. **(B)** Time course of the paw withdrawal threshold (PWT) measured after i.t. administration of vehicle or C2230 (10 µg/ 5 µL). BL represents baseline measurement before SNL. **(C)** Quantification of the area under the curve (AUC) of panel B between the SNL timepoint and 5 h after i.t. injection. n= 6 rats per group; values are expressed as MEAN ± SEM. p value as indicated, see Supplemental Table 3 for full statistical analysis.

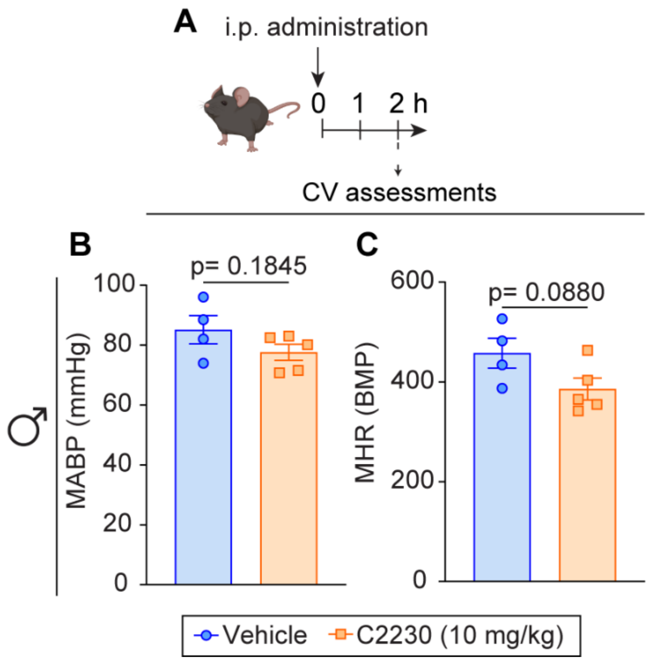

Supplemental Figure 7. C2230 does not affect cardiovascular (CV) function in male mice. (A) Timeline of the experimental approach used to determine mean arterial blood pressure (MABP) and mean heart rate (MHR), 2 h after i.p. administration of C2230 (10 mg/kg) to naïve male mice. **(B)** Bar graph showing MABP measurements in millimeters of mercury (mmHg) after i.p. administration of vehicle or C2230. **(C)** Bar graph illustrating MHR measurements in beats per minute (BPM) after i.p. administration of vehicle or C2230. n = 4 - 5 mice per group; values are expressed as MEAN ± SEM. p values as indicated, see Supplemental Table 3 for full statistical analysis.

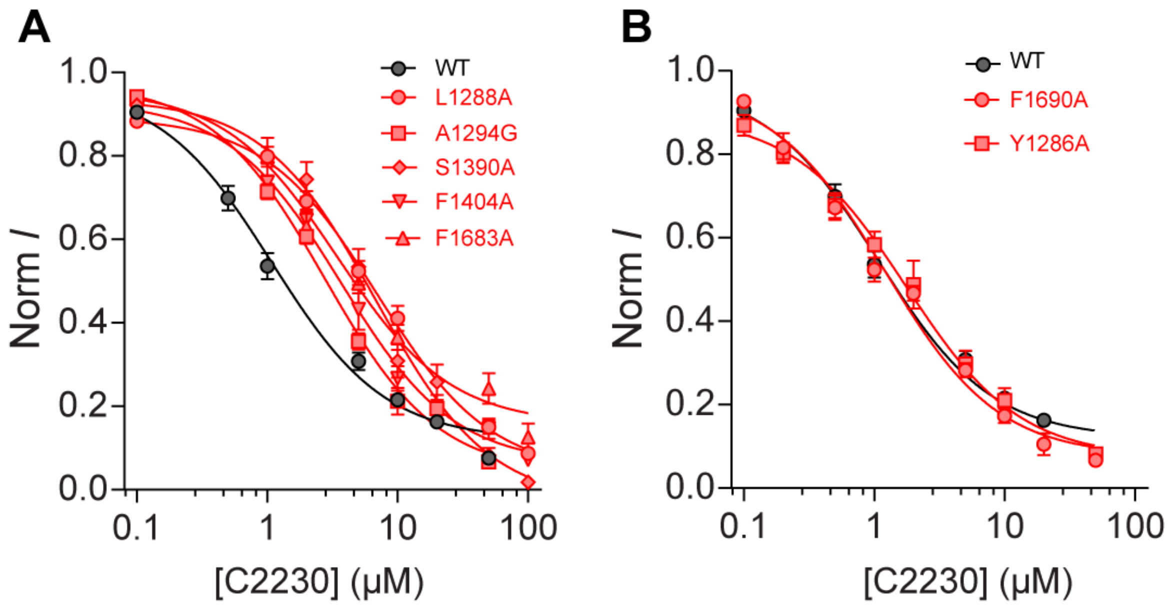

Supplemental Figure 8. Dose-response relationships of C2230-mediated inhibition of the indicated CaV2.2 alanine mutants at the holding potential of -50 mV; the wild-type CaV2.2 channel was included for comparison. (**A**) The IC_{50}s were calculated to be: 1.1 ± 0.2 μM, 7.0 ± 0.8 μM, 2.8 ± 0.3 μM, 6.8 ± 1.1 μM, 3.7 ± 0.6 μM, and 3.4 ± 0.6 μM, for wild-type (WT), L1288A, A1294G, S1390A, F1404A, and F1683A mutant channels, respectively (n = 5 – 10 cells). (**B**) The IC_{50}s were calculated to be: 1.1 ± 0.2 μM, 1.2 ± 0.2 μM, and 1.8 ± 0.3 μM, for WT, F1690A, and Y1286A mutant channels, respectively (n = 5 – 10 cells).

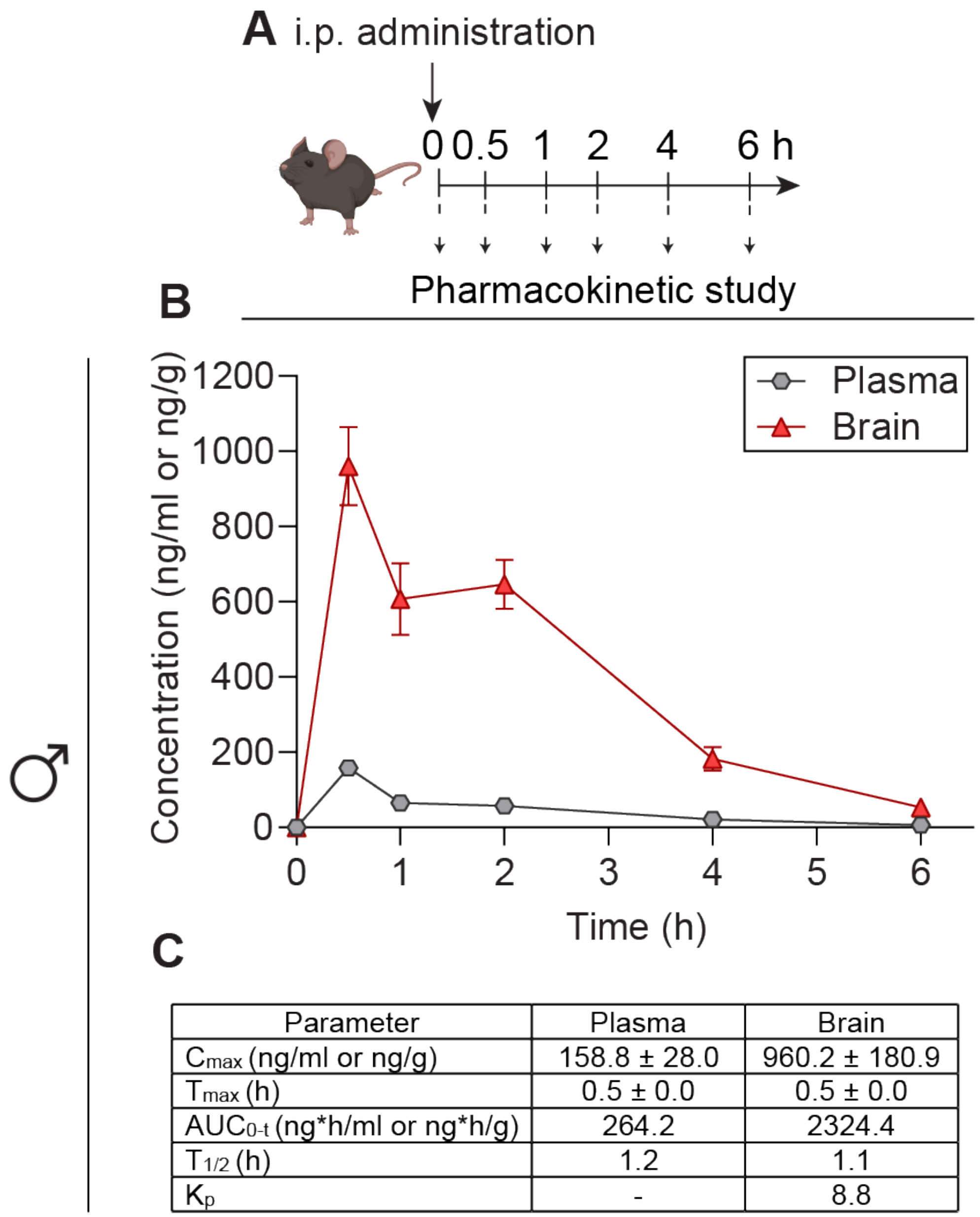

C

Parameter	Plasma	Brain
C_{max} (ng/ml or ng/g)	158.8 ± 28.0	960.2 ± 180.9
T_{max} (h)	0.5 ± 0.0	0.5 ± 0.0
$AUC_{0\text{-}t}$ (ng*h/ml or ng*h/g)	264.2	2324.4
$T_{1/2}$ (h)	1.2	1.1
K_p	-	8.8

Supplemental Figure 9. Plasma and brain concentration-time profiles and pharmacokinetic parameters of C2230 after intraperitoneal administration (10 mg/kg) in male mice. (A) Timeline of the experimental approach used to determine C2230 plasma and brain concentrations after i.p. administration to naïve male mice. **(B)** Time course of plasma and brain concentration measurements of C2230. **(C)** Table of pharmacokinetic parameters of C2230. Abbreviations: C_{max} = peak concentration, T_{max} = time to reach C_{max}, $AUC_{0\text{-}t}$ = area under the concentration-time curve up to 6 h, $T_{1/2}$ = elimination half-life, and K_p = brain to plasma AUC ratio.

Supplemental Table 1. Calculated properties of C2230.

ID	IUPAC Name	Compound Class	Mw	BBB	cLogS (7.4)	cLogP	HBD	HBA	RO5	NHOH	RotB	TPSA	QED
C2230	1-(2-tert-butyl-4-methoxyphenoxy)-3-(cyclopentylamino)propan-2-ol	1-aryloxy-3-amino-2-propanols	321.5	4.7	-2	3.3	2	4	Y	2	7	50.7	0.81

Mw, molecular weight (Da); BBB score, indicates probability of compound having CNS exposure where scores in the range [4-6] correctly predicted 90.3% of CNS drugs (20); LogS(7.4), predicted solubility (M) at pH 7.4; cLogP, predicted lipophilicity coefficient in octanol/water; HBD, number of hydrogen-bond donors; HBA, number of hydrogen bond acceptors; RO5, binary (Y/N) assignment of complying with Lipinski rule-of-5 (21); NHOH, number of polar NH and OH hydrogens; RotB, number of rotatable bonds; TPSA, topological polar surface area (Å^2); QED, Quantitative Estimate of Druglikeness where a score of 1 indicates all properties are favorable (22). Properties calculated with RDKit and ChemAxon modules.

Supplemental Table 2. Human donor information.

ID	Age	Sex	Race	Cause of death
230927DHA (AnaBios)	34	M	Filipino	Cerebral vascular accident / Intracerebral hemorrhage / Stroke
230919DHA (AnaBios)	25	F	Arab	Anoxia / Drug intoxication
AKKM235 (UNOS)	54	M	-	Anoxia / Cardiovascular

Supplemental Table 3. Full statistical analysis.

Figure panel	Assay	Statistical test; findings	Post-hoc analysis (adjusted p-value)	Number of subjects
Figure 2B	HEK293 cells-$Ca_V2.2$ peak current-IV curve	Kruskal-Wallis test<br><br>p<0.0001	Dunn's multiple comparison test<br><br>DMSO (0.1%) vs. 10 μM C2230 norm *I* to 1<br>p=0.9616<br><br>DMSO (0.1%) vs. 10 μM C2230<br>p=0.0002	DMSO (n=13)<br>C2230 norm *I* to 1 (n=13)<br>C2230 (n=13)
Figure 2D	HEK293 cells-$Ca_V2.2$ three-pulse protocol	Unpaired t-tests comparing individual timepoints	DMSO (0.1%) vs. C2230 (20 μM):<br><br>0 ms: p=0.0549<br>50 ms: p=0.0079<br>100 ms: p=0.0084<br>200 ms: p=0.0403<br>400 ms: p=0.0602<br>800 ms: p=0.1890<br>1600 ms: p=0.2132<br>3200 ms: p=0.4336	DMSO (n=13)<br>C2230 (n=12)
Figure 2E	OSI Tau of inactivation V_h= -80 mV	Paired t test	Control vs. C2230 (20 μM):<br>P<0.0001<br><br>Control vs. DMSO (0.1%):<br>p=0.4983	Control (n=10)<br>C2230 (n=10)<br><br>Control (n=8)<br>DMSO (n=8)
Figure 2F	OSI Tau of inactivation V_h= -50 mV	Paired t test	Control vs. C2230 (20 μM):<br>P<0.0001<br><br>Control vs. DMSO (0.1%):<br>p=0.9399	Control (n=10)<br>C2230 (n=10)<br><br>Control (n=8)<br>DMSO (n=8)
Figure 3C	Rat DRGs-$Ca_V2.2$ peak current density	Kruskal-Wallis test<br><br>p<0.0001	Dunn's multiple comparison test<br><br>DMSO (0.1%) vs. C2230 (5 μM):<br>p=0.3106<br>DMSO (0.1%) vs. C2230 (10 μM):<br>p<0.0001<br>DMSO (0.1%) vs. C2230 (50 μM):<br>p<0.0001	DMSO (n=28)<br>C2230, 5 μM (n=10)<br>C2230, 10 μM (n=15)<br>C2230, 50 μM (n=6)
Figure 3F	Rat DRGs-Calcium currents-paired-pulse protocol	Mann-Whitney test	DMSO (0.1%) vs. C2230 (20 μM):<br>p=0.9454	DMSO (n=6)<br>C2230 (n=7)

Figure 3I	Human DRGs-Total calcium peak current density	Mann-Whitney test	DMSO (0.1%) vs. C2230 (20 µM): p=0.0047	DMSO (n=6)<br>C2230 (n=7)
Figure 4C	Rat TGs-Total calcium peak current density	Kruskal-Wallis test<br><br>p=0.0026	Dunn's multiple comparison test<br><br>DMSO (0.1%) vs. C2230 (20 µM): p=0.0027<br>DMSO (0.1%) vs. ω-Ctx-GVIA (500 nM): p=0.0468<br>DMSO vs. C2230 + ω-Ctx-GVIA p=0.0111<br>DMSO vs. ω-Ctx-GVIA + C2230 p=0.0016<br>C2230 (20 µM) vs. ω-Ctx-GVIA (500 nM): p>0.9999<br>C2230 vs. C2230 + ω-Ctx-GVIA p>0.9999<br>C2230 vs. ω-Ctx-GVIA + C2230 p>0.9999<br>ω-Ctx-GVIA vs. C2230 + ω-Ctx-GVIA p>0.9999<br>ω-Ctx-GVIA vs. ω-Ctx-GVIA + C2230 p>0.9999<br>C2230 + ω-Ctx-GVIA vs. ω-Ctx-GVIA + C2230 p>0.9999	DMSO (n=13)<br>C2230 (n=13)<br>ω-Ctx-GVIA (n=9)<br>C2230 + ω-Ctx-GVIA (n=9)<br>ω-Ctx-GVIA + C2230 (n=10)
Figure 4D	Rat TGs-Total calcium norm / C2230 + ω-Ctx-GVIA	One-way ANOVA<br><br>p=0.0002<br>F(0,10)= 0.7321	Tukey's multiple comparisons test<br><br>DMSO (0.1%) vs. C2230 (20 µM): p=0.0003<br>DMSO (0.1%) vs. ω-Ctx-GVIA (500 nM): p=0.0008<br>C2230 (20 µM) vs. ω-Ctx-GVIA (500 nM): p=0.9897	DMSO (n=5)<br>C2230 (n=5)<br>ω-Ctx-GVIA (n=3)
Figure 4E	Rat TGs-Total calcium norm / ω-Ctx-GVIA + C2230	One-way ANOVA<br><br>p=0.0003<br>F(2,12)= 0.7155	Tukey's multiple comparisons test<br><br>DMSO (0.1%) vs. C2230 (20 µM): p=0.0002	DMSO (n=5)<br>C2230 (n=5)<br>ω-Ctx-GVIA (n=5)

			DMSO (0.1%) vs. ω-Ctx-GVIA (500 nM): p=0.0139<br>ω-Ctx-GVIA (500 nM) vs. C2230 (20 μM): p=0.0783	
Figure 4H	Marmoset TGs-Total calcium peak current density	Kruskal-Wallis test<br><br>p=0.0002	Dunn's multiple comparison test<br><br>DMSO (0.1%) vs. C2230 (20 μM): p=0.0002<br>DMSO (0.1%) vs. ω-Ctx-GVIA (500 nM): p=0.0189<br>C2230 (20 μM) vs. ω-Ctx-GVIA (500 nM): p=0.8576	DMSO (n=12)<br>C2230 (n=13)<br>ω-Ctx-GVIA (n=10)
Figure 5A	Evoked iCGRP	2-way ANOVA Fraction*treatment<br><br>Interaction: F(5,24)=3.332 p=0.0200<br><br>Fraction: F(5,24)=8.174 p=0.0001<br><br>Treatment: F(1,24)=4.774 p=0.0389	Sidak's multiple comparisons test<br><br>DMSO vs. C2230 (20 μM)<br><br>Fraction 1: p>0.9999<br>Fraction 2: p>0.9999<br>Fraction 3: p>0.9999<br>Fraction 4: p=0.0008<br>Fraction 5: p=0.9889<br>Fraction 6: p=0.9992	DMSO (n=3)<br>C2230, 20 μM (n=3)
Figure 5D	Amplitude of eEPSCs	Paired t-test	DMSO (0.1%) vs. C2230 (20 μM): p=0.0474	DMSO (n=5)<br>C2230, 20 μM (n=5)
Figure 6C	Male SNL mice-AUC Mechanical threshold	One-way ANOVA<br><br>p<0.0001<br>F(4,35)= 0.8316	Dunnett's multiple comparisons test<br><br>Vehicle vs. C2230 (1 mg/kg): p=0.2519<br>Vehicle vs. C2230 (3 mg/kg): p=0.0177<br>Vehicle vs. C2230 (10 mg/kg): p<0.0001<br>Vehicle vs. C2230 (30 mg/kg): p<0.0001	n=8 for all groups.

Figure 6E	Female SNL mice-AUC Mechanical threshold	One-way ANOVA<br><br>$p<0.0001$<br>$F(2,21)= 1.099$	Dunnett's multiple comparisons test<br><br>Vehicle vs. C2230 (10 mg/kg): $p=0.0013$<br>Vehicle vs. C2230 (30 mg/kg): $p<0.0001$	n=8 for all groups.
Figure 6G	Male SNL mice-AUC aversion time	One-way ANOVA<br><br>$p<0.0001$<br>$F(4,35)= 1.818$	Dunnett's multiple comparisons test<br><br>Vehicle vs. C2230 (1 mg/kg): $p=0.9023$<br>Vehicle vs. C2230 (3 mg/kg): $p=0.1262$<br>Vehicle vs. C2230 (10 mg/kg): $p=0.0041$<br>Vehicle vs. C2230 (30 mg/kg): $p<0.0001$	n=8 for all groups.
Figure 6I	Female SNL mice-AUC aversion time	One-way ANOVA<br><br>$p<0.0001$<br>$F(2,21)= 1.096$	Dunnett's multiple comparisons test<br><br>Vehicle vs. C2230 (10 mg/kg): $p<0.0001$<br>Vehicle vs. C2230 (30 mg/kg): $p<0.0001$	n=8 for all groups.
Figure 7B	SNI Chronic Mechanical threshold	Two-Way repeated measures (RM) ANOVA<br><br>Time x Injury + Drug: $F(6,64)=22.58$ $p<0.0001$<br><br>Time: $F(1.447,46.31)=4.97$ $p=0.0346$<br><br>Injury + Drug: $F(3,32)=261.3$ $p<0.0001$<br><br>Subject $F(32,64)=3.139$ $p<0.0001$	Dunnet's multiple comparisons test<br><br>Week 3, SNI + C2230, 0 vs. 120min: $p<0.0001$<br><br>Week 6, SNI + C2230, 0 vs. 120min: $p<0.0001$<br><br>Week 9, SNI + C2230, 0 vs. 120min: $p<0.0001$	Combined across sex;<br><br>Sham + Veh (n=8)<br><br>SNI + Veh (n=10)<br><br>Sham + C2230 (n=8)<br><br>SNI + C2230 (n=10)
Figure 7C	SNI Chronic Aversion time	Two-Way repeated measures (RM) ANOVA<br><br>Time x Injury + Drug: $F(6,64)=46.76$	Dunnet's multiple comparisons test<br><br>Week 3, SNI + C2230, 0 vs. 120min:	Combined across sex;<br><br>Sham + Veh (n=8)

		p<0.0001<br><br>Time: F(1.745,55.84)=11.95 p<0.0001<br><br>Injury + Drug: F(3,32)=421.0 p<0.0001<br><br>Subject F(32,64)=3.263 p<0.0001	p<0.0001<br><br>Week 6, SNI + C2230, 0 vs. 120min: p<0.0001<br><br>Week 9, SNI + C2230, 0 vs. 120min: p<0.0001	SNI + Veh (n=10)<br><br>Sham + C2230 (n=8)<br><br>SNI + C2230 (n=10)
Figure 8K	SNI mice-Fiber photometry- AUC 0.07 g filament	Mixed-effects model (REML)<br><br>p=0.0725	Tukey's multiple comparisons test<br><br>BL vs. SNI: p=0.0046<br>BL vs. C2230 (10 mg/kg): p=0.5533<br>BL vs. vehicle: p=0.2678<br>SNI vs. C2230 (10 mg/kg): p=0.1806<br>SNI vs. vehicle: p=0.9995<br>C2230 (10 mg/kg) vs. vehicle: p=0.5218	Combined across sex; n=9-11
Figure 8L	SNI mice-Fiber photometry- peak ΔF/F 0.07 g filament	Mixed-effects model (REML)<br><br>p=0.0075	Tukey's multiple comparisons test<br><br>BL vs. SNI: p=0.0102<br>BL vs. C2230 (10 mg/kg): p=0.9991<br>BL vs. vehicle: p=0.3902<br>SNI vs. C2230 (10 mg/kg): p=0.0218<br>SNI vs. vehicle: p=0.3308<br>C2230 (10 mg/kg) vs. vehicle: p=0.5137	Combined across sex; n=9-11
Figure 8M	SNI mice-Fiber photometry- AUC 1.0 g filament	Mixed-effects model (REML)<br><br>p=0.0084	Tukey's multiple comparisons test<br><br>BL vs. SNI: p=0.0249<br>BL vs. C2230 (10 mg/kg): p=0.9753<br>BL vs. vehicle: p=0.4932<br>SNI vs. C2230 (10 mg/kg): p=0.0128	Combined across sex; n=9-11

			SNI vs. vehicle: p=0.4559<br>C2230 (10 mg/kg) vs. vehicle: p=0.3079	
Figure 8N	SNI mice-Fiber photometry- peak ΔF/F 1.0 g filament	Mixed-effects model (REML)<br><br>p=0.0013	Tukey's multiple comparisons test<br><br>BL vs. SNI: p=0.0029<br>BL vs. C2230 (10 mg/kg): p>0.9999<br>BL vs. vehicle: p=0.2355<br>SNI vs. C2230 (10 mg/kg): p=0.0046<br>SNI vs. vehicle: p=0.3004<br>C2230 (10 mg/kg) vs. vehicle: p=0.2655	Combined across sex; n=9-11
Figure 9B	SNL rats-Vehicle-Time in chamber	2-way RM ANOVA Chamber*time<br><br>Interaction: F(1,22)=97.30 p<0.0001<br><br>Chamber: F(1,22)= 24.00 p<0.0001<br><br>Time: p>0.9999 (NS)	Bonferroni multiple comparisons test between preconditioning and test.<br><br>vF: p<0.0001<br>NS: p<0.0001	Combined across sex; n=12; (6/6 males/females)
Figure 9C	SNL rats-C2230-Time in chamber	2-way RM ANOVA Chamber*time<br><br>Interaction: F(1,22)=9.019 p=0.0065<br><br>Chamber: F(1,22)= 8.539 p=0.0079<br><br>Time: p>0.9999 (NS)	Bonferroni multiple comparisons test between preconditioning and test.<br><br>vF: p=0.0904<br>NS: p=0.0904	Combined across sex; n=12; (6/6 males/females)
Figure 9D	SNL rats-CPA score	Unpaired t-test	Vehicle vs. C2230 (10 mg/kg): p=0.0358	Combined across sex; n=12 (6/6 males/females)
Figure 9E	SNL rats-Preconditioning distance	2-way ANOVA Sex*treatment<br><br>Interaction: p=0.7877 (NS)	Sidak's multiple comparisons test<br><br>Vehicle vs. C2230 (10 mg/kg)	n=6 for all groups.

		Sex:<br>p=0.4245 (NS)<br><br>Treatment:<br>p=0.5360 (NS)	Male: p=0.9613<br>Female: p=0.7796	
Figure 9F	SNL rats-Test distance	2-way ANOVA<br>Sex*treatment<br><br>Interaction:<br>p=0.3388 (NS)<br><br>Sex:<br>p=0.1364 (NS)<br><br>Treatment:<br>p=0.1794 (NS)	Sidak's multiple comparisons test<br><br>Vehicle vs. C2230 (10 mg/kg)<br><br>Male: p=0.5549<br>Female: p=0.9089	n=6 for all groups.
Figure 10C	Male CION rats-AUC Mechanical threshold	Mann-Whitney test	Vehicle vs. C2230 (200 µg/20 µl): p<0.0001	Vehicle (n=8)<br>C2230, 200 µg/20 µl (n=10)
Figure 10E	Female CION rats-AUC Mechanical threshold	Mann-Whitney test	Vehicle vs. C2230 (200 µg/20 µl): p=0.0001	Vehicle (n=10)<br>C2230, 200 µg/20 µl (n=10)
Figure 10G	Male CION rats-AUC pinprick response score	Mann-Whitney test	Vehicle vs. C2230 (200 µg/20 µl): p=0.0006	Vehicle (n=8)<br>C2230, 200 µg/20 µl (n=10)
Figure 10I	Female CION rats-AUC pinprick response score	Mann-Whitney test	Vehicle vs. C2230 (200 µg/20 µl): p<0.0001	Vehicle (n=10)<br>C2230, 200 µg/20 µl (n=10)
Figure 11C	Male MIA mice-AUC Mechanical threshold	Unpaired t test	Vehicle vs. C2230 (10 mg/kg): p=0.0473	Vehicle (n=7)<br>C2230, 10 mg/kg (n=6)
Figure 11E	Female MIA mice-AUC Mechanical threshold	Unpaired t test	Vehicle vs. C2230 (10 mg/kg): p<0.0001	Vehicle (n=6)<br>C2230, 10 mg/kg (n=7)
Figure 11G	Male MIA mice-AUC cold aversion time	Unpaired t test	Vehicle vs. C2230 (10 mg/kg): p=0.0529	Vehicle (n=7)<br>C2230, 10 mg/kg (n=6)
Figure 11I	Female MIA mice-AUC cold aversion time	Unpaired t test	Vehicle vs. C2230 (10 mg/kg): p=0.0051	Vehicle (n=6)<br>C2230, 10 mg/kg (n=7)
Fig 11G+I Additional analysis for sex-differences	Female and male MIA, AUC, cold aversion time	2-way ANOVA, Sex*treatment<br><br>Sex; F(1,22)=5.592 p=0.0273<br><br>Treatment; F(1,22)=16.04 p=0.0006<br><br>Interaction; F(1,22)=0.922 p=0.347	Sidak's multiple comparison;<br>Vehicle vs. C2230 (10 mg/kg):<br><br>Males; p=0.0832 (NS)<br>Female; p=0.0039	Females;<br>Vehicle (n=6)<br>C2230, 10 mg/kg (n=7)<br><br>Males;<br>Vehicle (n=7)<br>C2230, 10 mg/kg (n=6)

Figure 12B	Male mechanical threshold-Naïve mice	2-way ANOVA<br>Treatment*Time<br><br>Interaction:<br>F(6,98)=0.4410<br>p=0.8497<br><br>Time:<br>F(6,98)=1.039<br>p=0.4048<br><br>Treatment:<br>F(1,98)=0.3513<br>p=0.5548	Bonferroni's multiple comparisons test<br><br>DMSO vs. C2230 (30 mg/kg)<br><br>0 h: p>0.9999<br>1 h: p>0.9999<br>2 h: p>0.9999<br>3 h: p>0.9999<br>4 h: p>0.9999<br>5 h: p>0.9999<br>6 h: p>0.9999	Vehicle (n=8)<br>C2230, 30 mg/kg (n=8)
Figure 12C	Male aversion time-Naïve mice	2-way ANOVA<br>Treatment*Time<br><br>Interaction:<br>F(6,98)=0.1897<br>p=0.9791<br><br>Time:<br>F(6,98)=0.3332<br>p=0.9179<br><br>Treatment:<br>F(1,98)=0.7495<br>p=0.7595	Bonferroni's multiple comparisons test<br><br>DMSO vs. C2230 (30 mg/kg)<br><br>0 h: p>0.9999<br>1 h: p>0.9999<br>2 h: p>0.9999<br>3 h: p>0.9999<br>4 h: p>0.9999<br>5 h: p>0.9999<br>6 h: p>0.9999	Vehicle (n=8)<br>C2230, 30 mg/kg (n=8)
Figure 12D	Male hot plate latency	Mann-Whitney test	DMSO vs. C2230 (30 mg/kg)<br>p=0.5540	Vehicle (n=8)<br>C2230, 30 mg/kg (n=8)
Figure 12E	Male rotarod latency to fall	Mann-Whitney test	DMSO vs. C2230 (30 mg/kg)<br>p>0.9999	Vehicle (n=8)<br>C2230, 30 mg/kg (n=8)
Figure 12F	Female mechanical threshold-Naïve mice	2-way ANOVA<br>Treatment*Time<br><br>Interaction:<br>F(6,91)=0.1497<br>p=0.9887<br><br>Time:<br>F(6,91)=0.2271<br>p=0.9669<br><br>Treatment:<br>F(1,91)=3.060<br>p=0.0836	Bonferroni's multiple comparisons test<br><br>DMSO vs. C2230 (30 mg/kg)<br><br>0 h: p>0.9999<br>1 h: p>0.9999<br>2 h: p>0.9999<br>3 h: p>0.9999<br>4 h: p>0.9999<br>5 h: p>0.9999<br>6 h: p>0.9999	Vehicle (n=7)<br>C2230, 30 mg/kg (n=8)
Figure 12G	Female aversion time-Naïve mice	2-way ANOVA<br>Treatment*Time<br><br>Interaction:<br>F(6,91)=0.1732<br>p=0.9834<br><br>Time:	Bonferroni's multiple comparisons test<br><br>DMSO vs. C2230 (30 mg/kg)<br><br>0 h: p>0.9999<br>1 h: p>0.9999	Vehicle (n=7<br>C2230, 30 mg/kg (n=8)

		F(6,91)=0.4424<br>p=0.8486<br><br>Treatment:<br>F(1,91)=0.8668<br>p=0.7595	2 h: p>0.9999<br>3 h: p>0.9999<br>4 h: p>0.9999<br>5 h: p>0.9999<br>6 h: p>0.9999	
Figure 12H	Female hot plate latency	Mann-Whitney test	DMSO vs. C2230 (30 mg/kg)<br>p=0.7789	Vehicle (n=7)<br>C2230, 30 mg/kg (n=8)
Figure 12I	Female rotarod latency to fall	Mann-Whitney test	DMSO vs. C2230 (30 mg/kg)<br>p>0.9999	Vehicle (n=7)<br>C2230, 30 mg/kg (n=8)
Figure 13D	Inhibition ratio DIII S5	Kruskal-Wallis test<br><br>p<0.0001	WT vs. N1281A: p>0.9999<br>WT vs. I1282A: p=0.4033<br>WT vs. L1283A: p>0.9999<br>WT vs. I1284A; p>0.9999<br>WT vs. V1285A: p>0.9999<br>WT vs. Y1286A: p>0.9999<br>WT vs. M1287A: p>0.9999<br>WT vs. L1288A: p=0.0010<br>WT vs. F1289A: p=0.6603<br>WT vs. F1291A: p>0.9999<br>WT vs. I1292A: p>0.9999<br>WT vs. F1293A: p=0.2014<br>WT vs. A1294G: p=0.01606<br>WT vs. V1295A: p>0.9999<br>WT vs. I1296Ap=0.1555<br>WT vs. A1297G: p>0.9999<br>WT vs. L1300A: p>0.9999	WT (n=12)<br>N1281A (n=5)<br>I1282A (n=4)<br>L1283A (n=5)<br>I1284A (n=5)<br>V1285A (n=5)<br>Y1286A (n=5)<br>M1287A (n=5)<br>L1288A (n=5)<br>F1289A (n=5)<br>F1291A (n=5)<br>I1292A (n=5)<br>F1293A (n=6)<br>A1294G (n=5)<br>V1295A (n=5)<br>I1296A (n=5)<br>A1297G (n=5)<br>L1300A (n=5)
Figure 13E	Inhibition ratio DIV S6	Kruskal-Wallis test<br><br>p<0.0001	WT vs. A1681G: p>0.9999<br>WT vs. Y1682A: p>0.9999<br>WT vs. F1683A: p=0.0154<br>WT vs. Y1684A: p=0.2083<br>WT vs. S1687A: p>0.9999	WT (n=12)<br>A1681G (n=5)<br>Y1682A (n=5)<br>F1683A (n=6)<br>Y1684A (n=5)<br>S1687A (n=5)<br>I1689N (n=5)<br>F1690A (n=6)<br>C1692A (n=6)<br>S1693A (n=5)

			WT vs. I1689N: p>0.9999<br>WT vs. F1690A: p>0.9999<br>WT vs. C1692A: p>0.9999<br>WT vs. S1693A: p>0.9999<br>WT vs. L1695A: p>0.9999<br>WT vs. M1696A: p>0.9999<br>WT vs. L1697A: p>0.9999<br>WT vs. L1699A: p>0.9999<br>WT vs. F1700C: p=0.3570<br>WT vs. V1701F: p>0.9999<br>WT vs. A1702G: p>0.9999<br>WT vs. V1703A: p>0.9999	L1695A (n=5)<br>M1696A (n=6)<br>L1697A (n=6)<br>L1699A (n=5)<br>F1700C (n=5)<br>V1701F (n=5)<br>A1702G (n=5)<br>V1703A (n=5)
Figure 13F	Inhibition ratio DIII S6	Kruskal-Wallis test<br><br>p<0.0001	WT vs. E1388A: p>0.9999<br>WT vs. L1389A: p>0.9999<br>WT vs. S1390A: p=0.0239<br>WT vs. I1391A: p>0.9999<br>WT vs. F1392A: p>0.9999<br>WT vs. Y1393A: p>0.9999<br>WT vs. V1394A: p>0.9999<br>WT vs. Y1396A: p>0.9999<br>WT vs. F1397A: p>0.9999<br>WT vs. V1398A: p>0.9999<br>WT vs. V1399A: p=0.7594<br>WT vs. F1400A: p>0.9999<br>WT vs. P1401A: p>0.9999<br>WT vs. F1402A: p>0.9999<br>WT vs. F1403A: p>0.9999<br>WT vs. F1404A: p=0.0072	WT (n=12)<br>E1388A (n=4)<br>L1389A (n=4)<br>S1390A (n=5)<br>I1391A (n=5)<br>F1392A (n=5)<br>Y1393A (n=5)<br>V1394A (n=5)<br>Y1396A (n=3)<br>F1397A (n=4)<br>V1398A (n=4)<br>V1399A (n=5)<br>F1400A (n=5)<br>P1401A (n=5)<br>F1402A (n=5)<br>F1403A (n=5)<br>F1404A (n=6)<br>V1405A (n=5)<br>N1406A (n=5)<br>I1407A (n=5)<br>F1408A (n=5)<br>A1410G (n=5)<br>L1411A (n=5)<br>I1412A (n=5)<br>I1413A (n=5)

			WT vs. V1405A: p>0.9999<br>WT vs. N1406A: p>0.9999<br>WT vs. I1407A: p>0.9999<br>WT vs. F1408A: p>0.9999<br>WT vs. A1410G: p>0.9999<br>WT vs. L1411A: p>0.9999<br>WT vs. I1412A: p>0.9999<br>WT vs. I1413A: p=0.1339	
Supplementary figure 4A	RMP	Mann-Whitney test	Vehicle vs. C2230 (20 μM): p=0.3691	Vehicle (n=12) C2230 (n=10)
Supplementary figure 4B	Rheobase	Mann-Whitney test	Vehicle vs. C2230 (20 μM): p=0.0549	Vehicle (n=12) C2230 (n=10)
Supplementary figure 4D	Excitability 2X rheobase	Mann-Whitney test	Vehicle vs. C2230 (20 μM): p=0.3342	Vehicle (n=12) C2230 (n=10)
Supplementary figure 4E	fAHP	Mann-Whitney test	Vehicle vs. C2230 (20 μM): p=0.1746	Vehicle (n=12) C2230 (n=10)
Supplementary figure 6C	Male SNL rats-AUC PWT	Mann-Whitney test	Vehicle vs. C2230 (10 μg/5 μl): p=0.0260	Vehicle (n=6) C2230, 10 μg/5 μl (n=6)
Supplementary figure 7B	Male naïve mice-MABP	Unpaired t test	Vehicle vs. C2230 (10 mg/kg): p=0.1845	Vehicle (n=4) C2230 (n=5)
Supplementary figure 7C	Male naïve mice-MHR	Unpaired t test	Vehicle vs. C2230 (10 mg/kg): p=0.0880	Vehicle (n=4) C2230 (n=5)

Supplemental References

1. Tang D, Yang Y, Xiao Z, Xu J, Yang Q, Dai H, et al. Scorpion toxin inhibits the voltage-gated proton channel using a Zn(2+) -like long-range conformational coupling mechanism. *Br J Pharmacol.* 2020;177(10):2351-64.
2. Francois-Moutal L, Dustrude ET, Wang Y, Brustovetsky T, Dorame A, Ju W, et al. Inhibition of the Ubc9 E2 SUMO-conjugating enzyme-CRMP2 interaction decreases NaV1.7 currents and reverses experimental neuropathic pain. *Pain.* 2018;159(10):2115-27.

3. Moutal A, Martin LF, Boinon L, Gomez K, Ran D, Zhou Y, et al. SARS-CoV-2 spike protein co-opts VEGF-A/neuropilin-1 receptor signaling to induce analgesia. *Pain.* 2021;162(1):243-52.

4. Dustrude ET, Moutal A, Yang X, Wang Y, Khanna M, and Khanna R. Hierarchical CRMP2 posttranslational modifications control NaV1.7 function. *Proc Natl Acad Sci U S A.* 2016;113(52):E8443-E52.

5. Piekarz AD, Due MR, Khanna M, Wang B, Ripsch MS, Wang R, et al. CRMP-2 peptide mediated decrease of high and low voltage-activated calcium channels, attenuation of nociceptor excitability, and anti-nociception in a model of AIDS therapy-induced painful peripheral neuropathy. *Mol Pain.* 2012;8:54.

6. Xie JY, Chew LA, Yang X, Wang Y, Qu C, Wang Y, et al. Sustained relief of ongoing experimental neuropathic pain by a CRMP2 peptide aptamer with low abuse potential. *Pain.* 2016;157(9):2124-40.

7. Basbaum AI, Bautista DM, Scherrer G, and Julius D. Cellular and molecular mechanisms of pain. *Cell.* 2009;139(2):267-84.

8. Hartung JE, Moy JK, Loeza-Alcocer E, Nagarajan V, Jostock R, Christoph T, et al. Voltage-gated calcium currents in human dorsal root ganglion neurons. *Pain.* 2022;163(6):e774-e85.

9. Ye GL, Savelieva KV, Vogel P, Baker KB, Mason S, Lanthorn TH, et al. Ligation of mouse L4 and L5 spinal nerves produces robust allodynia without major motor function deficit. *Behav Brain Res.* 2015;276:99-110.

10. Ho Kim S, and Mo Chung J. An experimental model for peripheral neuropathy produced by segmental spinal nerve ligation in the rat. *Pain.* 1992;50(3):355-63.

11. Nelson TS, Sinha GP, Santos DFS, Jukkola P, Prasoon P, Winter MK, et al. Spinal neuropeptide Y Y1 receptor-expressing neurons are a pharmacotherapeutic target for the alleviation of neuropathic pain. *Proc Natl Acad Sci U S A.* 2022;119(46):e2204515119.

12. Imamura Y, Kawamoto H, and Nakanishi O. Characterization of heat-hyperalgesia in an experimental trigeminal neuropathy in rats. *Exp Brain Res.* 1997;116(1):97-103.

13. Pitcher T, Sousa-Valente J, and Malcangio M. The Monoiodoacetate Model of Osteoarthritis Pain in the Mouse. *J Vis Exp.* 2016(111).

14. Chaplan SR, Bach FW, Pogrel JW, Chung JM, and Yaksh TL. Quantitative assessment of tactile allodynia in the rat paw. *J Neurosci Methods.* 1994;53(1):55-63.

15. Liu CY, Lu ZY, Li N, Yu LH, Zhao YF, and Ma B. The role of large-conductance, calcium-activated potassium channels in a rat model of trigeminal neuropathic pain. *Cephalalgia.* 2015;35(1):16-35.

16. Benoliel R, Wilensky A, Tal M, and Eliav E. Application of a pro-inflammatory agent to the orbital portion of the rat infraorbital nerve induces changes indicative of ongoing trigeminal pain. *Pain.* 2002;99(3):567-78.

17. Vos BP, Strassman AM, and Maciewicz RJ. Behavioral evidence of trigeminal neuropathic pain following chronic constriction injury to the rat's infraorbital nerve. *J Neurosci.* 1994;14(5 Pt 1):2708-23.

18. Zhou H, Zhang Q, Martinez E, Dale J, Hu S, Zhang E, et al. Ketamine reduces aversion in rodent pain models by suppressing hyperactivity of the anterior cingulate cortex. *Nat Commun.* 2018;9(1):3751.

19. Sharma RK, Yang T, Oliveira AC, Lobaton GO, Aquino V, Kim S, et al. Microglial Cells Impact Gut Microbiota and Gut Pathology in Angiotensin II-Induced Hypertension. *Circ Res.* 2019;124(5):727-36.

20. Gupta M, Lee HJ, Barden CJ, and Weaver DF. The Blood-Brain Barrier (BBB) Score. *J Med Chem.* 2019;62(21):9824-36.

21. Lipinski CA. Lead- and drug-like compounds: the rule-of-five revolution. *Drug Discov Today Technol.* 2004;1(4):337-41.

22. Bickerton GR, Paolini GV, Besnard J, Muresan S, and Hopkins AL. Quantifying the chemical beauty of drugs. *Nat Chem.* 2012;4(2):90-8.
